# Quantitative parameters of bacterial RNA polymerase open-complex formation, stabilization and disruption on a consensus promoter

**DOI:** 10.1093/nar/gkac560

**Published:** 2022-07-12

**Authors:** Subhas C Bera, Pim P B America, Santeri Maatsola, Mona Seifert, Eugeniu Ostrofet, Jelmer Cnossen, Monika Spermann, Flávia S Papini, Martin Depken, Anssi M Malinen, David Dulin

**Affiliations:** Junior Research Group 2, Interdisciplinary Center for Clinical Research, Friedrich Alexander University Erlangen-Nürnberg (FAU), Cauerstr. 3, 91058 Erlangen, Germany; Department of Physics and Astronomy, and LaserLaB Amsterdam, Vrije Universiteit Amsterdam, De Boelelaan 1081, 1081 HV, Amsterdam, The Netherlands; Department of Life Technologies, University of Turku, Tykistökatu 6A, 6th floor, 20520 Turku, Finland; Junior Research Group 2, Interdisciplinary Center for Clinical Research, Friedrich Alexander University Erlangen-Nürnberg (FAU), Cauerstr. 3, 91058 Erlangen, Germany; Junior Research Group 2, Interdisciplinary Center for Clinical Research, Friedrich Alexander University Erlangen-Nürnberg (FAU), Cauerstr. 3, 91058 Erlangen, Germany; Delft Center for Systems and Control, Delft University of Technology, Delft, the Netherlands; Junior Research Group 2, Interdisciplinary Center for Clinical Research, Friedrich Alexander University Erlangen-Nürnberg (FAU), Cauerstr. 3, 91058 Erlangen, Germany; Junior Research Group 2, Interdisciplinary Center for Clinical Research, Friedrich Alexander University Erlangen-Nürnberg (FAU), Cauerstr. 3, 91058 Erlangen, Germany; Department of Bionanoscience, Kavli Institute of Nanoscience, Delft University of Technology, Van der Maasweg 9, 2629 HZ Delft, The Netherlands; Department of Life Technologies, University of Turku, Tykistökatu 6A, 6th floor, 20520 Turku, Finland; Junior Research Group 2, Interdisciplinary Center for Clinical Research, Friedrich Alexander University Erlangen-Nürnberg (FAU), Cauerstr. 3, 91058 Erlangen, Germany; Department of Physics and Astronomy, and LaserLaB Amsterdam, Vrije Universiteit Amsterdam, De Boelelaan 1081, 1081 HV, Amsterdam, The Netherlands

## Abstract

Transcription initiation is the first step in gene expression, and is therefore strongly regulated in all domains of life. The RNA polymerase (RNAP) first associates with the initiation factor $\sigma$ to form a holoenzyme, which binds, bends and opens the promoter in a succession of reversible states. These states are critical for transcription regulation, but remain poorly understood. Here, we addressed the mechanism of open complex formation by monitoring its assembly/disassembly kinetics on individual consensus *lacUV5* promoters using high-throughput single-molecule magnetic tweezers. We probed the key protein–DNA interactions governing the open-complex formation and dissociation pathway by modulating the dynamics at different concentrations of monovalent salts and varying temperatures. Consistent with ensemble studies, we observed that RNAP-promoter open (RP_O_) complex is a stable, slowly reversible state that is preceded by a kinetically significant open intermediate (RP_I_), from which the holoenzyme dissociates. A strong anion concentration and type dependence indicates that the RP_O_ stabilization may involve sequence-independent interactions between the DNA and the holoenzyme, driven by a non-Coulombic effect consistent with the non-template DNA strand interacting with $\sigma$ and the RNAP $\beta$ subunit. The temperature dependence provides the energy scale of open-complex formation and further supports the existence of additional intermediates.

## INTRODUCTION

DNA-dependent RNA polymerase (RNAP) is the molecular machine responsible for the RNA production, i.e. the first step of gene expression, in cells (1,2). The structural core of RNAP is conserved in bacteria, eukaryotes and archaea (3–5). RNAP is a versatile target for antimicrobials having more than ten distinct small-molecule binding sites (6), such as rifamycins and fidaxomycin to treat either tuberculosis or *Clostridium difficile*-associated diarrhea, respectively. The bacterial RNAP, typically consisting of five subunits (ααββ’ω), is structurally the simplest multi-subunits RNAP and has been widely employed as model system for molecular mechanisms of transcription and transcription regulation. The core RNAP cannot initiate promoter-specific transcription on its own. This deficit is compensated in bacteria by σ factors, which bind to the RNAP to form the transcription initiation competent RNAP holoenzyme complex (one σ per RNAP) (1,7).

Biochemical, structural and single-molecule studies have defined the basics of the transcription initiation process by bacterial RNAP–σ^70^ holoenzyme (from now on referred to as holo, and reviewed in (8,9)). This multistep mechanism begins with holo searching for promoters, embedded in the vast excess of non-promoter DNA. This search probably proceeds by a combination of modes, i.e. holo sliding along the DNA duplex (10–15), holo hopping from a DNA binding site to another nearby site (16) and holo diffusing through the bulk solution (17). The holo docks on the promoter via concerted interactions with specific promoter regions known as UP (from around –60 to –40 region of the promoter relative to transcription start site at +1 position), –35, spacer and –10 elements (8,18–22). This initial unstable RNAP–promoter complex (RP_C_, where no DNA melting has occurred) isomerizes to more stable forms when the upstream and downstream regions of the promoter bend along the RNAP surface and into the DNA binding cleft, respectively (23–26). The formation of catalytically active holo–promoter open complex (RP_O_) is completed when the –11/+2 region of the promoter DNA duplex unwinds and the template DNA strand enters the active site cleft of the RNAP (27–30). The non-template (nt) DNA remains trapped outside the active site by the interactions between the –10 element and σ^70^ region 2, with –11A and –7T being flipped from the ntDNA base stack to deep pockets in σ^70^ (28,31). ntDNA binding is further stabilized by the discriminator (-6/+1) interactions with the σ^70^ region 2 and the core recognition element with the RNAP $\beta$ subunit (27). Promoter sequence, transcription factors and small solutes modulate the stabilities and interconversion rates of the intermediates on the RP_O_ formation pathway and thus the level of gene expression (8).

Several studies have implied that not all formed RP_O_’s are structurally and functionally identical. Most biochemical studies report the existence of two (32–34) or three (35) open complex structures (RP_O_, intermediates), but also several closed complex intermediates have recently been identified (26). A recent cryoEM study from Darst and colleagues reported seven intermediate states towards RP_O_ formation (36). In most cases, less than half of the apparent RP_O_’s appear capable of productive promoter escape followed by full-length RNA synthesis (32,37–40). The majority of RP_O_ instead get trapped at the promoter and only synthesize short RNAs, and such moribund complexes (32) appear to play a role in transcription regulation (41). What these RP_O_ intermediates are, what interactions determine them, and what mechanisms allow their interconversion, remain poorly understood.

Single-molecule studies have revealed long-lived pausing and backtracking during transcription initiation (40,42). Instead of predefined RP_O_ populations producing different RNA types, a single RP_O_ could enter a pause/backtrack during initial RNA synthesis, before stochastically escaping the promoter. Controversy in data remains as a magnetic tweezers based single-molecule study reported only a single uniform RP_O_ (43), whereas a smFRET based study reported an additional low occupancy RP_O_ species with an unstable promoter conformation (44).

In the present study, we addressed the mechanism and heterogeneity of RP_O_ formation using high-throughput magnetic tweezers by monitoring the kinetics of RP_O_ formation and dissociation on the consensus *lacUV5* promoter, a model system to study open complex dynamics. We probed the key protein–DNA interactions governing the RP_O_ pathway by modulating the dynamics with monovalent salts as well as temperature. We observed two different open conformations, i.e. one intermediate open complex RP_I_ and one final open state RP_O_, and we report here the rate constants between these two states. We show that the identity of monovalent cation mainly affects DNA twist, whereas the ranking of the anion in the Hofmeister series correlates with its effect on the transition from RP_C_ to RP_I_, indicating that this transition is driven by non-Coulombic interactions. Specifically, while physiological glutamate concentration favors rapid open complex formation, similar concentration of chloride promotes direct holo dissociation from the RP_I_ state to the degree that it does not populate the RP_O_ state anymore. From the strong salt dependence, we suggest that the stabilization of RP_O_ involves sequence-independent interactions between the DNA and the holo. One such candidate network of interaction takes place between the holo and the discriminator region of the promoter. Finally, the temperature dependence investigation revealed the energy landscape of open complex formation, the free energy difference between RP_C_ and dissociated holo, and supports the existence of several intermediate states during dissociation.

## MATERIALS AND METHODS

### High throughput magnetic tweezers

**Figure 1.**
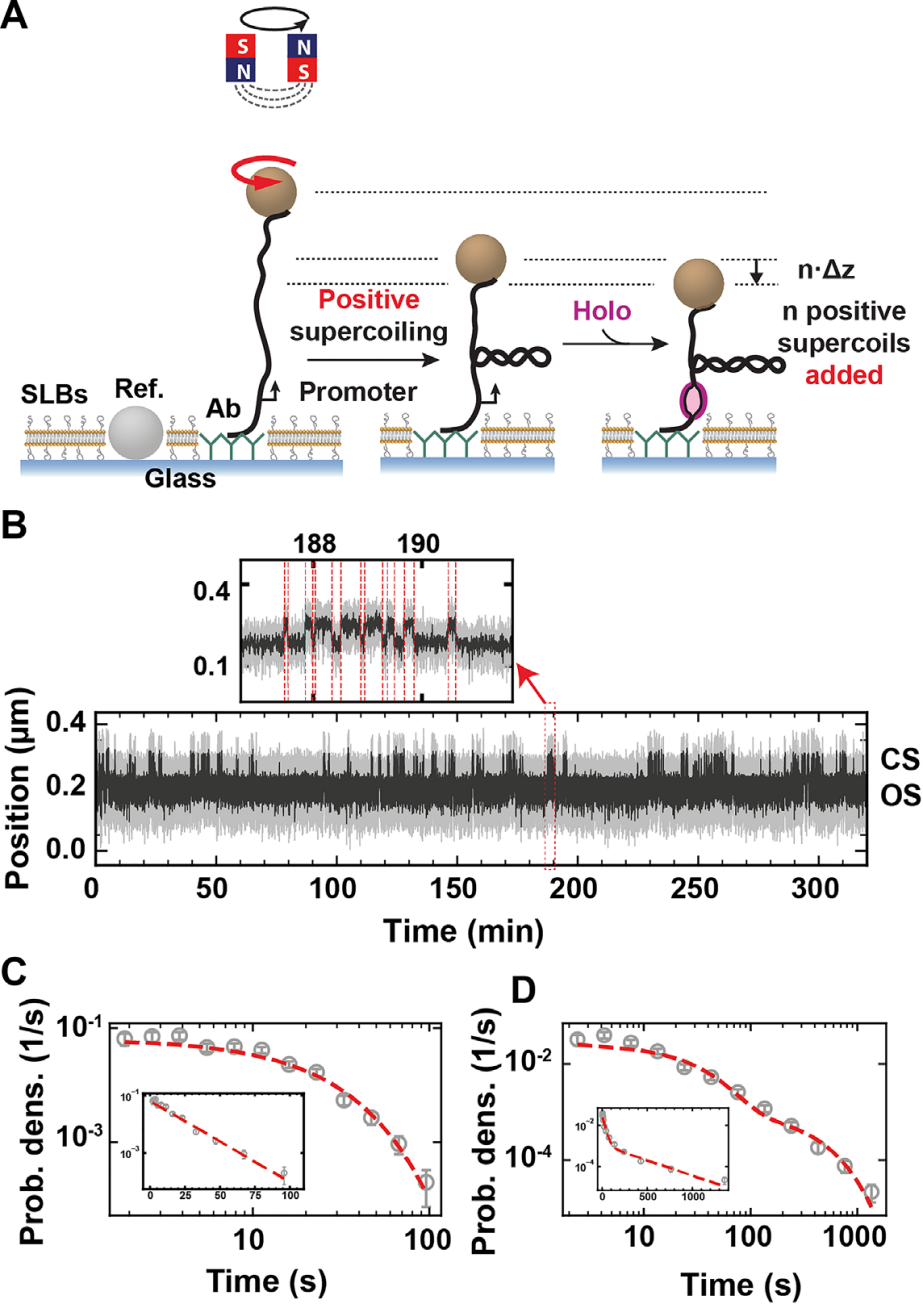
Measuring the bacterial holo open-complex dynamics using magnetic tweezers. (**A**) DNA plectonems formation upon positive supercoiling. Open complex formation by the holo leads to a decrease in the end-to-end extension of the DNA molecule by *n*.Δ*z*, with *n* being the number of open base-pairs and Δ*z* the distance by which the DNA extension decreases per full turn addition in a rotation extension experiment (Supplementary Figure S1C), i.e. Δ*z* ∼ 60 nm in reaction buffer. (**B**) Typical experimental trace showing dynamic transition between a promoter open and closed state (OS and CS, respectively) as described in (A). The experiment was performed with 10 nM holo, 150 mM KAc and at 34°C. A zoom in the trace shows fast open-complex dynamics. Raw data at 58 Hz (grey) and 10-times average (black). The red dashed lines indicate the transitions between the OS and CS captured by the change-point analysis (Materials and Methods). (**C**) Probability density distribution of the CS dwell times for traces acquired as described in (B). The dashed red line is a maximum likelihood estimation (MLE) fit for a single exponential probability distribution function. Inset shows the same plot in log-linear scale. (**D**) Probability density distribution of the OS dwell time distribution for traces acquired as described in (B). The dashed red line is a MLE fit for a double exponential probability distribution function. Inset shows the same plot in log-linear scale. Error bars in (C, D) are two standard deviations extracted from 1000 bootstraps.

We used the high-throughput magnetic tweezers apparatus previously described in (45–47) to monitor 30–100 individual DNA tethers in parallel. In short, it is a custom inverted microscope with a 50× oil immersion objective (CFI Plan Achro 50 XH, NA 0.9, Nikon, Germany), on top of which a flow chamber is mounted. The streptavidin coated magnetic beads (1 μm MyOne, ThermoFisher, Cat # 65001) are tethered to the bottom of the glass coverslip by a DNA construct that contains the *lacCONS +2* promoter for *Escherichia coli* RNA polymerase (see *DNA construct fabrication*) (40) (Figure 1). A typical field of view is shown in Supplementary Figure S1A with 500–700 tethers and a few reference beads. An attractive force is applied to the magnetic beads to stretch the nucleic acid tether (Figure 1A) using a pair of vertically aligned permanent magnets (5 mm cubes, SuperMagnete, Switzerland). The magnets are separated by a 1 mm gap and are positioned above the objective as described in Ref. (45). The vertical position and rotation of the beads are controlled by the M-126-PD1 and C-150 motors (Physik Instrumente PI, GmbH & Co. KG, Karlsruhe, Germany), respectively. The field of view is illuminated through the magnets’ gap by a collimated LED-light source located above, and is imaged onto a large sensor CMOS camera (Dalsa Falcon2 FA-80-12M1H, Stemmer Imaging, Germany). All data were recorded at 58 Hz acquisition frequency. For the change-point analysis, (see *Dwell times detection*) the raw data were averaged 10-times.

### Preparation of vesicles

Small unilamellar vesicles (SUVs) were prepared by mixing DOPC (1,2-dioleoyl-sn-glycero-3-phosphocholine) and PEG-PE (1,2-dioleoyl-sn-glycero-3-phosphoethanolamine-*N*-[methoxy(polyethylene glycol)-550]) (850375C, 880530C, respectively, Avanti Polar Lipids, USA) dissolved in chloroform in 95:5 molar fraction. After mixing, the solution was dried under a stream of nitrogen, followed by drying in a vacuum desiccator for 1 h. The dry lipid film was then resuspended at 4 mg/ml in the storage buffer (10 mM HEPES, pH 7.8, 150 mM NaCl, 2 mM EDTA, 2 mM sodium azide) by vortexing. Then the suspension was incubated for 1 h at room temperature, with occasional vortexing and subsequently extruded 21 times through 0.1 μm polycarbonate membrane (Avanti, Cat # 610005) using a mini-extruder (Avanti Polar Lipids, USA, Cat # 610023). SUVs stock solution was stored at +4ºC for several weeks.

### Flow cell assembly and lipid bilayer preparation

We used a lipid bilayer passivation strategy in the RP_O_ formation experiments, as our standard nitrocellulose passivation (45) failed to prevent non-specific sticking of the magnetic beads to the coverslip surface in the presence of holo. The glass coverslips used to assemble the flow cell (#1 thickness, 24 × 60 mm MenzelGlazer, Germany) were washed by sonication in a solution of Hellmanex III (Sigma-Aldrich, Germany) diluted in demineralized water (1%, v/v). The coverslips were subsequently rinsed thoroughly under a stream of demineralized water, dried in an oven at 80°C, and stored in a 50 ml Falcon tube. A diluted stock of 1 μm polystyrene reference beads were prepared by diluting 1 μl stock (Sigma, Cat # LB11) 500-fold in mQ water, and rinsing them by repeating three times the following procedure: vortex, centrifugation at ∼5000 rpm on a benchtop centrifuge for ∼1 min, removal of the supernatant and resuspension in the same volume of mQ water. The washed reference beads are finally resuspended in 50 μl mQ water and stored at 4°C for further use. ∼4 μl of the reference bead solution, obtained by diluting the secondary stock 10-fold in absolute ethanol, was then spread with the side of a pipet tip on the top surface of the bottom coverslip of the flow cell, and subsequently heated to ∼130°C for ∼2 min to melt the reference beads on the coverslip surface. Preceding the assembly of the flow cell, the side of the coverslips that forms the inner channel of the flow cell, were activated and made hydrophilic by thoroughly treating their surface with the electric discharge originating from a Corona SB (BlackHole Lab, Paris, France). The flow cell was then assembled by sandwiching a double layer of Parafilm (Sigma Aldrich, Germany, Cat # P7793) between two coverslips treated as described above. The flow cell was sealed by melting the Parafilm on a hot plate at ∼100°C for 1 min, while firmly pressing on top. The flow cell was subsequently mounted on the magnetic tweezers setup and rinsed with 1 ml 1× Phosphate buffered saline (PBS). 50 μl of full-length anti-digoxigenin (0.5 mg/ml in PBS, Sigma Aldrich, Germany, Cat # 11333089001) were added and incubated for 30 min. The excess was subsequently flushed away with 1 ml of 1× PBS buffer containing 700 mM NaCl followed by 10 min incubation, and a rinsing step with 1 ml PBS. The buffer in the flow cell was exchanged with 1 ml vesicle dilution buffer (10 mM HEPES at pH 7.4, 150 mM NaCl, 2 mM EDTA, 2 mM sodium azide and 2 mM CaCl_2_) to prepare the lipid bilayer assembly. The lipid bilayer was formed by flushing 1.2 ml of the SUV solution (see ‘*Preparation of vesicles*’) at 50 μg/ml in the vesicle dilution buffer (Sigma Aldrich, Germany), slowly through the flow cell at 0.1 ml/min for a total duration of ∼15 min. The flow channel was then washed with 1 ml PBS to remove any excess SUVs. To finalize the surface passivation, bovine serum albumin (BSA) (New England Biolabs, USA) at 1 mg/ml in PBS was incubated for 30 min, and subsequently flushed away excess BSA with 1 ml PBS.

In the meantime, 10 μl of MyOne streptavidin-coated superparamagnetic Dynabeads (Thermofisher, Germany, Cat # 65604D) were washed twice in PBS and diluted to 40 μl of PBS mixed with ∼15 pM of DNA construct and 1 mg/ml BSA and incubated for a few minutes. The DNA tethered magnetic beads were then flushed into the flow cell and incubated for ∼5 min to ensure attachment of the digoxygenin-labelled DNA handle to the anti-digoxigenin adsorbed on the flow cell surface. Finally, the excess of magnetic beads was removed by flushing copious amounts of PBS with occasional gentle tapping on the exit tube connecting the flow cell to the withdrawing pump.

### DNA construct fabrication

The long linear DNA construct (20 666 bp, sequence in Supplementary Data 1) was obtained by plasmid digestion and ligation with functionalized digoxigenin- or biotin-handles (850 bp), obtained from PCR on λ DNA (45). The fabrication of the 1.4 kb DNA construct (sequence in Supplementary Data 2) was done as described in (48). Briefly, the desired DNA fragments were amplified by PCR from a synthetic plasmid containing the *E. coli* RNAP *LacCONS* promoter sequence and selectively cleaved by nicking enzymes at multiple sites to obtain ssDNA of different lengths and partial complementarity. These fragments were annealed to form a double strand with functionalized biotin and digoxygenin handles. The resulting nicks were ligated to obtain a torsionally constrained molecule.

### Protein purification and assembly

*Escherichia coli* RNAP and σ^70^ were expressed in *E. coli* and purified by using sequentially nickel affinity, heparin affinity and anion exchange chromatography steps as previously described in (49). Holoenzymes were assembled by incubating RNAP with 3-fold molar excess of σ^70^ for 30 min at 30°C as described in (40).

### DNA tether selection

We first evaluated the extension and coilability of the DNA molecule by stretching the DNA from zero to 4 pN and rotating the magnetic bead first in negative and then in positive directions (±15–100 turns depending on the length of tether), while applying 4 pN force. For a coilable and single-tether DNA molecule, the extension should remain constant in the negative turn region but decrease in the positive turn region (50).

### DNA rotation experiment in different monovalent salts

To determine the effect of monovalent salt on DNA twist, we used a coilable ∼20.6 kb DNA molecule, fabrication of the construct was described elsewhere (51,52). After selection of coilable DNA molecules, the flow cell was flushed with a reference buffer (10 mM Tris–HCl, 150 mM NaCl, 2 mM EDTA) with occasional mild tapping (without mounting the magnets) that allows the DNA to release any unwanted twist. The rotation experiments were performed by slowly rotating the magnets (2 turns/s) from −70 to 70 turns at ∼0.3 pN force, the zero twist in the tethers being defined by the maximum extension in the reference buffer. The buffer (10 mM Tris−HCl, 2 mM EDTA) containing the monovalent salt of interest was flushed in the flow cell while applying a high force to the DNA (8 pN) and at zero turn. After 3 min of incubation, the same rotation experiment was performed in the experiment buffer containing the monovalent salt. Finally, the flow cell was flushed with the reference buffer while clamping the DNA with high force (8 pN) and after 3 min of incubation, another measurement was performed in the reference buffer. The first and the last reference measurement were performed to check whether the DNA supercoiled state had been restored in reference buffer to eliminate tethers in which a change in twist may have occurred from the magnetic bead sticking to the surface. When changing salt condition, the flow cell was flushed in with the reference buffer without the magnet to allow the DNA to relax and the same procedure was repeated. These experiments were performed at 25°C.

### Magnetic tweezers holo open complex dynamics experiments

To monitor the open complex dynamics, we tethered magnetic beads with the ∼1.4 kb coilable DNA molecules. The flow cell was rinsed with reaction buffer (40 mM HEPES, 10 mM MgAc_2_, 1 mM DTT, 1 mM cysteamine hydrochloride, 5% glycerol, pH 7.8 and the monovalent salt of interest at the indicated concentration) before the addition of the enzymes. The buffer was always pre-heated to the experiment temperature, i.e. 34°C if not indicated otherwise, before injecting into the flow cell. Preceding the measurement, we evaluated that the DNA tethers were relaxed. To this end, following data acquisition, DNA force-extension (varying the applied force from zero to high) and rotation-extension (twisting the tethers from −5 to 5 turn at 0.3 pN) tests were performed before holo addition in the flow chamber at +3 turns and 8 pN force (such force was used to prevent tethers twist change and magnetic bead sticking while flushing). The force was subsequently reduced to 0.3 pN and data acquisition was continued until the end of the experiment.

### Dwell times detection

To identify the points at which the DNA transitions between states (open and closed state, OS and CS, respectively), the so-called change points, we used an offline change point detection. With this analysis method, change points in the recorded data can be identified through finding the abrupt changes in the average magnetic bead position, even though neither the location nor the number of break points were known. For our purpose, we work with the change point detection algorithm as implemented in the Python package Ruptures (53). Change point detection is defined by a search method, a cost function and a constraint. The search method defines the algorithm used to analyze the time series. Here, as the true number of change points is unknown, we use the Bottom-Up algorithm. A dataset of length n is separated into *n*/2 segments and the pair segments with the lowest cost are merged until crossing a user defined penalty (see below). Bottom-Up has been shown to outperform the Binary Segmentation algorithm (54). As a cost function, we used a least absolute deviation to detect the changes in the median position of the magnetic bead. With an unknown number of change points, a constraint (penalty) is needed to balance out the goodness of fit parameter. The penalty was determined by manual inspection of change point detection quality for a particular data set. We use a penalty between 0.2 and 5 depending on the durations of the states. We embedded the Ruptures package into a GUI that is provided in our lab GitLab account (https://gitlab.com/DulinlabVU/change_point_analysis).

### Maximum likelihood fitting

The procedure is described in detail in (40). Briefly, the dwell times $\tau$ are described according to a probability-distribution function consisting either one or two exponentials,

$$\begin{equation*}f \left( t \right) = k{e}^{ - kt}\end{equation*}$$

and

$f ( t ) = {p}_ + {k}_ + {e}^{ - {k}_ + t} + {p}_ - {k}_ - {e}^{ - {k}_ - t}$ respectively. Here $k$, ${k}_ +$, ${k}_ -$ are characteristic rate constants, $ {p}_ +$ and $ {p}_ -$ are probabilities normalized such that $ {p}_ + + {p}_ - = 1$. The number of exponential fit to the data was determined using the Bayes Schwarz Information Criterion (BIC) (55). We calculate the maximum likelihood estimate of the parameters (MLE) (56) by minimizing the negative of the likelihood function

$$\begin{equation*}L = \mathop \sum \limits_{i = 1}^N {\rm{ln}}\left( {f\left( {{\tau }_i} \right)} \right) \end{equation*}$$

over the parameter set using minimization function with ‘L-BFGS-B’ algorithm in SciPy (SciPy.org). Here the ${\tau }_i$ are the experimentally measured dwell times and *N* is the number of collected dwell times ${\tau }_i$. The one standard deviation statistical error were extracted for each fitting parameter from 1000 bootstraps (57).

### Kinetic description of the open state formation

**Figure 2.**
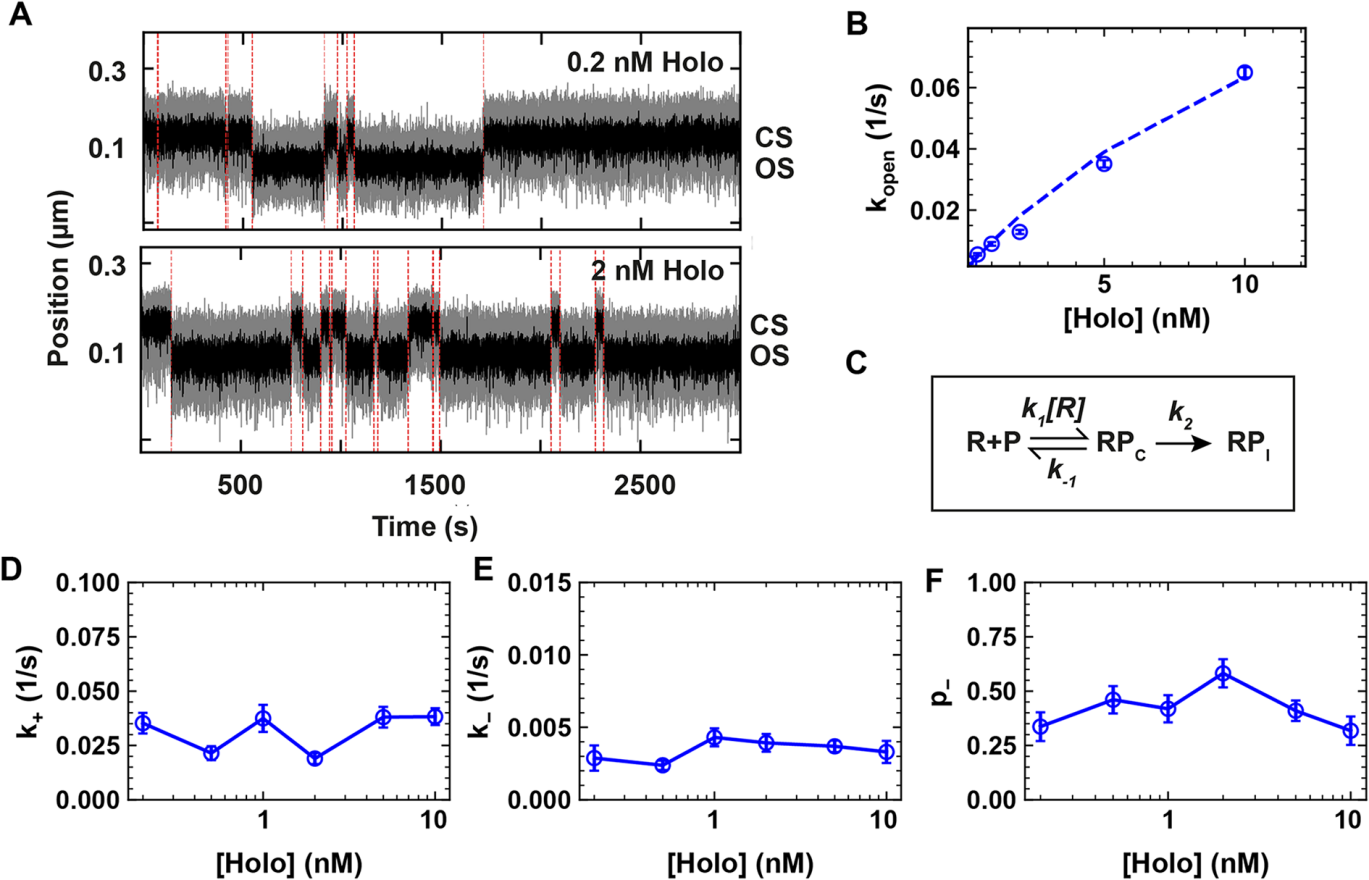
The CS dynamics is consistent with holo dissociation at the transition from OS to CS. (**A**) Traces showing the RP_O_ dynamics at 0.2 and 2 nM holo concentrations in 150 mM KAc. The dashed red lines indicate the transition between CS and OS. (**B**) CS exit rate constant ${k}_{open}$ as a function of holo concentration. The dashed line is a fit of the model described in (C) (Equation 1, Materials and Methods). (**C**) Kinetic model of the OS formation. RP_C_ and RP_I_ respectively indicates the holo-promoter closed complex and the holo-promoter intermediate open complex. ${k}_1$,$\ {k}_{ - 1}$, and ${k}_2$ are the rate constants for holo association, dissociation, and open to intermediate transitioning. (**D–F**) Double exponential fit parameters ${\rm{ }}{k}_ +$, ${k}_ -$ and ${p}_ -$ for the transitions from OS to CS as a function of holo concentration using the reaction buffer containing 150 mM KAc. Error bars are one standard deviation extracted from 1000 bootstraps in (C, D, E, F).

We consider the formation of the first state of the open complex, i.e. RP_I_, to be governed by the reaction scheme described in Figure 2C:

$$\begin{equation*}{\rm{R}} + {\rm{P}}\begin{array}{@{}*{1}{c}@{}} {{k}_1\left[ R \right]}\\ \rightleftharpoons \\ {{k}_{ - 1}} \end{array}{\rm{R}}{{\rm{P}}}_{\rm{C}}\mathop \to \limits^{{k}_2} \ {\rm{R}}{{\rm{P}}}_{\rm{I}}\end{equation*}$$

Previous studies have shown that holo binding equilibrates rapidly in comparison to the slow isomerization from RP_C_ to RP_I_ with the rate constant ${k}_2$ (8). We therefore write the observed rate constant of OS formation ${k}_{{\rm{open}}}$ as (58)

(1) $$\begin{equation*}{\rm{ }}{k}_{{\rm{open}}} = {P}_{{\rm{RPc}}} {k}_2, \quad {P}_{{\rm{R}}{{\rm{P}}}_{\rm{C}}} = \frac{{\left[ R \right]}}{{{K}_{\rm{D}} + \left[ R \right]}} , \quad {K}_D = {k}_{ - 1}/{k}_1 \end{equation*}$$

where ${P}_{{\rm{R}}{{\rm{P}}}_{\rm{C}}}$ is the fractional occupancy of the ${\rm{R}}{{\rm{P}}}_{\rm{C}}$ state at equilibrium, and we have defined the equilibrium dissociation constant ${K}_{\rm{D}}$. Equation (1) coincides with the ensemble description of Ruff and co-workers (8).

### Kinetic description of the OS to CS transition

The dissociation of the holo from the promoter is described by the kinetics of the transition from OS to CS. After we found the OS dwell time distribution was best fitted by a double exponential probability distribution function (pdf), we evaluated the first passage time distributions for several kinetic models to obtain the microscopic rate constants from the fit parameters $ {k}_ +$, $ {k}_ -$ and $ {p}_ -$ (Table 1). Models which did not result in a double exponential pdf for OS dwell times were discarded, as well as models that wouldn’t be rational based on literature. From this analysis, we concluded that the best model to describe the OS dwell time distribution corresponds to Model 4, Assumption 3 (Table 1):

$$\begin{equation*}R + P\mathop \leftarrow \limits^{{k}_5} R{P}_I\begin{array}{@{}*{1}{c}@{}} {{k}_3}\\ \rightleftharpoons \\ {{k}_{ - 3}} \end{array}R{P}_O\end{equation*}$$

**Table 1.** Comparison of models describing a double exponential distribution of the OS dwell times. pdf: probability distribution function. The left column presents four models. The red diamond indicates the holo kinetic state at which the OS dwell time starts. Model 1 describes a reversed kinetic pathway to dissociation from RP_C_. Model 2 consider two parallel dissociation pathways dominated by an intermediate RP_I_ state. Model 3 hypothesizes a dissociation from RP_O_, with two specific cases, i.e. the holo starts in OS from either RP_O_ or RP_I_, and eventually dissociates from RP_O_. Model 4 includes dissociation from RP_I_ and is solved in the context of three assumptions for which we have an analytical solution. The right column indicates the key parameter of the model, and the reason to either discard or keep a specific model

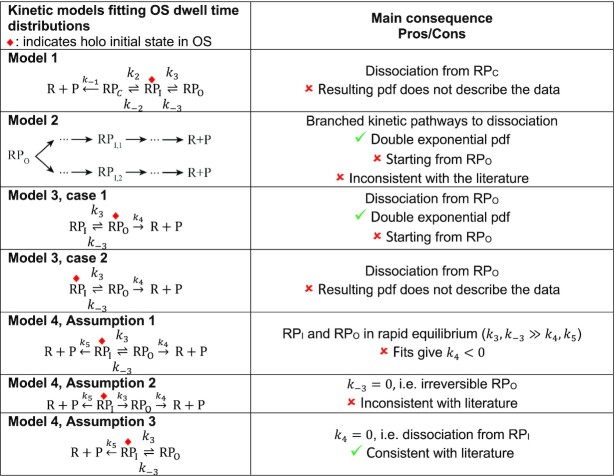

Under this assumption, we obtained a complete set of conversion relations for the fit parameters ${k}_ +$, $\ {k}_ -$ and $ {p}_ -$ to the microscopic rate constants ${k}_5$, ${k}_{ - 3}$, and ${k}_3$ (Supplemental information).

$$\begin{equation*} {k}_5 = {p}_ + {k}_ + + \left( {1 - {p}_ + } \right){{k}_ -} \end{equation*}$$

$$\begin{equation*}{\rm{ }}{k}_3 = \frac{{({k}_ + - {k}_5)({k}_5 - {k}_ - )}}{{{k}_5}}\ = \frac{{{p}_ + \left( {1 - {p}_ + } \right){{\left( {{k}_ + - {k}_ - } \right)}}^2}}{{{p}_ + {k}_ + + \left( {1 - {p}_ + } \right){k}_ - }}\ \end{equation*}$$

$$\begin{equation*}{\rm{ }}{k}_{ - 3} = \frac{{{k}_ + {k}_ - }}{{{k}_5}} = \frac{{{k}_ + {k}_ - }}{{{p}_ + {k}_ + + \left( {1 - {p}_ + } \right){k}_ - }} \end{equation*}$$

Note that since $0 \le {p}_ + \le 1$ and $ {k}_ +$, $ {k}_ - >0$ all the microscopic rate constants are positive, as should be.

### Error propagation from the double exponential fits to the kinetic rates

We propagated the errors of the MLE fitting parameters ($\Delta {k}_{{\rm{est}},{\rm{ }}i})$ to the rate constant of the kinetic model (Table 1) as $\Delta {k}_{{\rm{mic}},j}( {{x}_{{\rm{ }}i}} ) = \sqrt {\mathop \sum \limits_i {{( {\frac{{\partial {k}_{{\rm{mic}},j}}}{{\partial {x}_{{\rm{ }}i}}}\Delta {x}_{{\rm{ }}i}} )}}^2} {\rm{ }}$ where ${k}_{{\rm{mic}},j}$ is either of the derived microscopic rate constants ${k}_5$, ${k}_{ - 3}$, or ${k}_3$, and ${x}_{{\rm{ }}i}$ is either of the fit parameters $ {k}_ +$, $ {k}_ -$ or $ {p}_ -$. We use one standard deviation statistical error extracted from the bootstrap procedure.

### Theoretical description of the temperature dependence of the kinetic rate constants

For a two-state transition, the temperature dependence of the forward reaction is empirically described by the Arrhenius equation for the reaction rate constant ${k}_i$

(2) $$\begin{equation*}{\rm{ }}{k}_i = k_i^0 {e}^{ - \Delta {E}_i/{k}_{\rm{B}}T}\end{equation*}$$

where $k_i^0$ is the attempt rate, ${\rm{\Delta }}{E}_i$ is the activation energy, ${k}_{\rm{B}}$ is the Boltzmann constant, and $T$ is the temperature. Inserting Equation (2) in Equation (1), we can express the OS-formation rate constant as

(3) $$\begin{equation*}{\rm{ }}{k}_{{\rm{open}}} = \frac{{k_2^0{e}^{ - \Delta {E}_2/{k}_{\rm{B}}T}\left[ R \right]}}{{K_D^0{e}^{ - \left( {\Delta {E}_{ - 1} - \Delta {E}_1} \right)/{k}_{\rm{B}}T} + \left[ R \right]}} , \quad K_D^0 = k_{ - 1}^0/k_1^0 \end{equation*}$$

Equation (3) was fitted to the data in Figure 6B with a least-mean-squares fitting routine in Igor Pro 8 (WaveMetrics, Oregon, USA) to extract the activation energies $\Delta {E}_2$ and $ \Delta {E}_{diff} = \Delta {E}_{ - 1} - \Delta {E}_1$. The activation energies corresponding to the rate constants ${k}_5$, ${k}_3$ and ${k}_{ - 3}$ were extracted from a linear least-mean-squares fit (routine in Python 3) using the natural logarithm of Equation (2) to the temperature dependent values of the rate constants (Figure 6C–E).

## RESULTS

### High-throughput magnetic tweezers assay to study open complex dynamics for the bacterial RNA polymerase

We used a high-throughput magnetic tweezers assay to monitor the open complex dynamics for experiments up to several hours (Figure 1A, Supplementary Figure S1A). The magnetic bead was tethered to the top glass surface of the bottom coverslip of a flow cell by a ∼1.4 kb torsionally constrained DNA molecule, i.e. without nicks and with multiple attachment points between the handles and either the magnetic bead or the glass surface (Figure 1A) (Material and Methods). The flow cell surface was passivated using a lipid bilayer strategy, which significantly reduces the non-specific adhesion of magnetic beads and proteins to the flow cell surface (59–62). We provide a detailed protocol in the Materials and Methods section to establish such passivation strategy. The DNA sequence encodes the *lacCONS + 2* promoter for *E. coli* holo (Supplementary Figure S1B), which is a consensus versions of the *lacUV5* promoter (40), and has been extensively studied in ensemble (8) and single molecule experiments investigating bacterial transcription initiation (63).

The rotation of the magnets above the flow cell induces rotation of the magnetic bead, and concomitantly adds twist in the DNA tether. As the number of turns in the DNA molecule increases, torque also increases, up to the buckling transition at which the DNA starts forming plectonemes (50,64,65). Subsequent addition of turns to the DNA molecule is then converted into writhe, while keeping the torque constant, leading to a decrease of the DNA molecule end-to-end extension (50). In a rotation-extension experiment at ∼0.3 pN, the DNA extension is maximal at zero turn and decreases symmetrically when adding either positive or negative turns (Supplementary Figure S1C) (50). The linking number is the conserved sum of writhe and twist in a torsionally constrained nucleic acid molecule (66). The opening of the promoter (i.e. DNA unwinding or bubble formation) by the holo reduces the twist of the molecule, which is compensated by an increase in writhe for a positively supercoiled DNA molecule. This leads to a decrease of the end-to-end extension of the DNA molecule by $n \cdot \Delta z$, with $n$ being the number of open base pairs, and $\Delta z = \frac{{60}}{{10.5}} \ {\rm nm}/{\rm bp}$ (for a DNA helical pitch of ∼10.5 bp/turn) the rate at which the DNA molecule extension decreases per added twist (Figure 1A, Supplementary Figure S1C), as described by Strick and colleagues (43). Extracting the Allan deviation of the supercoiled tether (67,68), we measured the correlation time of the assay, i.e. its temporal resolution, to be ${t}_c = ( {45 \pm 8} ) \ {\rm ms}$ (Supplementary Figure S1D).

For a positively supercoiled DNA molecule and in the presence of holoenzyme, we clearly distinguished two main magnetic bead vertical positions, i.e. one indicating a DNA molecule without open complex that we coined the closed state (CS) and another one reporting a shorter DNA molecule end-to-end extension that signals a formed open complex and we coined the open state (OS) (Figure 1B) (43). The CS dwell time is the total time required for the holo to find, bind and open the promoter, while the OS dwell time is the total time the promoter is open until closing. As torque influences the open complex dynamics (43), we have only selected DNA molecules that were in a torsional state in the linear regime following the buckling transition in a rotation-extension experiment, where the torque is constant (Supplementary Figure S1C, E–H). We extracted the difference in extension between CS and OS for both positively and negatively supercoiled DNA, resulting in an average jump size of $( {73 \pm 8} )\ {\rm nm}{\rm{ }}$ and $( {42 \pm 6} )\ {\rm nm}$, respectively (Supplementary Figure S1I). Using these values, we determined a transcription bubble size of $( {10 \pm 1} )\ {\rm bp}$, and a DNA bended length of $( {16 \pm 5} )\ {\rm nm}$. These values are in good agreement with what Strick and colleagues previously measured, i.e. $( {13 \pm 1} )\ {\rm bp}$ and $( {15 \pm 5} )\ {\rm nm}$, respectively (43). Though we could measure indirectly the length of DNA bended, we could not observe directly this event, as it is very short-lived and beyond the assay temporal resolution (Supplementary Figure S1D). A visual inspection of a magnetic tweezers trace shows short-lived CS interrupted by either short or long-lived OS (Figure 1B). To quantify our observation, we use a Python-written custom graphic user interface (GUI), based on the change-point algorithm Ruptures (53) (Material and Methods, provided in https://gitlab.com/DulinlabVU/change_point_analysis), to automatically detect the transitions between OS and CS states in the magnetic tweezers traces. The measured CS and OS dwell times were subsequently assembled in distributions, which were fitted using a maximum likelihood estimation (MLE) procedure (56). The CS dwell time distributions were best fitted by a single exponential probability distribution function with a promoter opening rate constant ${k}_{open}$ (Figure 1C, Materials and Methods). In the presence of 150 mM potassium acetate (KAc), the OS dwell time distribution was best fitted by a double exponential probability distribution function with the fitting parameters ${k}_ +$, ${k}_ -$ and ${p}_ -$, i.e. the characteristic rate constants of the first and second exponential, respectively, and the probability of the second exponential (Figure 1D) (Materials and Methods) (40). This double exponential distribution, which suggests the existence of an intermediate open state (RP_I_), was not reported in previous magnetic tweezers study of the OS dynamics (43). The exit rates and probabilities extracted from the MLE fits performed on the dwell time distributions of all experiments are provided in Supplementary Table S1.

### A rapidly equilibrating binding followed by promoter opening describes open state formation

We first investigated the formation of open complex in 150 mM KAc at different holo concentrations. A direct observation of the traces shows a shorter CS dwell time when increasing holo concentration from 0.2 to 2 nM (Figures 1B and 2A). Extracting ${k}_{open}$ from the CS dwell time distributions (Supplementary Figure S2), we found ${k}_{open}$ to increase with holo concentration (Figure 2B) and be well described by a model where holo association to/dissociation from the promoter equilibrates quickly in comparison to the isomerization from RP_C_ to RP_I_ (Equation 1, Figure 2C), supporting previous magnetic tweezers observation by Strick and colleagues (43). From fitting Equation (1), we extracted the equilibrium dissociation constant ${K}_D = ( {17 \pm 2} ) \ {\rm nM}$ and the rate constant ${k}_2 = ( {0.16 \pm 0.02} ) \ {{\rm s}}^{ - 1}$ (Supplementary Table S3). The latter is only approximative, as we could not saturate the holo binding kinetics because of the insufficient temporal resolution of our assay (Supplementary Figure S1D), and of our inability to observe directly the RP_C_ state. The CS kinetics are consistent with a complete dissociation of the holo from the promoter following the transition from OS to CS, and the binding of a different holo preceding the next OS formation. This is further supported by the following three experiments. We first incubated the flow chamber with 0.5 nM holo in a reaction buffer to initiate and record CS–OS dynamics. The flow chamber was flushed about ∼1800 s after the start of the recording either with reaction buffer (Supplementary Figure S3A), reaction buffer containing ∼10 nM competing *lacCONS + 2* DNA promoter fragment (Supplementary Figure S3B) or reaction buffer containing 100 μg/ml heparin (Supplementary Figure S3C). All three experiments showed that after the flushing step the CS never converted back to the OS (Supplementary Figure S3). This finding confirms that the observed transition from OS to CS is in connection with the full release of the holo from the promoter. In the next section, we investigate through which kinetic state the holo dissociation takes place.

### Holo-promoter dissociations occurs from RP_I_

The OS dwell times kinetics were insensitive to the holo concentration, and strictly double exponentially distributed at 150 mM KAc (Figures 1D and 2D–F, Supplementary Figure S2), and not by a single exponential as previously reported (43). This indicates that the open complex is not formed by a single state, but suggest the existence of at least one intermediate preceding RP_O_ (8). Furthermore, the CS dwell times distributions are strictly single exponentially distributed (Figure 1C, Supplementary Figures S2, S5, S7–S9), indicating that CS ends from a single transition from RP_C_ to a single open state. Here, we could describe all the data in any experimental conditions with a single intermediate (RP_I_). The literature indicates that the holo very quickly isomerizes from RP_C_ to the first intermediate (rate constant ${k}_2$, Figure 2C) in comparison to the reverse reaction (slow, rate constant ${k}_{ - 2}$) and the forward reaction (8). The holo thus rapidly interconverts from RP_I_ to RP_O_. The OS dwell time distribution directly informs on the total duration of the open complex, i.e. from the promoter opening to its closing, which leads to holo dissociation. What is the kinetic pathway best describing the OS dwell times?

To answer this question, we discuss the merits of several kinetic models (Table 1). For each model, we determined the mathematical expressions of the rate constants describing the OS dwell time distribution. For the models describing a double exponential distribution, the absolute values of the rate constants were subsequently calculated using conversion expressions as a function of the MLE fit parameters (${k}_ +$, ${k}_ -$ and ${p}_ -$) (Materials and Methods, Supplementary Information). We first considered a model where the holo dissociates from walking back the kinetic pathway of RP_O_ formation to eventually dissociate from RP_C_ (Model 1, Table 1). Though this model is highly unlikely, given that ${k}_2$ is much larger than ${k}_ -$ and ${k}_ +$, we calculated the resulting probability distribution function (pdf) for such model (Supplementary Information). We found that the pdf would be the sum of a ‘peaked’ distribution (resembling a gamma distribution (69)) and an exponential decay, while we clearly observed a double exponential. The spatiotemporal resolution of the assay ($2 \cdot {t}_c \sim 90\ {\rm ms}$) may prevent us to capture the rising part of the peak distribution for very short-lived RP_C_ state, i.e. ${k}_{ - 1} \gg {k}_{ - 2},\ {k}_2$ (Table 1). In such a case, a double exponential description would be able to describe the OS dwell time distribution. However, this model would be inconsistent with previous ensemble studies of holo dissociation from the λP_R_ promoter (8,70). We therefore discarded this model, and we did not consider anymore dissociation from RP_C_ in the following models. Another possible model consistent with a double exponential function is Model 2 (Table 1), where the dissociation occurs through two parallel pathways, both being rate-limited by a different RP_I_ intermediate state. This model is unlikely, as Model 2 assumes that RP_I_ is an intermediate towards closing the open state, not opening, which is inconsistent with decades of literature (8), and, furthermore, suggests the existence of rapid irreversible intermediates preceding both RP_I_’s, for which we have no proof. Therefore, we discarded Model 2. In Model 3, we consider holo dissociation only from RP_O_, which has two possible cases, i.e. starting the OS from either RP_O_ (case 1) or RP_I_ (case 2). The former, i.e. Model 3 case 1 (Table 1), is described by a peaked dwell time distribution (Supplementary Information), which is not supported by our data that consistently showed a double exponential behavior (Supplementary Figure S2). We therefore discarded this model. In Model 3 case 2, the open complex starts from RP_I_ and dissociates from RP_O_ and is analytically described by a double exponential pdf (Model 3, case 2 in Table 1, Supplementary Figure S4). While this model is mathematically correct, it is conceptually irrational to have the open complex starting from RP_O_, and not in RP_I_ as described in the last 40 years literature (8), and we therefore discarded this model.

We introduced a fourth model, i.e. Model 4, where the holo may also dissociate from RP_I_ with rate constant ${k}_5$.

(4) $$\begin{equation*}{\rm{R}} + {\rm{P}}\mathop \leftarrow \limits^{{k}_5} {\rm{R}}{{\rm{P}}}_{\rm{I}}\begin{array}{@{}*{1}{c}@{}} {{k}_3}\\ \rightleftharpoons \\ {{k}_{ - 3}} \end{array}{\rm{R}}{{\rm{P}}}_{\rm{O}}\mathop \to \limits^{{k}_4} \ {\rm{R}} + {\rm{P}}\end{equation*}$$

A complete mapping of the fit parameters from the double exponential pdf, i.e. ${p}_ +$, ${k}_ +$, ${k}_ -$, to the kinetic rate constants of this model (${k}_3$, ${k}_{ - 3}$, ${k}_4$, ${k}_5$) cannot be made (Supplementary Information). We therefore proposed and test a set of simplifying assumptions that enable a complete mapping of the fit parameters to the underlying kinetic rate constants (Table 1, Supplementary Information). In Assumption 1, we assumed that RP_I_ and RP_O_ are in rapid equilibrium, i.e. ${k}_{ - 3}$ and ${k}_3$ are large in comparison to ${k}_5$ and ${k}_4$ (Table 1, Supplementary Information). Applying this model to our data resulted in negative values for ${k}_4$ (Supplementary Figure S4), which is unphysical and therefore rejected. In Assumption 2, we defined ${k}_{ - 3} = 0$, i.e. RP_O_ is an irreversible state leading to holo dissociation (Table 1, Supplementary Information). While this model results in a double exponential pdf and did not produce unphysical values for the rate constants (Supplementary Figure S4), it implies that the only way to rescue the holo from RP_O_ is dissociation, which is in contradiction with previous studies showing that such state should be reversible without dissociation (40,44). Finally, in Assumption 3, we defined ${k}_4 = 0$, i.e. the holo may enter RP_O_, but must return to RP_I_ to dissociate (Table 1, Supplementary Information). This model describes the OS dwell times with a double exponential pdf, without unphysical values for the rate constants and assumptions with the kinetic state lifetime (Supplementary Figure S4). In the following part of the study, we represented only the rate constants from Model 4 Assumption 3 (Table 1), which are provided in Supplementary Table S2. We subsequently investigate how the open-complex dynamics is affected by the nature and the concentration of monovalent salts, and the temperature in the context of this model.

### Anions affect open complex dynamics and cations affect DNA twist

**Figure 3.**
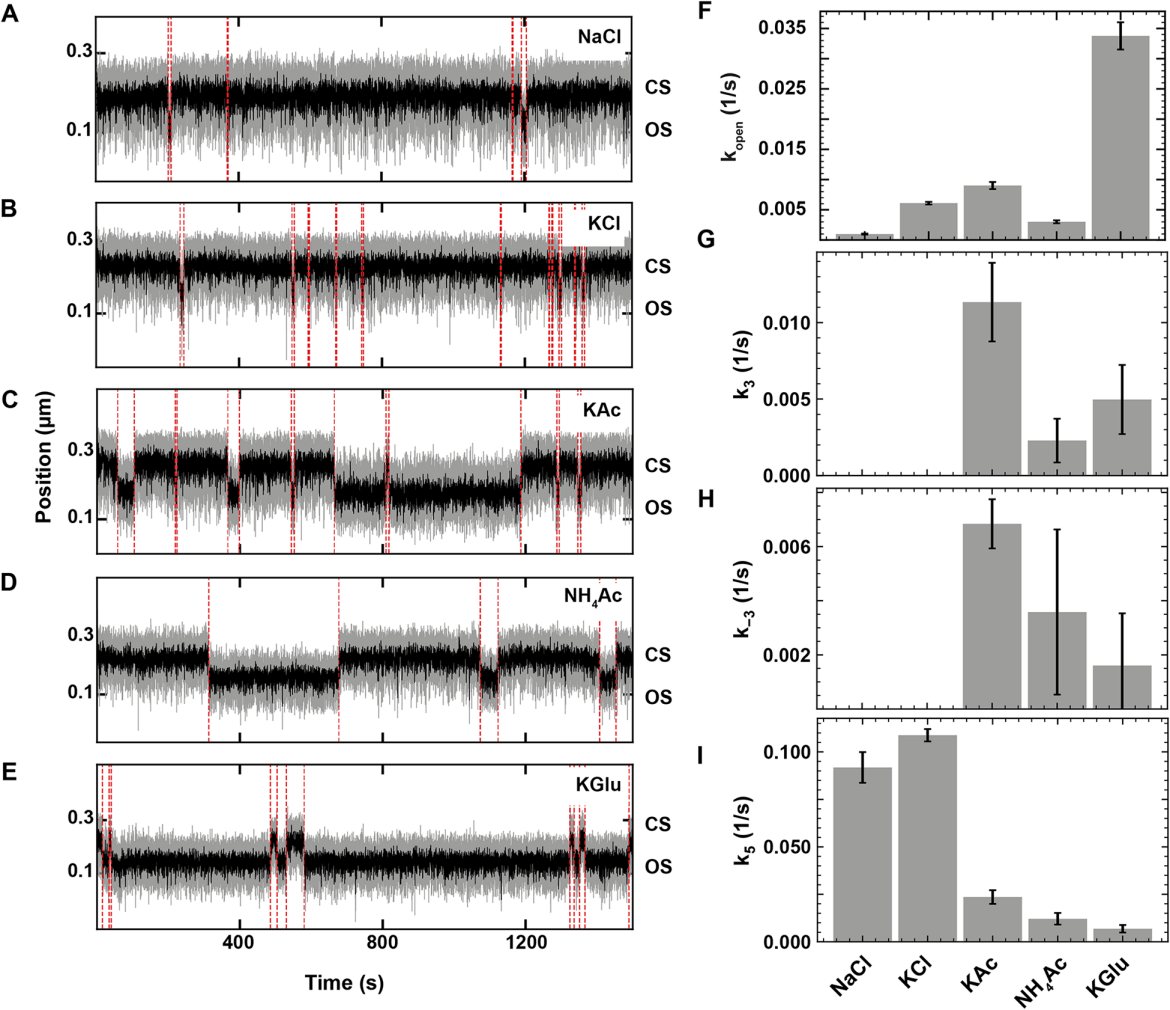
Monovalent salts affect bacterial holo open complex dynamics. (**A–E**) Holo open complex dynamics was observed at 34°C in the presence of 150 mM of the indicated monovalent salt. 10 nM holo was used in KCl and NaCl (A, B), and 1 nM holo was used in KAc, NH_4_Ac and KGlu (C–E). The red dashed lines indicate the transitions between OS and CS captured by the change-point analysis. (**F–I**) Dependence of ${k}_{open}$, ${k}_3$, ${k}_{ - 3}$ and ${k}_5$ on the monovalent salt type (Model 4, Assumption 3 in Table 1). The second exponential was absent from the dwell time distributions when using either NaCl or KCl, and we therefore extracted only ${k}_5$ for these conditions. Error bars in (F–I) are the propagated errors from the one standard deviation error extracted from 1000 bootstraps (Materials and Methods).

We first investigated how the identity of the monovalent ion affects the open complex dynamics. Previous ensemble studies showed that the DNA twist depends on the nature and the concentration of the monovalent cation (71). By affecting the DNA twist, the cation nature may impact bacterial transcription initiation kinetics (72), as the open complex dynamics is sensitive to torque (43) (Supplementary Figure S1C, E–H). We chose to compare potassium and sodium, the most common cations used in transcription studies, and ammonium, as it was shown to affect very strongly DNA twist (71). In addition to the data at 150 mM potassium acetate (KAc), we investigated open complex dynamics in the presence of 150 mM of either ammonium acetate (NH_4_Ac), potassium glutamate (KGlu), sodium chloride (NaCl) or potassium chloride (KCl) (Materials and Methods). We chose glutamate and chloride as anions because they are the most commonly used anions for in vitro transcription studies, and include acetate to complete the Hofmeister series ranking for the Coulombic interactions screening chloride > acetate > glutamate. We used 1 nM holo for all experiments except with chloride anion, where the holo concentration was increased to 10 nM to compensate for the stronger screening of electrostatic interactions by chloride that dramatically increase the CS lifetime (Figure 3). A direct observation of the activity traces shows very long CS and very short OS dwell times in the presence of NaCl and KCl, while the opposite trend is apparent when KGlu was used. KAc and NH_4_Ac presence induced an intermediate response, i.e. CS and OS having equal durations (Figure 3A–E). Analyzing the dwell time distributions showed that the CS dwell times are mono-exponentially distributed in all salts (Supplementary Figure S5), and ${k}_{open}$ is more than three-fold larger in KGlu than in other salts (Figure 3F), indicating that KGlu strongly favors the open complex formation. Furthermore, we found that the OS dwell times were very short and mono-exponentially distributed in the presence of 150 mM chloride anion, while they were double-exponentially distributed in the presence of the other anions (Figure 3G, Supplementary Figure S5). This result suggests that the complex never reaches the stable RP_O_ in the presence of high chloride concentration and can only populate the less stable RP_I_, by which the holo dissociates from the promoter (Model 4 Assumption 3, Table 1, Supplementary Information). In the presence of 150 mM KAc, NH_4_Ac and KGlu, the RP_I_ state is sufficiently stable (Figure 3I) for the holo to visit the RP_O_ state (second exponential appearing again in the OS dwell time distributions, Supplementary Figure S5). We could also estimate the isomerization rate constants to (${k}_3$) and away from (${k}_{ - 3}$) the RP_O_ state (Figure 3HI). The values indicate that the RP_O_ is most stable, i.e. ${k}_{ - 3}$ the smallest, in KGlu whereas KAc imposes rapid dynamics between the RP_I_ and the RP_O_ states.

Could the effect we observed here resulted from a change in the DNA twist due to the change of monovalent salt? Magnetic tweezers are a well-suited technique to characterize DNA twist variation, and we therefore investigated how the DNA twist (Δtwist) varied when changing the monovalent salt from NaCl to either KCl, KGlu, KAc or NH_4_Ac. Specifically, we performed extension-rotation experiments on a 20.6 kb coilable DNA tether (Supplementary Figure S6, Materials and Methods). We observed that changing the cation (sodium to potassium) in Tris-EDTA buffer induced a positive increase in twist by $( {135 \pm 7} )^\circ /{\rm kb}$; this cation effect was the same using either chloride, acetate or glutamate as the anion (Supplementary Figure S6A, B). Consistently with ensemble work (71), NH_4_Ac induces even larger increase in helical twist, i.e. $( {331 \pm 7} )^\circ /{\rm kb}$, in comparison to NaCl. When performing the same experiments in the holo reaction buffer, which contains 5 mM MgCl_2_, we observed a similar trend, though the effect is nearly two-fold smaller (Supplementary Figure S6B). Our data confirms that the cation affects the DNA helical twist and the strength of this effect follows the order Na^+^ < K^+^ < NH_4_^+^ (71,73). In contrast, our data did not show that the anion nature affects the DNA twist. We hypothesized that an increase in DNA helical twist would lead to a shorter-lived and less populated OS, as previously suggested (72). However, the observed difference in open complex dynamics (Figure 3G–I) is not consistent with the cation ranking for the helical twist (Supplementary Figure S6AB). For example, ${k}_{open}$ is 6-fold larger with KCl than with NaCl, though one would expect the opposite given the helical twist ranking effect, but 3-fold smaller for NH_4_Ac than for KAc. Overall, the anion nature has a much more significant impact on open complex dynamics than the monovalent cation, and we therefore performed the following experiments using only the physiological K^+^ cation.

### Physiological concentration of glutamate favors open complex formation and stability

**Figure 4.**
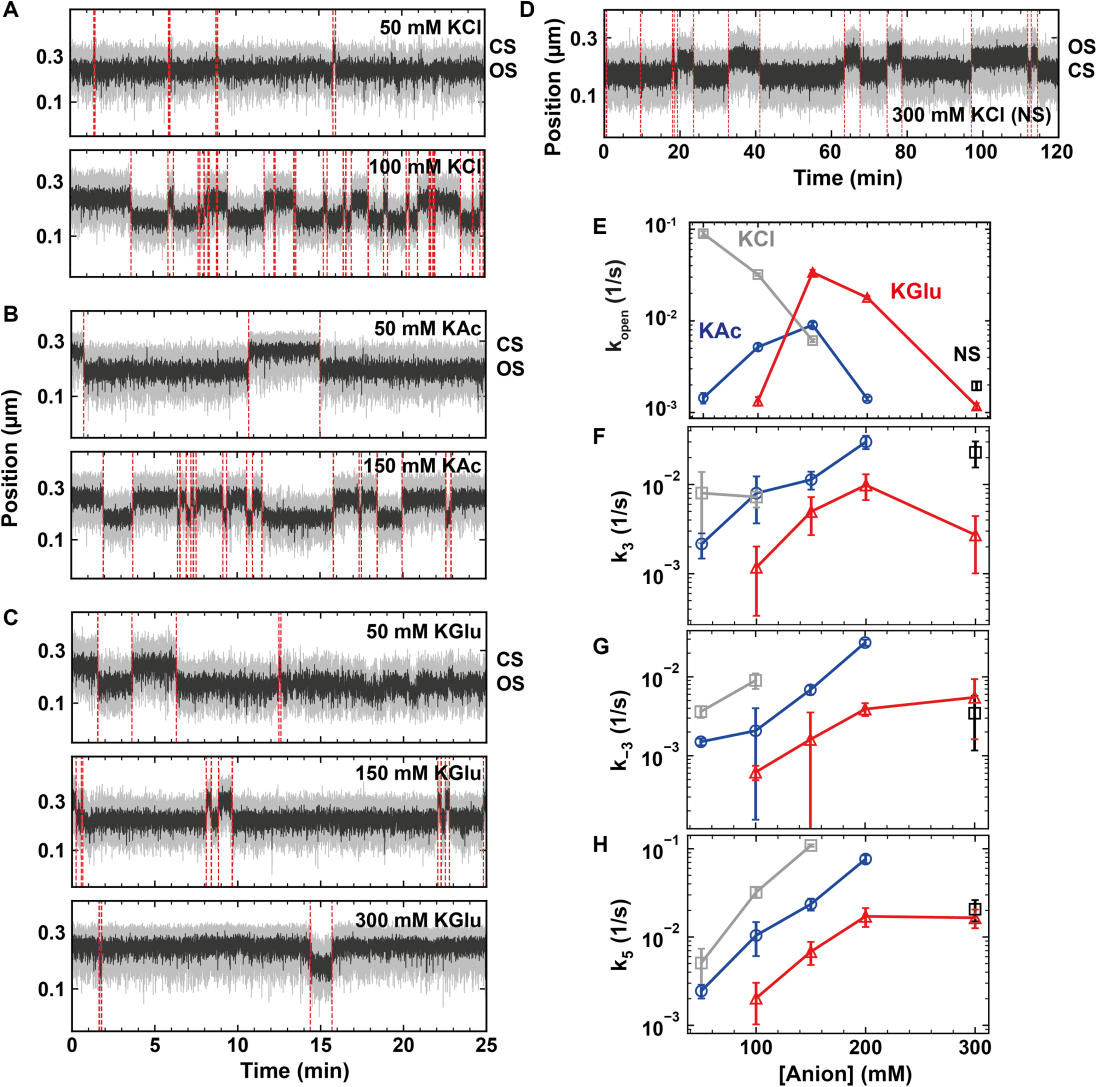
The anion type and concentration affect the holo open complex dynamics. (**A–C**) Traces of holo open complex dynamics using reaction buffers containing different anions at the indicated concentrations at 34°C. We used 10 nM holo in KCl, and 1 nM holo in KAc and KGlu. (**D**) Traces of holo open complex dynamics using 5 nM holo, 300 mM KCl and negatively supercoiled (NS) DNA. The red dashed lines indicate the transitions between OS and CS captured by the change-point analysis. (**E–H**) Monovalent salt concentration dependence of ${\rm{ }}{k}_{open}, {k}_3$, ${k}_{ - 3}$ and ${k}_5$ for KCl (grey), KAc (blue), KGlu (red), and KCl negatively supercoiled (NS) DNA (black) for activity traces acquired as in (A–D). The solid lines connect the markers and are not fits. Error bars in (E–H) are the propagated errors from the one standard deviation error extracted from 1000 bootstraps (Materials and Methods)

We next investigated how the changing concentration of chloride, acetate and glutamate affects the observed open complex dynamics at constant holo concentration. We chose these anions for specific reasons: glutamate is the most physiological anion; chloride is often used in *in vitro* bacterial transcription studies (42,43,74,75); acetate ranks between chloride and glutamate in the Hofmeister series (76) and is therefore interesting to investigate how Coulombic versus non-Coulombic interactions impact open complex formation. We varied the KCl concentration from 50 to 150 mM while using 10 nM holo in the reaction buffer. The activity traces showed shorter OS and longer CS as the KCl concentration increased (Figure 4A). Indeed, ${k}_{open}$ decreased steadily with KCl concentration ($S{k}_{open} = - 2.3$), indicating a loss in holo affinity with the promoter (Figure 4E, Supplementary Figure S7A–D, Table S4). Surprisingly, we found that the OS dwell times distributions were well described by a double-exponential pdf for KCl concentration up to 100 mM. Specifically, we found that the second exponential, and therefore the RP_O_ state, was completely depopulated for KCl concentration above 100 mM (Figure 4F–G, Supplementary Figure S7A–D), followed by a lower stability of RP_I_ at increasing KCl concentrations, e.g. ${\rm{ }}{k}_5$ increased ∼20-times ( $S{k}_5 = 2.8$) when increasing KCl concentration from 50 to 150 mM (Figure 4H, Supplementary Figure S7D, Table S4).

In comparison to KCl, the holo-promoter interactions were affected in a very different way by KAc and KGlu, as ${k}_{open}$ values first maximized at ∼150 mM ( $S{k}_{open, KAc} = 1.7$, $S{k}_{open, KGlu} = 8$) to significantly decrease at higher salt concentrations ($S{k}_{open, KAc} = - 6.5$, $S{k}_{open, KGlu} = - 5$) (Figure 4B, C, E, Supplementary Figure S7B–D, Table S4). The stability of both RP_O_ and RP_I_ decreased with KAc concentration, i.e. ${\rm{ }}{k}_5$ and ${k}_{ - 3}$ increased by more than one order of magnitude ($S{k}_5 = 2.4$, $S{k}_{ - 3} = 2$), while the conversion rate constant from RP_I_ to RP_O_, i.e. ${k}_3$, surprisingly increases by more than 10-fold ($S{k}_3 = 1.8$) (Figure 4F-H, Supplementary Figure S7B–D, Table S4). While glutamate shows a destabilizing effect on the open complex similarly to acetate, the glutamate effect saturates above 200 mM concentration. We could not measure RP_O_ dynamics at KCl and KAc concentrations larger than 150 mM and 200 mM, respectively, as OS were hardly detected. RP_O_ stability in the presence of acetate is intermediate between chloride and glutamate. Interestingly, 50 mM KCl showed a faster open complex formation than 150 mM KGlu, while maintaining a comparable stability of the OS (Figure 4E–H). However, RP_O_ state is able to resist higher concentrations of the physiological anion glutamate than chloride, supporting the hypothesis that glutamate is an open complex stabilizer (77,78).

As DNA in bacterial cells is naturally negatively supercoiled, we have investigated whether the dynamic observed with positively supercoiled DNA is conserved (Supplementary Figure SS1J). Because holo forms a very stable open complex on *lacCONS* promoter when negatively supercoiled (43), we needed to significantly increase the monovalent salt concentration to investigate the pathway towards dissociation. The open complex formed rapidly at 200 mM KCl with negatively supercoiled DNA, but showed almost no dynamics (Supplementary Figure S8A). We observed a similar behavior when replacing KCl with 300 mM KGlu (Supplementary Figure S8B). Increasing KCl concentration to 300 mM was necessary to monitor open complex dissociation dynamics (Figure 4D). By extracting the dwell times of OS and CS, and fitting the respective distribution with MLE, we show that CS dwell time distribution is well described by a single exponential, while OS dwell time distribution is double exponentially distributed (Supplementary Figure S8CD). Interestingly, the open complex dynamics for negatively supercoiled promoter in 300 mM KCl is similar to the ones for positively supercoiled promoter in 300 mM KGlu (Figure 4E–H). The open complex formation is strongly impaired by the high KCl concentration, similarly to the impact of 300 mM KAc and KGlu when using positively supercoiled DNA (Figure 4E). The dissociation dynamics is also rather similar to the one observed at 300 mM KGlu conditions on positively supercoiled DNA, with the exception that the transition from RP_I_ to RP_O_, i.e. ${k}_3$, increased almost 10-fold (Figure 4FGH). Our results show that the dissociation mechanism is conserved, independently of the supercoiling sign.

**Figure 5.**
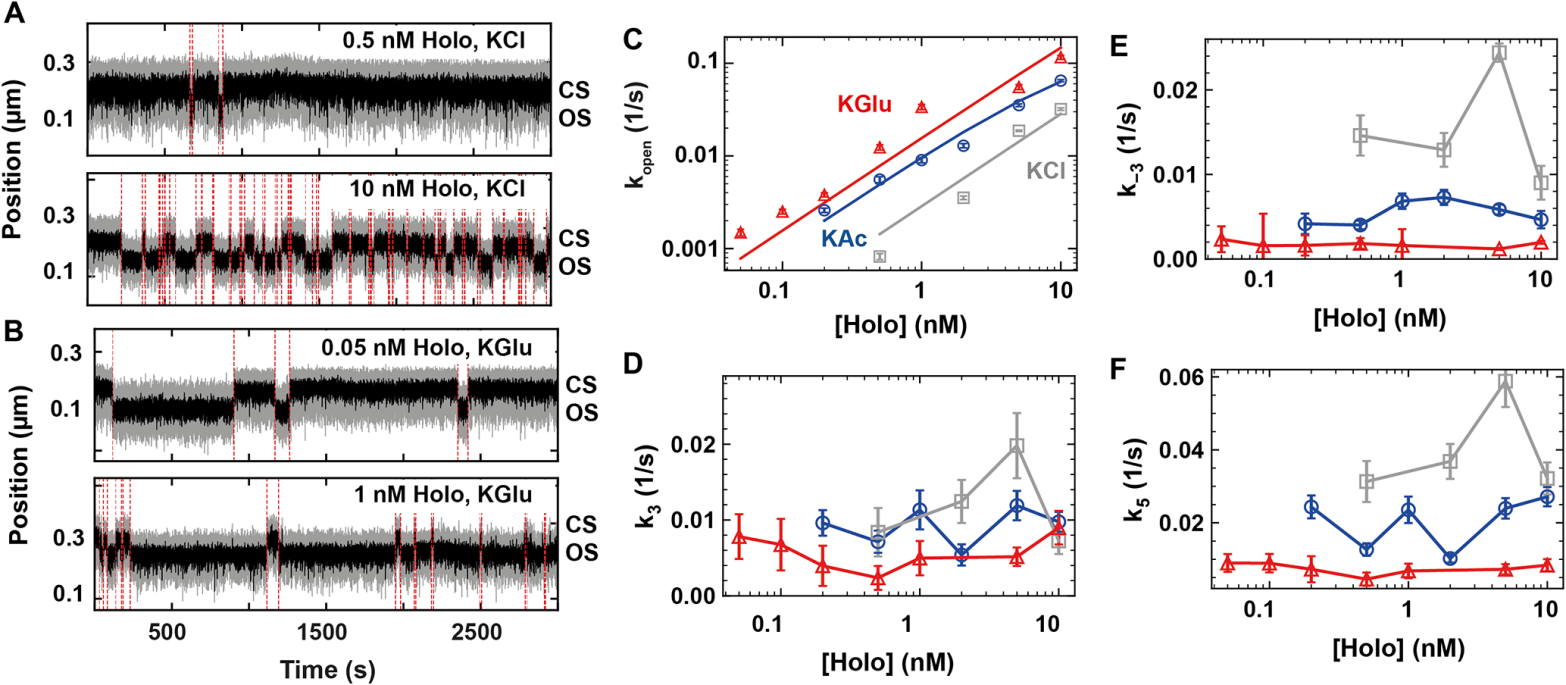
The holo releases the promoter upon transcription bubble closing. (**A, B**) Magnetic tweezers activity traces showing open complex dynamics in either (A) 100 mM KCl, or (B) 150 mM KGlu at 34°C using the indicated holo concentration. The red dashed lines indicate the transitions between OS and CS captured by the change-point analysis. (**C–F**) Holo concentration dependence of ${k}_{open}$, ${k}_3$, ${k}_{ - 3}$ and ${k}_5$ in either 100 mM KCl, 150 mM KGlu or 150 mM KAc. Color code as in (C). The solid lines in (C) are fits to Equation (1). The solid lines in (D–F) connect the markers and are not fits. Error bars in (C–F) are the propagated errors from the one standard deviation error extracted from 1000 bootstraps (Materials and Methods)

Having found the optimum concentration of chloride, acetate and glutamate, we investigated the open complex formation and stability as a function of holo concentration (Figure 5). We performed these experiments in either 100 mM KCl or 150 mM KGlu, and represent these data next to the 150 mM KAc data presented in Figure 2. The CS dwell times visually shortened in magnetic tweezers traces with increasing holo concentration (Figure 5A, B). Extracting ${k}_{open}$ from the CS dwell time distributions (Supplementary Figure S9) and representing it as a function of holo concentration for KCl and KGlu, we observed a similar trend as for KAc and the data were well fitted by Equation (1), supporting that the holo rapidly dissociates from the promoter upon closing and is not recycled for the subsequent OS (Figure 5C). For KGlu, we extracted an equilibrium dissociation constant ${K}_D = ( {7 \pm 1} )\ nM$ and ${\rm{ }}{k}_2 = ( {17 \pm 0.02} ) \ {s}^{ - 1}$ (the latter value is only indicative as we could not reach saturation in holo concentration), while for KCl the fit was poor and the fit parameters were therefore unrealistic (Supplementary Table S3). As for KAc, the dynamics of the OS, i.e. ${k}_{ - 3}$, ${k}_3$ and ${k}_5$, is mostly unaffected by holo concentration in both KCl and KGlu (Figure 5D–F). We noted the values at 5 nM holo in KCl are ∼2-fold higher than at other holo concentrations. We do not have an explanation for this behavior.

### Open complex formation energy landscape probed by temperature-controlled magnetic tweezers

**Figure 6.**
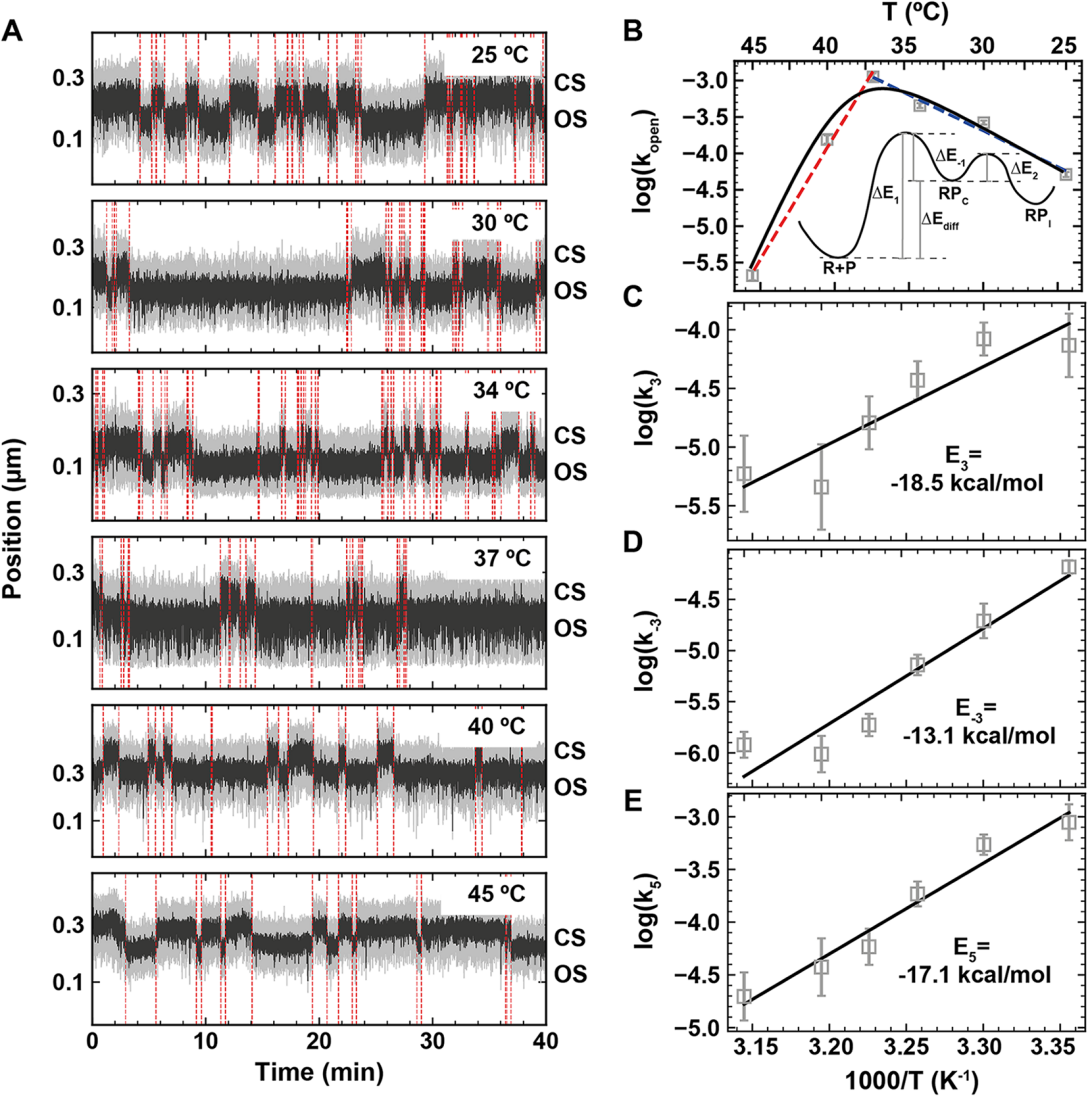
Effect of temperature on the holo open complex dynamics. All experiments were performed in 150 mM KAc and 5 nM holo. (**A**) Magnetic tweezers activity traces showing open complex dynamics at the temperature indicated in the plots. The red dashed lines indicate the transitions between OS and CS captured by the change-point analysis. (**B**) Arrhenius plot of ${k}_{open}$ where the solid line is a fit to the data using Equation (3). Blue and red dashed lines in (B) are fits using Equation (2) restricted to ${k}_{open}$ extracted for temperatures either below or above 37°C, respectively. The energy landscape of the open state formation is represented within the plot. (**C–E**) Arrhenius plot of ${k}_3$, ${k}_{ - 3}$ and ${k}_5$. Solid lines are fit to the data using Equation (2). Error bars in B–E are the propagated errors from the one standard deviation error extracted from 1000 bootstraps (Materials and Methods)

Temperature dependence of the bacterial open complex formation enables the exploration of the energy landscape of the reaction (33,79,80). We have recently developed a temperature-controlled magnetic tweezers assay (52), and we applied it to investigate how temperature affects the kinetics of the open complex dynamics in real-time. Because KGlu induces extremely stable OS, we performed this study in 150 mM KAc and 5 nM holo to maximize the statistics of the open complex dynamics as a function of temperature (Figure 6A). Nonetheless, we expect our results to be conserved for KGlu, as the open complex dynamics shows a similar trend in either acetate or glutamate (Figure 4).

We showed here that the CS dwell times distribution was strictly mono-exponential described by an exit rate constant ${k}_{open}$ that is strongly holo concentration dependent (Supplementary Figure S10). Therefore, if the holo was degraded/denatured during the course of the experiment (several hours) at the elevated temperature, we would have expected the CS dwell times distribution to not be accurately fitted by a mono-exponential, but a probability distribution function representative of the holo concentration decrease over time, i.e. a multi-exponential. The observed CS dwell time distributions at all temperatures indicate that the holo remained functional for the entire duration of the experiment. The MLE fits revealed that ${k}_{open}$ increased by ∼4-fold when temperature was increased from 25°C to 37°C, and subsequently decreased by ∼13-fold when the temperature further increased from 37°C to 45°C (Figure 6B). ${k}_{open}$ cannot be fitted by a simple Arrhenius equation, as the Arrhenius plot does not appear curvilinear (Figure 6B, Supplementary Figure S10) (81). This behavior is similar to what was previously described for the temperature dependence of fibrinopeptide release by thrombin (82), and support the existence of a closed intermediate, i.e. RP_C_, between R + P and RP_I_ states, though we cannot directly observe it during the open complex formation (Figures 2C and 5C). The transition with the lowest activation energy, i.e. from RP_C_ to RP_I_, dominates the reaction at low temperature, while the transition with the highest activation energy dominates at high temperature, i.e. holo dissociation from the promoter. To the best of our knowledge, such behavior was not described in the previous investigations of the temperature dependence of the open complex formation with strong promoters, likely because holo dissociation from RP_C_ never dominated (33,79,80). Using Equation (3) (Materials and Methods), we extracted the activation energy of the transition from RP_C_ to RP_I_, i.e. $\Delta {E}_2 = ( {22 \pm 6} ) \ {\rm kcal\cdot mol}^{ - 1}\ {\rm or}\ ( {92 \pm 25} )\ {\rm kJ\cdot mol}^{ - 1}$, and the energy difference between the unbound state R + P and RP_C_, i.e. $\Delta {E}_{diff} = \Delta {E}_{ - 1} - \Delta {E}_1 = ( {107 \pm 12} ) \ {\rm kcal\cdot mol}^{ - 1}\ {\rm{or}}\ ( {448 \pm 50} )\ {\rm kJ\cdot mol}^{ - 1}$ (Figure 6B). Our evaluation of $\Delta {E}_2$ is in agreement with previous estimation made using the *lacUV5* promoter (33,80).

From the OS dwell time distributions (Supplementary Figure S10), we extracted the temperature dependence of ${k}_3$, ${k}_{ - 3}$ and ${k}_5$. Their respective Arrhenius plot were well described by Equation (2), and we extracted the activation energies ${E}_3 = ( { - 18.5\ \pm 2.9} )\ \ {\rm kcal\cdot mol}^{ - 1}\ {\rm{or\ }}( { - 77 \pm 12} )\ {\rm kJ\cdot mol}^{ - 1}$, ${E}_{ - 3} = ( { - 13.1 \pm 3.1} ) \ {\rm kcal\cdot mol}^{ - 1}$${\rm{or}}\ ( { - 55 \pm 13} )\ {\rm kJ\cdot mol}^{ - 1}$ and ${E}_5 = ( { - 17.1 \pm 1.7} ) \ {\rm kcal\cdot mol}^{ - 1}\ {\rm{or}}\ ( { - 72 \pm 7} )\ {\rm kJ\cdot mol}^{ - 1}$, respectively (Figure 6C–E). Interestingly, all these activation energies are negative. An elementary reaction must have a positive activation energy. However, if the observed reaction occurs via an unknown and stable intermediate, e.g. an open complex intermediate in the present situation, the complex may be trapped in this intermediate while increasing the temperature, resulting in a product apparently more stable at higher temperature (83). Furthermore, such intermediates are not rate-limiting in the forward reaction, i.e. open complex formation, as we do not have any kinetic indication of their existence, though a recent cryoEM study has indicated several open intermediates (36). While for *lacUV5* and $\lambda$*P_R_*, negative $ {E}_{ - 3}$ and ${E}_5$ were already observed, we also note that ${E}_3$ is negative, indicating the existence of another intermediate between RP_I_ and RP_O_, as previously suggested for $\lambda$*P_R_* promoter, i.e. I_3_ (35,84).

## DISCUSSION

In the present study, we have investigated the bacterial open complex formation and dissociation on a consensus *lacUV5* (*lacCONS*) promoter using high-throughput magnetic tweezers. We have studied the impact of the nature and concentration of the monovalent salt, holo concentration and temperature on the kinetics of formation and dissociation. While some of these aspects have been investigated in ensemble studies with $\lambda$P_R_ and *lacUV5* promoters, such investigations have never been performed at the single molecule level. Furthermore, we show here that the choice of the monovalent salt may have dramatic consequences on the kinetics observed at the single molecule level. Seminal work by Strick and colleagues using magnetic tweezers showed that the holo specifically binds at the promoter to form an RP_C_ and transits directly towards a stable RP_O_, the lifetime of which varies as a function of the promoter sequence, the applied torque and the supercoiling sign (43). Interestingly, they reported no intermediate between RP_C_ and RP_O_, and a single dissociation rate constant, though ensemble studies already reported at least one intermediate (8). Furthermore, a recent biochemical study has characterized several closed-promoter intermediates preceding RP_I_, which originate from promoter bending and conformational rearrangement of the RNAP clamp to position the promoter towards opening (26), supporting recent cryoEM studies (25,36). A recent single-molecule FRET study has showed that the holo explores an RP_I_ state, either transiently or permanently, in addition to the fully open RP_O_ state (44). One of the differences between the magnetic tweezers and the single molecule FRET studies was the nature of the monovalent salt: the former used NaCl, while the latter used KGlu. Record and colleagues have shown that the physiologically relevant glutamate has a stabilizing effect on protein folding and RP_O_ formation over chloride, and specifically interacts with the holo to drive major conformational changes from RP_I_ (called I_2_ in $\lambda$P_R_ studies) to RP_O_ (78). These experiments were performed at rather high monovalent salt concentration, i.e. from 150 to 545 mM, and did not investigate lower, and more physiological, salt concentrations. We have filled this gap by investigating a range of monovalent salts from 50 to 300 mM. We show here that open complex dynamics are mainly affected by the type of the anions, i.e. Cl^–^, Glu^–^ and Ac^–^. In agreement with previous studies (77,85), our direct observation of RP_O_ formation using a torsionally constrained DNA molecule showed no effect of the monovalent cation, i.e. K^+^, Na^+^ and NH_4_^+^ (Figure 3G–I), despite the increase in the DNA helical twist induced by potassium and ammonium in comparison to sodium (Supplementary Figure S6). Though the rate constant for open complex formation was similar at 50 mM KCl and 150 mM KGlu, it decreased exponentially with KCl concentration (Figure 4E), until no activity was detected above 150 mM. This result suggests that increasing chloride concentration screens the holo Coulombic interactions with the promoter, and consequently decreases the equilibrium association constant ${K}_1$. KGlu and KAc impacts the open complex formation in two different regimes, up to ∼150 mM, KGlu increases attractive interactions between the promoter and the holo, to eventually screen these interactions by Coulombic effect at higher concentrations.

We studied the dissociation kinetics of the holo from *lacCONS* promoter to determine the reaction pathway between RP_O_ and RP_I_. Kinetic modeling clearly supports a description where RP_O_ is a stable but still slowly reversible state and holo dissociation occurs only via RP_I_ (Model 4 Assumption 3, Table 1). This is in agreement with ensemble studies inducing dissociation with high salt upshift (70). How does anion concentration affect open complex stability? The dissociation rate constant ${k}_5$, i.e. from RP_I_ to R + P, increased exponentially with glutamate and acetate concentration, and even more so for chloride, up to the point that the holo did not enter RP_O_ state at > 100 mM chloride. The strong dependence of RP_I_ dissociation on chloride could explain why a previous magnetic tweezers study reported only a single rate constant for RP_O_ dissociation, as if the entire OS population was in the RP_I_ state (43). Interestingly, a follow up study by the same group on initial transcription and promoter escape showed RNA synthesis activity in the same ionic condition, suggesting that RP_I_ may be the catalytically competent state (74), as recently reported by Record *et al.* (84), and further supported by a recent structural work from Darst and colleagues (86). What could be the utility of such long-lived, catalytically incompetent RP_O_? A recent magnetic tweezers study reported a long-lived backtrack state of the initially transcribing complex (ITC) using positively supercoiled DNA (42). This state was described by a double exponential, with exit rates of ∼0.003 and ∼0.0002 s^–1^, respectively (42). We show in another study that such state is catalytically incompetent, its escape is sensitive to the nucleotide concentration, and may serve as a mean to trap the holo at the promoter to regulate gene expression as a function of NTP concentration (40). We propose here that the holo is similarly capable to enter a long-lived inactive open state in the absence of RNA and NTPs, forming the so-called RP_O_. Recovery from the inactive state is slow on the strong consensus promoter (∼0.05 s^–1^ in 100 mM KCl) but can be speculated to be faster on promoters with less ideal –10 or discriminator sequences, or when additional transcription factors bind and modulate the RP_O_.

We could not observe a difference in transcription bubble size between RP_I_ and RP_O_, within the spatiotemporal resolution of our assay, indicating that this transition does not lead to an intermediate state with a different bubble size long-lived enough to be detected. It may rather originate from the rearrangement of the melted DNA strands inside the holo, as previously suggested (77), and/or involves conformation change in the holo. However, we cannot exclude the existence of rapid bubble expansion or reduction indicative of other intermediate state(s). These rearrangements tighten the holo–promoter interactions effectively preventing the dissociation from RP_O_ state. In the RP_I_ state, in contrast, these interactions remain weak enough to allow their stochastic disruption and thus direct dissociation from the RP_I_ state without full reversal to the state preceding the open complex, i.e. RP_C_. Furthermore, acetate shows an intermediate effect on the kinetics of ${k}_3$, ${k}_{ - 3}$ and ${k}_5$, following the Hofmeister series ranking of acetate between chloride and glutamate, and being also consistent with the idea that glutamate favors open complex formation via non-Coulombic interactions (77).

By investigating the temperature dependence of the RP_O_ dynamics, we extracted the activation energies of the transitions between the different states. We showed that, in our experimental conditions, i.e. positively supercoiled DNA and 150 mM KAc, a simple Arrhenius equation could not describe the temperature dependent open complex formation kinetics (Figure 6B). We thus proposed a model, in which the transition from RP_C_ to RP_I_ (${k}_2$ in Figure 2C) dominates the reaction at low temperature, while RP_C_ to holo dissociation (${k}_{ - 1}$ in Figure 2C) dominates the reaction at high temperature. To estimate how these changes in the rate-limiting steps affect the formation and relative abundance of different RNAP–promoter complexes, we simulated, using the experimental rate constants from Supplementary Table S2, the time and temperature dependent evolution of nascent RNAP–promoter population (Supplementary Figure S11). In each temperature, RP_I_ concentration peaked ∼60–120 s before RP_O_ begins to accumulate significantly. The equilibrium binding of the holo to the promoter was most efficient in the physiologically optimal temperature as indicated by the fact that the relative free promoter concentration was 0.25 at 37°C, increasing to 0.70 or 0.58 towards the low (25°C) and high (45°C) end of the studied temperature range, respectively. Similar trend was observed for the RP_O_/RP_I_ ratio, i.e. 1.05 at 25°C, 2.42 at 37°C and 1.85 at 45°C. Overall, the simulation demonstrate that temperature modulates significantly both the overall holo binding to the promoter and the equilibrium levels between RP_I_ and RP_O_. We also note that there appears a time window (at about 10–60 s in Supplementary Figure S11), where RP_I_ is the dominant complex with insignificant amount of RP_O_ formed. It is therefore plausible to speculate that RP_I_ can be kinetically selected as the predominant transcription initiation complex, i.e. RP_I_ would initiate RNA synthesis before isomerization to RP_O_ had time to occur. However, it should be noted that the simulation was carried using a simple three species mechanism (Supplementary Figure S11). As previously observed (33,84), the activation energies of the transition described by the rate constants ${k}_3$, ${k}_{ - 3}$ and ${k}_5$ were in fact negative, which indicates that these rates do not actually report on elemental reactions (83). This finding supports the existence of intermediate states between RP_O_ and RP_I_ (and vice versa) and between RP_I_ and R + P, which are not kinetically significant in the net forward reaction of RP_O_ formation.

A recent cryoEM study from Darst and colleagues has reported seven intermediates during the formation of *E. coli* holo open promoter complex on *rpsT P2* promoter in combination with a transcription factor inhibitor trapping the open complex in different open complex state (36). Previous ensemble biochemical studies have reported on three distinct open intermediates on $\lambda$P_R_, one rapidly equilibrating with the most stable RP_O_ state, and therefore challenging to observe (8,33). Our recently published single molecule FRET investigation reported several open complex states with kinetic rate constants in the sub-second range (87), i.e. beyond the temporal resolution of our magnetic tweezers assay. However, the negative activation energies of the transitions towards dissociation we present here strongly support their existence. Could the seven states observed by cryoEM be a combination of the transient open states reported using single molecule FRET and the slower ones we report here? To answer this question, future studies will need to directly observe such intermediates using high resolution magnetic tweezers combined with single molecule FRET to reveal the complete cycle of open complex formation and dissociation. Furthermore, other promoter sequences may be more appropriate to reveal such intermediates, as open complex formation kinetics varies widely for different promoters (43).

Our study expands the understanding on how monovalent salts and temperature affect protein-nucleic acids interactions, and will therefore be of use to single molecule biophysicists. We provide the community a detailed protocol to establish a robust lipid bilayer passivation for single molecule assays, as well as a complete pipeline for data analysis using a custom Python routine. Furthermore, our single molecule study of bacterial RNA polymerase open complex dynamics corroborates the existence of open-state intermediates, and further expands our understanding of the interactions leading to a stable holo open complex.

## DATA AVAILABILITY

The data of this study are available from the lead authors upon reasonable request.

## Supplementary Material

gkac560_Supplemental_Files

## References

[1] Burgess R.R. , Travers A.A., Dunn J.J., Bautz E.K. Factor stimulating transcription by RNA polymerase. Nature. 1969; 221:43–46. 4882047 10.1038/221043a0

[2] Sklar V.E. , Schwartz L.B., Roeder R.G. Distinct molecular structures of nuclear class I, II, and III DNA-dependent RNA polymerases. Proc. Natl. Acad. Sci. U.S.A. 1975; 72:348–352. 1054509 10.1073/pnas.72.1.348 PMC432302

[3] Zhang G. , Campbell E.A., Minakhin L., Richter C., Severinov K., Darst S.A. Crystal structure of thermus aquaticus core RNA polymerase at 3.3 a resolution. Cell. 1999; 98:811–824. 10499798 10.1016/s0092-8674(00)81515-9

[4] Cramer P. , Bushnell D.A., Fu J., Gnatt A.L., Maier-Davis B., Thompson N.E., Burgess R.R., Edwards A.M., David P.R., Kornberg R.D. Architecture of RNA polymerase II and implications for the transcription mechanism. Science (New York, N.Y). 2000; 288:640–649. 10.1126/science.288.5466.640 10784442

[5] Hirata A. , Klein B.J., Murakami K.S. The X-ray crystal structure of RNA polymerase from archaea. Nature. 2008; 451:851–854. 18235446 10.1038/nature06530 PMC2805805

[6] Mosaei H. , Harbottle J. Mechanisms of antibiotics inhibiting bacterial RNA polymerase. Biochem. Soc. Trans. 2019; 47:339–350. 30647141 10.1042/BST20180499

[7] Feklistov A. , Sharon B.D., Darst S.A., Gross C.A. Bacterial sigma factors: a historical, structural, and genomic perspective. Annu. Rev. Microbiol. 2014; 68:357–376. 25002089 10.1146/annurev-micro-092412-155737

[8] Ruff E.F. , Record M.T. Jr, Artsimovitch I. Initial events in bacterial transcription initiation. Biomol. 2015; 5:1035–1062. 10.3390/biom5021035 PMC4496709 26023916

[9] Mazumder A. , Kapanidis A.N. Recent advances in understanding sigma70-dependent transcription initiation mechanisms. J. Mol. Biol. 2019; 431:3947–3959. 31082441 10.1016/j.jmb.2019.04.046 PMC7057261

[10] Singer P. , Wu C.W. Promoter search by escherichia coli RNA polymerase on a circular DNA template. The J. Biol. Chem. 1987; 262:14178–14189. 3308887

[11] Ricchetti M. , Metzger W., Heumann H. One-dimensional diffusion of *Escherichia coli* DNA-dependent RNA polymerase: a mechanism to facilitate promoter location. Proc. Natl. Acad. Sci. U.S.A. 1988; 85:4610–4614. 3290898 10.1073/pnas.85.13.4610 PMC280484

[12] Kabata H. , Kurosawa O., Arai I., Washizu M., Margarson S.A., Glass R.E., Shimamoto N. Visualization of single molecules of RNA polymerase sliding along DNA. Science (New York, N.Y). 1993; 262:1561–1563. 10.1126/science.8248804 8248804

[13] Guthold M. , Zhu X., Rivetti C., Yang G., Thomson N.H., Kasas S., Hansma H.G., Smith B., Hansma P.K., Bustamante C. Direct observation of one-dimensional diffusion and transcription by *Escherichia coli* RNA polymerase. Biophys. J. 1999; 77:2284–2294. 10512846 10.1016/S0006-3495(99)77067-0 PMC1300507

[14] Harada Y. , Funatsu T., Murakami K., Nonoyama Y., Ishihama A., Yanagida T. Single-molecule imaging of RNA polymerase-DNA interactions in real time. Biophys. J. 1999; 76:709–715. 9929475 10.1016/S0006-3495(99)77237-1 PMC1300075

[15] Heller I. , Marchetti M., Mazumder A., Chakraborty A., Malinowska A.M., Roos W.H., Ebright R.H., Peterman E.J., Wuite G.J. One-dimensional sliding assists σ70-dependent promoter binding by *Escherichia coli* RNA polymerase. 2018; bioRxiv doi:13 December 2018, preprint: not peer reviewed 10.1101/494534.

[16] Suzuki Y. , Shin M., Yoshida A., Yoshimura S.H., Takeyasu K. Fast microscopical dissection of action scenes played by *Escherichia coli* RNA polymerase. FEBS Lett. 2012; 586:3187–3192. 22771906 10.1016/j.febslet.2012.06.033

[17] Wang F. , Redding S., Finkelstein I.J., Gorman J., Reichman D.R., Greene E.C. The promoter-search mechanism of escherichia coli RNA polymerase is dominated by three-dimensional diffusion. Nat. Struct. Mol. Biol. 2013; 20:174–181. 23262491 10.1038/nsmb.2472 PMC3565103

[18] Hofer B. , Muller D., Koster H. The pathway of *E. coli* RNA polymerase-promoter complex formation as visualized by footprinting. Nucleic Acids Res. 1985; 13:5995–6013. 3898021 10.1093/nar/13.16.5995 PMC321928

[19] Kovacic R.T. The 0 degree c closed complexes between escherichia coli RNA polymerase and two promoters, T7-A3 and lacUV5. J. Biol. Chem. 1987; 262:13654–13661. 3308880

[20] Mecsas J. , Cowing D.W., Gross C.A. Development of RNA polymerase-promoter contacts during open complex formation. J. Mol. Biol. 1991; 220:585–597. 1651395 10.1016/0022-2836(91)90102-c

[21] Schickor P. , Metzger W., Werel W., Lederer H., Heumann H. Topography of intermediates in transcription initiation of E.coli. EMBO J. 1990; 9:2215–2220. 2192861 10.1002/j.1460-2075.1990.tb07391.x PMC551945

[22] Spassky A. , Kirkegaard K., Buc H. Changes in the DNA structure of the lac UV5 promoter during formation of an open complex with *Escherichia coli* RNA polymerase. Biochem. 1985; 24:2723–2731. 3896305 10.1021/bi00332a019

[23] Davis C.A. , Capp M.W., Record M.T. Jr, Saecker R.M. The effects of upstream DNA on open complex formation by Escherichia coli RNA polymerase. Proc. Natl. Acad. Sci. U.S.A. 2005; 102:285–290. 15626761 10.1073/pnas.0405779102 PMC544287

[24] Davis C.A. , Bingman C.A., Landick R., Record M.T., Saecker R.M. Real-time footprinting of DNA in the first kinetically significant intermediate in open complex formation by Escherichia coli RNA polymerase. Proc. Natl. Acad. Sci. U.S.A. 2007; 104:7833–7838. 17470797 10.1073/pnas.0609888104 PMC1876533

[25] Boyaci H. , Chen J., Jansen R., Darst S.A., Campbell E.A. Structures of an RNA polymerase promoter melting intermediate elucidate DNA unwinding. Nature. 2019; 565:382–385. 30626968 10.1038/s41586-018-0840-5 PMC6399747

[26] Sreenivasan R. , Shkel I.A., Chhabra M., Drennan A., Heitkamp S., Wang H.C., Sridevi M.A., Plaskon D., McNerney C., Callies K. et al. Fluorescence-Detected conformational changes in duplex DNA in open complex formation by *Escherichia coli* RNA polymerase: upstream wrapping and downstream bending precede clamp opening and insertion of the downstream duplex. Biochem. 2020; 59:1565–1581. 32216369 10.1021/acs.biochem.0c00098 PMC7269339

[27] Zhang Y. , Feng Y., Chatterjee S., Tuske S., Ho M.X., Arnold E., Ebright R.H. Structural basis of transcription initiation. Science (New York, N.Y). 2012; 338:1076–1080. 10.1126/science.1227786 PMC3593053 23086998

[28] Basu R.S. , Warner B.A., Molodtsov V., Pupov D., Esyunina D., Fernandez-Tornero C., Kulbachinskiy A., Murakami K.S. Structural basis of transcription initiation by bacterial RNA polymerase holoenzyme. The J. Biol. Chem. 2014; 289:24549–24559. 24973216 10.1074/jbc.M114.584037 PMC4148879

[29] Zuo Y. , Steitz T.A. Crystal structures of the *E. coli* transcription initiation complexes with a complete bubble. Mol. Cell. 2015; 58:534–540. 25866247 10.1016/j.molcel.2015.03.010 PMC5567806

[30] Bae B. , Feklistov A., Lass-Napiorkowska A., Landick R., Darst S.A. Structure of a bacterial RNA polymerase holoenzyme open promoter complex. Elife. 2015; 4:e08504. 10.7554/eLife.08504 PMC4593229 26349032

[31] Feklistov A. , Darst S.A. Structural basis for promoter-10 element recognition by the bacterial RNA polymerase sigma subunit. Cell. 2011; 147:1257–1269. 22136875 10.1016/j.cell.2011.10.041 PMC3245737

[32] Kubori T. , Shimamoto N. A branched pathway in the early stage of transcription by escherichia coli RNA polymerase. J. Mol. Biol. 1996; 256:449–457. 8604130 10.1006/jmbi.1996.0100

[33] Saecker R.M. , Tsodikov O.V., McQuade K.L., Schlax P.E., Capp M.W., Record M.T. Kinetic studies and structural models of the association of e. coli sigma(70) RNA polymerase with the lambdaP(R) promoter: large scale conformational changes in forming the kinetically significant intermediates. J. Mol. Biol. 2002; 319:649–671. 12054861 10.1016/S0022-2836(02)00293-0

[34] Gries T.J. , Kontur W.S., Capp M.W., Saecker R.M., Record M.T. Jr One-step DNA melting in the RNA polymerase cleft opens the initiation bubble to form an unstable open complex. Proc. Natl. Acad. Sci. U.S.A. 2010; 107:10418–10423. 20483995 10.1073/pnas.1000967107 PMC2890804

[35] Kontur W.S. , Saecker R.M., Capp M.W., Record M.T. Late steps in the formation of E-coli RNA Polymerase-lambda P-R promoter open complexes: characterization of conformational changes by rapid [Perturbant] upshift experiments. J. Mol. Biol. 2008; 376:1034–1047. 18191943 10.1016/j.jmb.2007.11.064 PMC2396949

[36] Chen J. , Chiu C., Gopalkrishnan S., Chen A.Y., Olinares P.D.B., Saecker R.M., Winkelman J.T., Maloney M.F., Chait B.T., Ross W. et al. Stepwise promoter melting by bacterial RNA polymerase. Mol. Cell. 2020; 78:275–288. 32160514 10.1016/j.molcel.2020.02.017 PMC7166197

[37] Ko J. , Heyduk T. Kinetics of promoter escape by bacterial RNA polymerase: effects of promoter contacts and transcription bubble collapse. The Biochem. J. 2014; 463:135–144. 24995916 10.1042/BJ20140179

[38] Duchi D. , Bauer D.L.V., Fernandez L., Evans G., Robb N., Hwang L.C., Gryte K., Tomescu A., Zawadzki P., Morichaud Z. et al. RNA polymerase pausing during initial transcription. Mol. Cell. 2016; 63:939–950. 27618490 10.1016/j.molcel.2016.08.011 PMC5031556

[39] Henderson K.L. , Felth L.C., Molzahn C.M., Shkel I., Wang S., Chhabra M., Ruff E.F., Bieter L., Kraft J.E., Record M.T. Jr Mechanism of transcription initiation and promoter escape by E. coli RNA polymerase. Proc. Natl. Acad. Sci. U.S.A. 2017; 114:E3032–E3040. 28348246 10.1073/pnas.1618675114 PMC5393250

[40] Dulin D. , Bauer D.L.V., Malinen A.M., Bakermans J.J.W., Kaller M., Morichaud Z., Petushkov I., Depken M., Brodolin K., Kulbachinskiy A. et al. Pausing controls branching between productive and non-productive pathways during initial transcription in bacteria. Nat. Commun. 2018; 9:1478. 29662062 10.1038/s41467-018-03902-9 PMC5902446

[41] Susa M. , Kubori T., Shimamoto N. A pathway branching in transcription initiation in escherichia coli. Mol. Microbiol. 2006; 59:1807–1817. 16553885 10.1111/j.1365-2958.2006.05058.x PMC1413587

[42] Lerner E. , Chung S., Allen B.L., Wang S., Lee J., Lu S.W., Grimaud L.W., Ingargiola A., Michalet X., Alhadid Y. et al. Backtracked and paused transcription initiation intermediate of Escherichia coli RNA polymerase. Proc. Natl. Acad. Sci. U.S.A. 2016; 113:E6562–E6571. 27729537 10.1073/pnas.1605038113 PMC5087071

[43] Revyakin A. , Ebright R.H., Strick T.R. Promoter unwinding and promoter clearance by RNA polymerase: detection by single-molecule DNA nanomanipulation. Proc. Natl. Acad. Sci. U.S.A. 2004; 101:4776–4780. 15037753 10.1073/pnas.0307241101 PMC387324

[44] Duchi D. , Gryte K., Robb N.C., Morichaud Z., Sheppard C., Brodolin K., Wigneshweraraj S., Kapanidis A.N. Conformational heterogeneity and bubble dynamics in single bacterial transcription initiation complexes. Nucleic Acids Res. 2018; 46:677–688. 29177430 10.1093/nar/gkx1146 PMC5778504

[45] Ostrofet E. , Papini F.S., Dulin D. Correction-free force calibration for magnetic tweezers experiments. Sci. Rep. 2018; 8:15920. 30374099 10.1038/s41598-018-34360-4 PMC6206022

[46] Cnossen J.P. , Dulin D., Dekker N.H. An optimized software framework for real-time, high-throughput tracking of spherical beads. Rev. Sci. Instrum. 2014; 85:103712. 25362408 10.1063/1.4898178

[47] Dulin D. , Vilfan I.D., Berghuis B.A., Hage S., Bamford D.H., Poranen M.M., Depken M., Dekker N.H. Elongation-competent pauses govern the fidelity of a viral RNA-Dependent RNA polymerase. Cell Rep. 2015; 10:983–992. 25683720 10.1016/j.celrep.2015.01.031

[48] Papini F.S. , Seifert M., Dulin D. High-yield fabrication of DNA and RNA constructs for single molecule force and torque spectroscopy experiments. Nucleic Acids Res. 2019; 47:e144. 31584079 10.1093/nar/gkz851 PMC6902051

[49] Svetlov V. , Artsimovitch I. Purification of bacterial RNA polymerase: tools and protocols. Methods Mol. Biol. (Clifton, N.J.). 2015; 1276:13–29. 10.1007/978-1-4939-2392-2_2 PMC4324551 25665556

[50] Strick T.R. , Allemand J.F., Bensimon D., Bensimon A., Croquette V. The elasticity of a single supercoiled DNA molecule. Science (New York, N.Y). 1996; 271:1835–1837. 10.1126/science.271.5257.1835 8596951

[51] Lipfert J. , Koster D.A., Vilfan I.D., Hage S., Dekker N.H. Single-Molecule magnetic tweezers studies of type IB topoisomerases. DNA Topoisomerases: Methods Protoc. 2009; 582:71–89. 10.1007/978-1-60761-340-4_7 19763943

[52] Seifert M. , van Nies P., Papini F.S., Arnold J.J., Poranen M.M., Cameron C.E., Depken M., Dulin D. Temperature controlled high-throughput magnetic tweezers show striking difference in activation energies of replicating viral RNA-dependent RNA polymerases. Nucleic Acids Res. 2020; 48:5591–5602. 32286652 10.1093/nar/gkaa233 PMC7261197

[53] Truong C. , Oudre L., Vayatis N. Selective review of offline change point detection methods. Signal Process. 2020; 167:107299.

[54] Keogh E. , Chu S., Hart D., Pazzani M. An online algorithm for segmenting time series. Proceedings 2001 IEEE International Conference on Data Mining, 2001. 2001; 289–296.

[55] Schwarz G. Estimating dimension of a model. Ann. Stat. 1978; 6:461–464.

[56] Cowan G. Statistical Data Analysis. 1998; Oxford University Press.

[57] Press W.H. , Flannery B.P., Teukolsky S.A., Vetterling W.T. Numerical Recipes in C: The Art of Scientific Computing. 1992; Cambridge University Press.

[58] Cha S. A simple method for derivation of rate equations for enzyme-catalyzed reactions under the rapid equilibrium assumption or combined assumptions of equilibrium and steady state. J. Biol. Chem. 1968; 243:820–825. 5638598

[59] De Vlaminck I. , Henighan T., van Loenhout M.T., Pfeiffer I., Huijts J., Kerssemakers J.W., Katan A.J., van Langen-Suurling A., van der Drift E., Wyman C. et al. Highly parallel magnetic tweezers by targeted DNA tethering. Nano Lett. 2011; 11:5489–5493. 22017420 10.1021/nl203299e

[60] Sudhakar S. , Jachowski T.J., Kittelberger M., Maqbool A., Hermsdorf G.L., Abdosamadi M.K., Schäffer E. Supported solid lipid bilayers as a platform for single-molecule force measurements. Nano Lett. 2019; 19:8877–8886. 31746618 10.1021/acs.nanolett.9b03761

[61] Glasmastar K. , Larsson C., Hook F., Kasemo B. Protein adsorption on supported phospholipid bilayers. J. Colloid. Interface Sci. 2002; 246:40–47. 16290382 10.1006/jcis.2001.8060

[62] Collins B.E. , Ye L.F., Duzdevich D., Greene E.C. DNA curtains: novel tools for imaging protein-nucleic acid interactions at the single-molecule level. Methods Cell Biol. 2014; 123:217–234. 24974030 10.1016/B978-0-12-420138-5.00012-4

[63] Ostrofet E. , Papini F.S., Malinen A.M., Dulin D. Joo C. , Rueda D. Biophysics of RNA-Protein Interactions. 2019; NY Springer 109–141.

[64] Brutzer H. , Luzzietti N., Klaue D., Seidel R. Energetics at the DNA supercoiling transition. Biophys. J. 2010; 98:1267–1276. 20371326 10.1016/j.bpj.2009.12.4292 PMC2849096

[65] Vilfan I.D. , Lipfert J., Koster D.A., Lemay S.G., Dekker N.H. Magnetic tweezers for single-molecule experiments. Handb. Single-Mol. Biophys. 2009; 371–395.

[66] Charvin G. , Allemand J.F., Strick T.R., Bensimon D., Croquette V. Twisting DNA: single molecule studies. Contemp. Phys. 2004; 45:383–403.

[67] Dulin D. , Cui T.J., Cnossen J., Docter M.W., Lipfert J., Dekker N.H. High spatiotemporal-resolution magnetic tweezers: calibration and applications for DNA dynamics. Biophys. J. 2015; 109:2113–2125. 26588570 10.1016/j.bpj.2015.10.018 PMC4656881

[68] Lansdorp B.M. , Saleh O.A. Power spectrum and allan variance methods for calibrating single-molecule video-tracking instruments. Rev. Sci. Instrum. 2012; 83:025115. 22380133 10.1063/1.3687431 PMC3306435

[69] Xie S.N. Single-molecule approach to enzymology. Single Mol. 2001; 2:229–236.

[70] Tsodikov O.V. , Record M.T. General method of analysis of kinetic equations for multistep reversible mechanisms in the single-exponential regime: application to kinetics of open complex formation between esigma70 RNA polymerase and lambdaP(R) promoter DNA. Biophys. J. 1999; 76:1320–1329. 10049315 10.1016/S0006-3495(99)77294-2 PMC1300111

[71] Anderson P. , Bauer W. Supercoiling in closed circular DNA - dependence upon ion type and concentration. Biochem. 1978; 17:594–601. 623732 10.1021/bi00597a006

[72] Wang J.Y. , Drlica K., Syvanen M. Monovalent cations differ in their effects on transcription initiation from a sigma-70 promoter of *Escherichia coli*. Gene. 1997; 196:95–98. 9322745 10.1016/s0378-1119(97)00207-2

[73] Cruz-León S. , Vanderlinden W., Müller P., Forster T., Staudt G., Lin Y.-Y., Lipfert J., Schwierz N. Twisting DNA by salt. Nucleic Acids Res. 2022; 50:5726–5738. 35640616 10.1093/nar/gkac445 PMC9177979

[74] Revyakin A. , Liu C., Ebright R.H., Strick T.R. Abortive initiation and productive initiation by RNA polymerase involve DNA scrunching. Science (New York, N.Y). 2006; 314:1139–1143. 10.1126/science.1131398 PMC2754787 17110577

[75] Hsu L.M. Monitoring abortive initiation. Methods. 2009; 47:25–36. 18948204 10.1016/j.ymeth.2008.10.010 PMC2647590

[76] Yang Z. Hofmeister effects: an explanation for the impact of ionic liquids on biocatalysis. J. Biotechnol. 2009; 144:12–22. 19409939 10.1016/j.jbiotec.2009.04.011

[77] Kontur W.S. , Capp M.W., Gries T.J., Saecker R.M., Record M.T. Jr Probing DNA binding, DNA opening, and assembly of a downstream clamp/jaw in *Escherichia coli* RNA polymerase-lambdaP(R) promoter complexes using salt and the physiological anion glutamate. Biochem. 2010; 49:4361–4373. 20201585 10.1021/bi100092a PMC2893406

[78] Cheng X. , Guinn E.J., Buechel E., Wong R., Sengupta R., Shkel I.A., Record M.T. Jr Basis of protein stabilization by k glutamate: unfavorable interactions with carbon, oxygen groups. Biophys. J. 2016; 111:1854–1865. 27806267 10.1016/j.bpj.2016.08.050 PMC5103011

[79] Roe J.H. , Burgess R.R., Record M.T. Jr Temperature dependence of the rate constants of the escherichia coli RNA polymerase-lambda PR promoter interaction. Assignment of the kinetic steps corresponding to protein conformational change and DNA opening. J. Mol. Biol. 1985; 184:441–453. 3900414 10.1016/0022-2836(85)90293-1

[80] Buc H. , McClure W.R. Kinetics of open complex formation between escherichia coli RNA polymerase and the lac UV5 promoter. Evidence for a sequential mechanism involving three steps. Biochem. 1985; 24:2712–2723. 3896304 10.1021/bi00332a018

[81] Winzor D.J. , Jackson C.M. Interpretation of the temperature dependence of equilibrium and rate constants. J. Mol. Recognit. 2006; 19:389–407. 16897812 10.1002/jmr.799

[82] Vindigni A. , Di Cera E. Release of fibrinopeptides by the slow and fast forms of thrombin. Biochem. 1996; 35:4417–4426. 8605191 10.1021/bi952834d

[83] Mozurkewich M. , Benson S.W. Negative activation energies and curved arrhenius plots. 1. Theory of reactions over potential wells. The J. Phys. Chem. 1984; 88:6429–6435.

[84] Plaskon D.M. , Henderson K.L., Felth L.C., Molzahn C.M., Evensen C., Dyke S., Shkel I.A., Record M.T. Jr Temperature effects on RNA polymerase initiation kinetics reveal which open complex initiates and that bubble collapse is stepwise. Proc. Natl. Acad. Sci. U.S.A. 2021; 118:e2021941118. 34290140 10.1073/pnas.2021941118 PMC8325161

[85] Ha J.H. , Capp M.W., Hohenwalter M.D., Baskerville M., Record M.T. Jr Thermodynamic stoichiometries of participation of water, cations and anions in specific and non-specific binding of lac repressor to DNA. Possible thermodynamic origins of the “glutamate effect” on protein-DNA interactions. J. Mol. Biol. 1992; 228:252–264. 1447786 10.1016/0022-2836(92)90504-d

[86] Saecker R.M. , Chen J., Chiu C.E., Malone B., Sotiris J., Ebrahim M., Yen L.Y., Eng E.T., Darst S.A. Structural origins of Escherichia coli RNA polymerase open promoter complex stability. Proc. Natl. Acad. Sci. U.S.A. 2021; 118:e2112877118. 34599106 10.1073/pnas.2112877118 PMC8501879

[87] Malinen A.M. , Bakermans J., Aalto-Setälä E., Blessing M., Bauer D.L.V., Parilova O., Belogurov G.A., Dulin D., Kapanidis A.N. Real-time single-molecule studies of RNA polymerase–promoter open complex formation reveal substantial heterogeneity along the promoter-opening pathway. J. Mol. Biol. 2022; 434:167383. 34863780 10.1016/j.jmb.2021.167383 PMC8783055

