# Supplementary material for: Quantitative parameters of bacterial RNA polymerase open-complex formation, stabilization and disruption on a consensus promoter
Source: Nucleic Acids Res. 2022 Jul 12;50(13):7511–28. doi: 10.1093/nar/gkac560 (PMC9303404; doi:10.1093/nar/gkac560)
Supplement: gkac560_Supplemental_Files [file gkac560_supplemental_files.zip › Bera_etal_SI.pdf]

Supplementary information for:

Quantitative parameters of bacterial RNA polymerase open-complex formation, stabilization and disruption on a consensus promoter

Subhas C. Bera[1,†], Pim P. B. America[2,†], Santeri Maatsola[3], Mona Seifert[1], Eugeniu Ostrofet[1], Jelmer Cnossen[4], Monika Spermann[1], Flávia Stal-Papini[1], Martin Depken[5], Anssi M. Malinen[3], David Dulin[1,2,#]

1 Junior Research Group 2, Interdisciplinary Center for Clinical Research, Friedrich Alexander University Erlangen-Nürnberg (FAU), Cauerstr. 3, 91058 Erlangen, Germany.

2 Department of Physics and Astronomy, and LaserLaB Amsterdam, Vrije Universiteit Amsterdam, De Boelelaan 1081, 1081 HV, Amsterdam, The Netherlands

3 Department of Life Technologies, University of Turku, Tykistökatu 6A, 6th floor, 20520 Turku, Finland

4 Delft Center for Systems and Control, Delft University of Technology, Delft, the Netherlands

5 Department of Bionanoscience, Kavli Institute of Nanoscience, Delft University of Technology, Van der Maasweg 9, 2629 HZ Delft, The Netherlands

† These authors contributed equally to this work.

# Correspondence may be addressed to: d.dulin@vu.nl

This Supplementary Information contains

Supplementary Notes 1 to 2

Supplementary Figures 1 to 11

Table S1 to S4

Supplementary notes 1: Kinetic description of double exponential OS dwell time distributions

In this section, we discuss how we evaluated which kinetic model best describes the open state (OS) dwell time distribution, or in other words, the OS disassembly time. First note that the unbinding distribution for a kinetic model can in general be obtained by evaluating the first passage time distributions of all the paths that result in unbinding. If we assume the specific transition from x to y occurs with rate constant $k_{x\to y}$, when the total rate constant of all transitions starting from state x is $k_{\mathrm{tot,x}}$, we can write the first passage time distribution for the transition as

$$p_{x\to y}(t) = p_{x\to y} k_{\mathrm{tot,x}} e^{-k_{\mathrm{tot,x}} t} = k_{x\to y} e^{-k_{\mathrm{tot,x}} t}.$$

Here we have used that the (splitting) probability for making the transition to x before any of the other transitions is

$$p_{x\to y} = \frac{k_{x\to y}}{k_{\mathrm{tot,x}}}.$$

The first passage time distribution of a path through multiple states can be found as the convolution of the first passage time distributions between the successive states. To enable to combine successive transitions by simply multiplying probability densities (rather than performing convolutions), we move to Laplace space, and write the general probability distribution as

$$\phi_x(s) = \int_0^\infty e^{-st} p_x(t) \mathrm{d}t = \frac{k_x}{s + k_{\mathrm{tot}}}$$

Model 1: open complex starts from RP_I and dissociates from RP_C

We first evaluate the most straight-forward model for OS dissociation, i.e. the holo enters the OS from RP_I to reach RP_O, and walks back the kinetic pathway to dissociate from RP_C, such as

$$\mathrm{R + P} \underset{k_{-1}}{\longleftarrow} \mathrm{RP_C} \underset{k_{-2}}{\overset{k_2}{\rightleftharpoons}} \mathrm{RP_I} \underset{k_{-3}}{\overset{k_3}{\rightleftharpoons}} \mathrm{RP_O} \qquad \text{(S1)}$$

The system converts from the RP_I to RP_C or RP_O with rate constant k_{-2} or k_3 respectively, and can get repeatedly reabsorbed back into RP_I with rate constant k_2 or k_{-3} before the holo dissociates from RP_C with rate constant k_{-1}. Of note, this model can be interpreted as an extension of **Model 2, Assumption 3**, where k_4 is subdivided into k_2, k_{-2} and k_{-1}.

Using this kinetic model, we have the following transition-time distributions

$\phi_{-1} = \frac{k_{-1}}{k_{-1}+k_2+s}$, $\phi_2 = \frac{k_2}{k_{-1}+k_2+s}$, $\phi_{-2} = \frac{k_{-2}}{k_3+k_{-2}+s}$

$\phi_3 = \frac{k_3}{k_3+k_{-2}+s}$, $\phi_{-3} = \frac{k_{-3}}{k_{-3}+s}$

By summing transition-time distributions of escape paths, enumerated by the number n of absorptions into RP_O before escaping, we can write down the full escape-time distribution. Starting from RP_I, the escape-time distribution is

$$\Psi_{RP_I \to R+P}(s) = \phi_{-1}\phi_{-2} \sum_{m_1}^{\infty} \sum_{m_2}^{\infty} \frac{(m_1 + m_2)!}{m_1! \, m_2!} (\phi_2 \phi_{-2})^{m_1} (\phi_3 \phi_{-3})^{m_2}$$

Defining $m = m_1 + m_2$, we have

$$\Psi_{RP_I \to R+P}(s) = \phi_{-1}\phi_{-2} \sum_{m}^{\infty} \sum_{m_1}^{m} \frac{m!}{m_1! \, (m - m_1)!} (\phi_2 \phi_{-2})^{m} (\phi_3 \phi_{-3})^{m-m_1}$$

Using the binomial theorem, we can write

$$\Psi_{RP_I \to R+P}(s) = \phi_{-1}\phi_{-2} \sum_{m}^{\infty} (\phi_2\phi_{-2} + \phi_3\phi_{-3})^m = \frac{\phi_{-1}\phi_{-2}}{1 - \phi_2\phi_{-2} - \phi_3\phi_{-3}}$$

$$= \frac{k_1 k_{-2}(k_{-3} + s)}{(k_{-1} + k_2 + s)(k_3 + k_{-2} + s)(k_{-3} + s) - k_2 k_{-2}(k_{-3} + s) - k_3 k_{-3}(k_{-1} + k_2 + s)}$$

$$= \frac{k_{-1} k_{-2}(k_{-3} + s)}{(s + s_1)(s + s_2)(s + s_3)}$$

Where s_1, s_2 and s_3 are defined as minus the roots of the denominator. As we have three roots, we can write $\Psi_{RP_I \to R+P}$ as the sum of three Laplace transformed exponentials using partial fraction decomposition, such as

$$\Psi_{RP_I \to R+P}(s) = \frac{A_1}{s + s_1} + \frac{A_2}{s + s_2} + \frac{A_3}{s + s_3}$$

$$= \frac{A_1(s + s_2)(s + s_3) + A_2(s + s_1)(s + s_3) + A_3(s + s_1)(s + s_2)}{(s + s_1)(s + s_2)(s + s_3)}$$

$$= \frac{s^2(A_1 + A_2 + A_3) + s[A_1(s_2 + s_3) + A_2(s_1 + s_3) + A_3(s_1 + s_2)] + A_3 s_1 s_2 + A_1 s_2 s_3 + A_2 s_1 s_3}{(s + s_1)(s + s_2)(s + s_3)}$$

Comparison of the numerators gives us for the following relations

$$A_1 + A_2 + A_3 = 0$$

$$A_1(s_2+s_3)+A_2(s_1+s_3)+A_3(s_1+s_2)=k_{-1}k_{-2}$$

$$A_3s_1s_2+A_1s_2s_3+A_2s_1s_3=k_{-1}k_{-2}k_{-3}$$

Considering the probability density function must be normalized in the time domain, we can write it in the form

$$\mathrm{P}_{RP_I\rightarrow R+P}(t)=p_1s_1e^{-s_1t}+p_2s_2e^{-s_2t}+(1-p_1-p_2)s_3e^{-s_3t}$$

Note that in this notation $A_1=p_1s_1$, $A_2=p_2s_2$ and $A_3=(1-p_1-p_2)s_3$, so we can rewrite the relations from the numerator to

$$p_1s_1+p_2s_2+(1-p_1-p_2)s_3=0 \quad \text{(S2)}$$

$$p_1s_1(s_2+s_3)+p_2s_2(s_1+s_3)+(1-p_1-p_2)s_3(s_1+s_2)=k_{-1}k_{-2} \quad \text{(S3)}$$

$$s_1s_2s_3=k_{-1}k_{-2}k_{-3} \quad \text{(S4)}$$

From **Equations S2** and **S3**, we get

$$p_1=\frac{s_2s_3-k_{-1}k_{-2}}{(s_3-s_1)(s_2-s_1)} \quad \text{(S5)}$$

$$p_2=-\frac{s_1s_3-k_{-1}k_{-2}}{(s_3-s_2)(s_2-s_1)} \quad \text{(S6)}$$

$$p_3=1-p_1-p_2=\frac{s_1s_2-k_{-1}k_{-2}}{(s_3-s_2)(s_3-s_1)} \quad \text{(S7)}$$

We now need to evaluate the signs of the exponential weights to determine the nature of the distribution, considering that the kinetic rate constants must be positive.

First note that we only consider distributions with real and positive exponential rate constants (resulting in exponential decay) such that we can choose $s_3>s_2>s_1>0$. Starting from **Equations S2** and **S3**, we obtain

$$k_{-1}k_{-2}=-p_1(s_3-s_1)(s_2-s_1)+s_2s_3$$

Considering that $k_{-1},k_{-2}>0$ we find the condition

$$p_1>\frac{s_2s_3}{(s_3-s_1)(s_2-s_1)}>0 \quad \text{(S8)}$$

Of note, we have $\frac{s_2}{(s_2-s_1)}>1$ and $\frac{s_3}{(s_3-s_1)}>1$ because $s_3>s_2>s_1>0$. We can therefore rewrite **Equation S8** such as

$p_1 > \frac{s_3}{s_3 - s_1} > 1$ and $p_1 > \frac{s_2}{s_2 - s_1} > 1$

The numerator of **Equation S6** must verify

$k_{-1}k_{-2} - s_1 s_3 = (s_2 - s_1)[-p_1(s_3 - s_1) + s_3] < 0$ and the denominator is positive, therefore $p_2 < 0$.

Furthermore, the numerator of **Equation S7** must verify

$s_1 s_2 - k_{-1}k_{-2} = (s_3 - s_1)[p_1(s_2 - s_1) - s_2] > 0$, the denominator is positive and therefore $p_3 > 0$.

In conclusion, we have two exponentials with a positive weight and one with a negative rate constant. The global distribution is therefore the combination of a peaked and an exponential distribution.

Model 3: Dissociation from RP_O

As dissociation from RP_C (Model 1) fails at describing the double exponential distribution of the OS dwell times, we propose another model, where the holo dissociates from RP_O.

$$\mathrm{RP_I} \underset{k_{-3}}{\overset{k_3}{\rightleftharpoons}} \mathrm{RP_O} \overset{k_4}{\rightarrow} \mathrm{R} + \mathrm{P} \qquad \text{(S9)}$$

The system converts from the RP_I to RP_O with rate constant k_3, and can get temporarily and repeatedly reabsorbed back into RP_I with rate constant k_{-3}, before the holo dissociates from RP_O with rate constant k_4. In the following section, we calculate the escape time distribution from the double bound states system for two cases, i.e. the OS starts in either RP_O or RP_I.

The particular transition-time distributions for **Equation S9** are

$$\phi_4(s) = \frac{k_4}{s + k_4 + k_{-3}}, \qquad \phi_3(s) = \frac{k_3}{s + k_3}, \qquad \phi_{-3}(s) = \frac{k_{-3}}{s + k_4 + k_{-3}}.$$

By summing transition-time distributions of escape paths, enumerated by the number n of absorptions into RP_O before escaping, we can write down the full escape-time distribution, which differs depending on the starting point of the OS.

Model 3, Case 1: the OS starts in RP_O

Here, the OS starts in RP_O, and therefore we have

$$\Psi_{RP_O\to R+P}(s) = \phi_4(s)\sum_{n=0}^{\infty}\left(\phi_3(s)\phi_{-3}(s)\right)^n = \frac{\phi_4(s)}{1-\phi_{-3}(s)\phi_3(s)} = \frac{k_4(s+k_3)}{(s+k_3)(s+k_4+k_{-3})-k_{-3}k_3}$$

This escape time distribution is readily converted back from Laplace to real space as

$$P_{RP_O\to R+P}(t) = p_+k_+e^{-k_+t} + (1-p_+)k_-e^{-k_-t}.$$

With $p_{i,+} = \frac{A_{i,+}}{k_+}$ and $1-p_+ = \frac{A_{i,-}}{k_-}$.

We have the two effective rate constants

$$k_\pm = \frac{1}{2}(k \pm \Delta k), \qquad k = k_{-3}+k_3+k_4, \qquad \Delta k = \sqrt{k^2-4k_3k_4}$$

By definition, we have $k_\pm$

$$k_+ + k_- = k_{-3}+k_3+k_4$$

If we now find the roots of the denominator to factorize it and use partial-fraction decomposition we find that $\Psi_{RP_O\to R+P}(s)$ can be written in the form of two Laplace transformed exponential.

$$\Psi_{RP_O\to R+P}(s) = \frac{k_4(s+k_3)}{(s+k_3)(s+k_4+k_{-3})-k_{-3}k_3} = \frac{s(A_{O,+}+A_{O,-})+A_{O,+}k_- + A_{O,-}k_+}{(s+k_+)(s+k_-)}$$
$$= \frac{A_{O,+}}{s+k_+} + \frac{A_{O,-}}{s+k_-}$$

The numerator of $\Psi_{RP_O\to R+P}$ must follow

$A_{O,+}+A_{O,-} = k_4$ and $A_{O,+}k_- + A_{O,-}k_+ = k_3k_4$

Which can also be written as

$k_4 = p_+k_+ + (1-p_+)k_-$ and $k_+k_- = k_3k_4$

From which can be derived

$p_{O,+} = \frac{k_4-k_-}{k_+-k_-}$ and $p_{O,-} = 1-p_{O,+} = \frac{k_+-k_4}{k_+-k_-}$

As we have per definition $k_+ > k_-$; $k_4 = p_+k_+ + (1-p_+)k_-$ and the kinetic rate constants should be positive, we can derive that $k_+ > k_4 > k_-$ and therefore the two exponential weights $p_{O,+/-}$ must be both

positive. The kinetic rate constants for this model can be found from the double exponential fit parameters with the following conversion relations.

$k_4 = p_+ k_+ + (1 - p_+) k_-$

$k_3 = \frac{k_+ k_-}{k_4}$

$k_{-3} = \frac{(k_+ - k_4)(k_4 - k_-)}{k_4}$

This model is however inconsistent with the start of the OS in RP_I, as described in the literature, and can therefore be discarded.

Model 3, Case 2: the OS starts in RP_I

Starting in OS from RP_I, we need to add an extra transition from RP_I to RP_O state

$$\Psi_{RP_I \to R+P}(s) = \phi_3(s)\Psi_{RP_O \to R+P}(s) = \frac{\phi_3(s)\phi_4(s)}{1 - \phi_{-3}(s)\phi_3(s)} = \frac{k_3 k_4}{(s + k_3)(s + k_4 + k_{-3}) - k_{-3} k_3}$$
$$= \frac{s(A_{I,+} + A_{I,-}) + A_{I,+} k_- + A_{I,-} k_+}{(s + k_+)(s + k_-)} = \frac{A_{I,+}}{s + k_+} + \frac{A_{I,-}}{s + k_-}.$$

Similarly to Case 1, this escape time distributions is readily converted back from Laplace to real space as

$$P_{RP_I \to R+P}(t) = p_+ k_+ e^{-k_+ t} + (1 - p_+) k_- e^{-k_- t}.$$

With $p_{i,+} = \frac{A_{i,+}}{k_+}$ and $1 - p_+ = \frac{A_{i,-}}{k_-}$.

We have the two effective rate constants

$$k_\pm = \frac{1}{2}(k \pm \Delta k), \qquad k = k_{-3} + k_3 + k_4, \qquad \Delta k = \sqrt{k^2 - 4 k_3 k_4}$$

Of note, these effective rate constants are independent from the starting position, i.e. either RP_O or RP_I.

From the numerator of $\Psi_{RP_I \to R+P}$, we have

$p_+ k_+ + (1 - p_+) k_- = 0$ and $k_+ k_- = k_3 k_4$

From which can be derived

$p_{I,+} = -\frac{k_-}{k_+ - k_-} < 0,$ $p_{I,-} = 1 - p_{O,+} = \frac{k_+}{k_+ - k_-} \geq 0$

In this case we find that one of the exponential weights is negative, which leads to a peaked distribution where a minimum of two successive steps is needed before holo dissociation (1,2). We clearly observe a double exponential distribution in **Figure 1D**, and therefore Model 2, Case 2 cannot describe the data.

Model 4: open complex starts from RP_I, dissociation from RP_O and RP_I considered

After concluding that **Model 1 and 3** are not suitable to describe the OS dwell time distribution, we propose a third model, which allows the holo to dissociated from both RP_I and RP_O, while the OS starts in RP_I (**Table 1**).

$$\mathrm{R+P} \overset{k_5}{\leftarrow} \mathrm{RP_I} \underset{k_{-3}}{\overset{k_3}{\rightleftharpoons}} \mathrm{RP_O} \overset{k_4}{\rightarrow} \mathrm{R+P} \tag{S10}$$

Using the formalism described above, the particular transition-time distributions for the new model (**Equation S10**) are

$\phi_5 = \frac{k_5}{k_5+k_3+s}$, $\phi_3 = \frac{k_3}{k_5+k_3+s}$, $\phi_{-3} = \frac{k_{-3}}{k_4+k_{-3}+s}$, $\phi_4 = \frac{k_4}{k_4+k_{-3}+s}$

In this model we start from RP_I and there are escape-routes with we have that the path to unbinding is

$$\Psi_{RP_I\to R+P}(s) = (\phi_5+\phi_3\phi_4)\sum_{n=0}^{\infty}(\phi_3\phi_{-3})^n = \frac{\phi_5+\phi_3\phi_4}{1-\phi_3\phi_{-3}} = \frac{k_5(k_4+k_{-3}+s)+k_3k_4}{(k_4+k_{-3}+s)(k_5+k_3+s)-k_3k_{-3}}$$
$$= \frac{k_5k_4+k_5k_{-3}+k_3k_4+k_5s}{(s+k_+)(s+k_-)}$$

In the last step, we factorized the denominator with the roots

$$k_\pm = \frac{1}{2}(k\pm\Delta k), \qquad k = k_{-3}+k_3+k_4+k_5 \ ,$$

$$\Delta k = \sqrt{k^2-4[(k_5+k_3)(k_4+k_{-3})-k_3k_{-3}]} = \sqrt{k^2-4[k_5k_4+k_5k_{-3}+k_3k_4]}$$

Using partial-fraction decomposition, we find that we can write $\Psi_{RP_I\to R+P}$ in the form

$$\Psi_{RP_I\to R+P} = \frac{k_5k_4+k_5k_{-3}+k_3k_4+k_5s}{(s+k_+)(s+k_-)} = \frac{s(A_++A_-)+A_+k_-+A_-k_+}{(s+k_+)(s+k_-)} = \frac{A_+}{s+k_+}+\frac{A_-}{s+k_-}$$

Where A_+ and A_- are a combination of the kinetic rate constants. Note that if we transform this form of $\Psi_{RP_I\to R+P}$ back from Laplace space to real space, we get a double exponentials probability distribution function

$$P_{RP_I \to R+P}(t) = A_+ e^{-k_+ t} + A_- e^{-k_- t} = p_+ k_+ e^{-k_+ t} + (1 - p_+) k_- e^{-k_- t}$$

To get the coefficients A_+ and A_-, note from the numerator of $\Psi_{RP_I \to R+P}$ that

$$A_+ + A_- = k_5 \quad \text{and} \quad A_+ k_- + A_- k_+ = k_5 k_4 + k_5 k_{-3} + k_3 k_4 \qquad \text{(S11, S12)}$$

From the numerator of $\Psi_{RP_I \to R+P}$ we find

$$k_+ k_- = k_5 k_4 + k_5 k_{-3} + k_3 k_4 \qquad k_+ + k_- = k_{-3} + k_3 + k_4 + k_5 \qquad \text{(S13, S14)}$$

Therefore, we can write

$$p_+ = \frac{A_+}{k_+} = \frac{k_5 - k_-}{k_+ - k_-} \quad \text{and} \quad p_- = 1 - p_+ = \frac{k_+ - k_5}{k_+ - k_-}$$

To extract the values of the kinetic rate constants in the model from the double exponential fits, we need expressions for the kinetic rate constants in terms of the fitting parameters.

First note that

$$k_5 = A_+ + A_- = p_+ k_+ + (1 - p_+) k_- \qquad \text{(S15)}$$

Secondly, consider that from **Equation S13** and **S14** we obtain

$$(k_+ + k_-)(k_3 + k_5) - k_+ k_- = {k_3}^2 + 2 k_3 k_5 + {k_5}^2 + k_3 k_{-3}$$

Rewriting this relation gives

$$k_{-3} = \frac{-(k_3 + k_5)^2 + (k_+ + k_-)(k_3 + k_5) - k_+ k_-}{k_3} \qquad \text{(S16)}$$

$$= \frac{(k_+ - (k_3 + k_5))((k_3 + k_5) - k_-)}{k_3}$$

and using $k_+ + k_- = k_{-3} + k_3 + k_4 + k_5$ we get

$$k_4 = \frac{k_3 k_5 + {k_5}^2 - k_5(k_+ + k_-) + k_+ k_-}{k_3} = k_5 - \frac{(k_+ - k_5)(k_5 - k_-)}{k_3} \qquad \text{(S17)}$$

Now we have derived solutions for k_5, k_{-3} and k_4 in terms of p_+, k_+, k_-, k_3. We could however not retrieve a complete set of conversion relations for fit parameters p_+, k_+, k_- to kinetic parameters k_3, k_{-3}, k_4, k_5 without making an assumption. This is simply because a direct mapping of three parameters (p_+, k_+, k_-) to four parameters (k_3, k_{-3}, k_4, k_5) cannot be made.

Model 4, Assumption 1: RP_I and RP_O in rapid equilibrium

If we assume that the rate constants between states RP_I and RP_O (k_3 and k_{-3}) are much higher than the rate constants to unbind from either of the two states (k_4 and k_5), we can assume that the transitions between the states are equilibrated and the detailed balance holds between the two states

$$p_{RP_I} k_3 = p_{RP_O} k_{-3}, \qquad p_{RP_I} + p_{RP_O} = 1,$$

where p_{RP_I} and p_{RP_O} are the equilibrated fractions in the RP_I and RP_O state respectively. In this case, we have a double exponential unbinding distribution with $p_+ = p_{RP_I}$ and $1 - p_+ = p_{RP_O}$. Thus, we have $k_{-3} = \frac{p_{RP_I}}{p_{RP_O}} k_3 = \frac{p_+}{1-p_+} k_3$. Substitution of this relation in the general expression for k_{-3} (**Equation S16**) gives

$$\frac{p_+}{1-p_+} k_3 = \frac{-(k_3+k_5)^2 + k_5(k_+ + k_-) + k_3(k_+ + k_-) - k_+ k_-}{k_3}$$

$$0 = {k_3}^2 \left(1 + \frac{p_+}{1-p_+}\right) + k_3(+2k_5 - k_+ - k_-) + k_+ k_- - k_5(k_+ + k_-) + {k_5}^2$$

And $1 + \frac{p_+}{1-p_+} = \frac{1}{1-p_+}$, thus

$$k_3 = \frac{1}{2}(k_+ + k_- - 2k_5)(1-p_+) \pm \frac{1}{2}(1-p_+)\sqrt{(k_+ + k_- - 2k_5)^2 - \frac{4(k_+ - k_5)(k_- - k_5)}{1-p_+}}$$

And we already derived relations for k_5 (**Equation S15**) and k_4 (**Equation S17**) in terms of p_+, k_+, k_-, k_3, so this equation gives us a complete set of conversion relations.

$$k_5 = p_+ k_+ + (1-p_+) k_-$$

$$k_3 = \frac{1}{2}(k_+ + k_- - 2k_5)(1-p_+) + \frac{1}{2}(1-p_+)\sqrt{(k_+ + k_- - 2k_5)^2 - \frac{4(k_+ - k_5)(k_- - k_5)}{1-p_+}}$$

$$k_4 = k_5 - \frac{(k_+ - k_5)(k_5 - k_-)}{k_3}$$

To ensure $k_3, k_{-3}, k_4, k_5 \geq 0$ for this assumption, we need to take the expression for k_3 with the plus-sign before the second term and the expression under the square root should be positive or zero. For this second condition, we must have $k_+ \geq k_5 \geq k_-$ and $p_+ \geq 0$, which boils down to $p_+ \geq 0$ and $k_+ \geq k_-$ and these conditions are per definition true for a double exponential fit.

Additionally, to ensure $k_4 > 0$ we have the boundary

$k_4 = k_5 - \frac{(k_+ - k_5)(k_5 - k_-)}{k_3} > 0$ thus $k_3 > \frac{(k_+ - k_5)(k_5 - k_-)}{k_5}$

Filling in this boundary in the expression for k_3 and using the expression for k_5 (**Equation S15**) we can get the upper boundary on p_+

$$p_+ < \frac{k_+ k_+}{k_5(k_+ + k_- - k_5)} = \frac{\sqrt{k_+ k_+}}{k_+ - k_-}$$

By evaluating this boundary for the double exponential fit parameters obtained, we could directly argue whether this model assumption would mathematically work or not.

Model 4, Assumption 2: **No reverse reaction from RP_O to RP_I**

For the assumption $k_{-3} = 0$, we find from the general expression for k_{-3} (**Equation S16**) that $k_+ = k_3 + k_5$ or $k_- = k_3 + k_5$. Considering $k_3 + k_5 > k_4$ as we expect unbinding to be faster from RP_I then from RP_O, we take $k_+ = k_3 + k_5$ and obtain

$$k_4 = k_5 - \frac{((k_3 + k_5) - k_5)(k_5 - k_-)}{k_3} = k_-$$

Taking into account the expression for k_5 (**Equation S15**), we have a complete set of conversion relations.

$k_5 = p_+ k_+ + (1 - p_+)k_-$

$k_4 = k_-$

$k_3 = k_+ - k_5$

For $k_3 \geq 0$ we need to have $k_+ \geq k_5$, which boils down to $p_+ \geq 0$ and $k_+ \geq k_-$ and these conditions are per definition true for a double exponential fit.

Model 4, Assumption 3: dissociation from only RP_I

Alternatively, we can assume there is no dissociation from RP_O, thus $k_4 = 0$. Under this assumption we get $k_5 = \frac{(k_+ - k_5)(k_5 - k_-)}{k_3}$ from **Equation S17** and $k_+ k_- = k_5 k_{-3}$ from **Equation S13**, thus we have

$k_5 = p_+ k_+ + (1 - p_+)k_-$

$k_3 = \frac{(k_+ - k_5)(k_5 - k_-)}{k_5}$

$k_{-3} = \frac{k_+ k_-}{k_5}$

In this case, we must have $k_+ \geq k_5 \geq k_-$ to ensure $k_3, k_{-3}, k_4, k_5 \geq 0$, which means $p_+ \geq 0$ and $k_+ \geq k_-$ and these conditions are per definition true for a double exponential fit. The evaluation of the kinetic rate constants calculated from the two exponential fits for the different assumptions to **Model 4** demonstrated that **Assumption 3** was the best proposed model for the OS disassembly times (**Results, Table 1**).

Supplementary Notes 2: Kinetic description of single exponential OS dwell time distributions

Using Model 3, Assumption 3 to describe the OS dwell times (**Table 1**), the OS dwell times distributions described by a single exponential probability distribution function report on the direct dissociation from RP_I with rate constant k_5, i.e. the holo did not transition to RP_O.

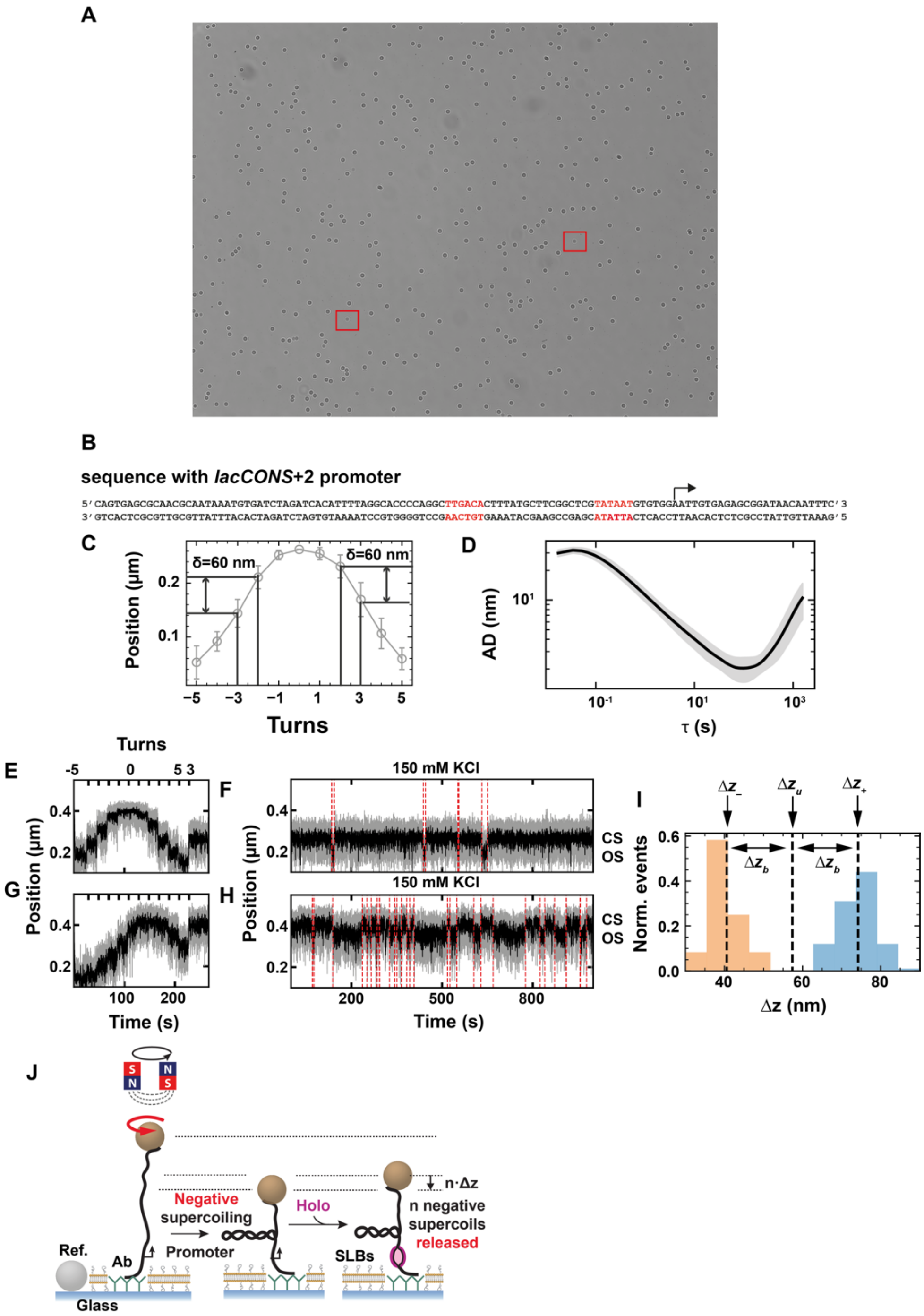

Supplementary Figure 1. Field of view, DNA sequence and effects of torque on open complex dynamics. (A) Typical field of view from a high throughput magnetic tweezers experiment, where 1 µm diameter MyOne magnetic beads (dark circles) are tethered to the glass surface by a ~1.4 kbp coilable DNA construct (**Material and Methods**). The polystyrene reference beads (1.1 µm diameter) are surrounded by a red square. **(B)** *lacCONS*+2 promoter sequence that is inserted in the dsDNA construct used in the MT experiment. The -10 element and -35 element are indicated in red, and the transcription start site and the

holo transcription direction are indicated by an arrow. **(C)** Estimation of change in extension for every turn from where we choose the linear regime as indicated and calculate $\delta = 60\ nm$. The error bar is one standard deviation from mean taken from seven traces. **(D)** Allan deviation calculated from a positively supercoiled 1.4 kb long dsDNA tethering a MyOne magnetic bead experiencing 0.3 pN force extracted from 15 tethered bead. The grey area is one standard deviation from the mean (black). The Allan deviation is the square root of the Allan variance, which determines the variance in position of the magnetic bead along the vertical axis between consecutive windows of duration τ. **(E)** The rotation extension of a tethered magnetic bead that is centered at zero turn and the measurement position (+3 turns) are beyond the plectonemic transition, i.e. in the constant torque regime. **(F)** Open complex dynamics for the tether calibrated in (E) with 150 mM KCl and 10 nM holo. **(G)** The rotation extension of a tethered magnetic bead performed in the same experiment as in (E), where the measurement position (+3 turns) precedes the plectonemic transition, i.e. at a lower torque than in (E). **(H)** Open complex dynamics for the tether calibrated in (G). The dashed red vertical lines indicate the detected transitions in (F) and (H) from the change-point analysis (**Materials and Methods**). **(I)** Histogram of the absolute difference in tether extensions between OS and CS, with mean values Δz_+ and Δz_- for positively (blue) and negatively (orange) supercoiled DNA, (N=107 and N=13, respectively). From this, we estimated the extend of DNA unwinding upon open complex formation $\Delta z_u = (\Delta z_+ + \Delta z_-)/2 = (57 \pm 5)\ nm$ (mean ± standard deviation), i.e. $(10 \pm 1)\ bp$, using one turn per 10.5 bp, and of bended DNA $\Delta z_b = (\Delta z_+ - \Delta z_-)/2 = (16 \pm 5)\ nm$ (mean ± standard deviation). **(J)** Schematic of the open complex formation experiments using a negatively supercoiled DNA molecule.

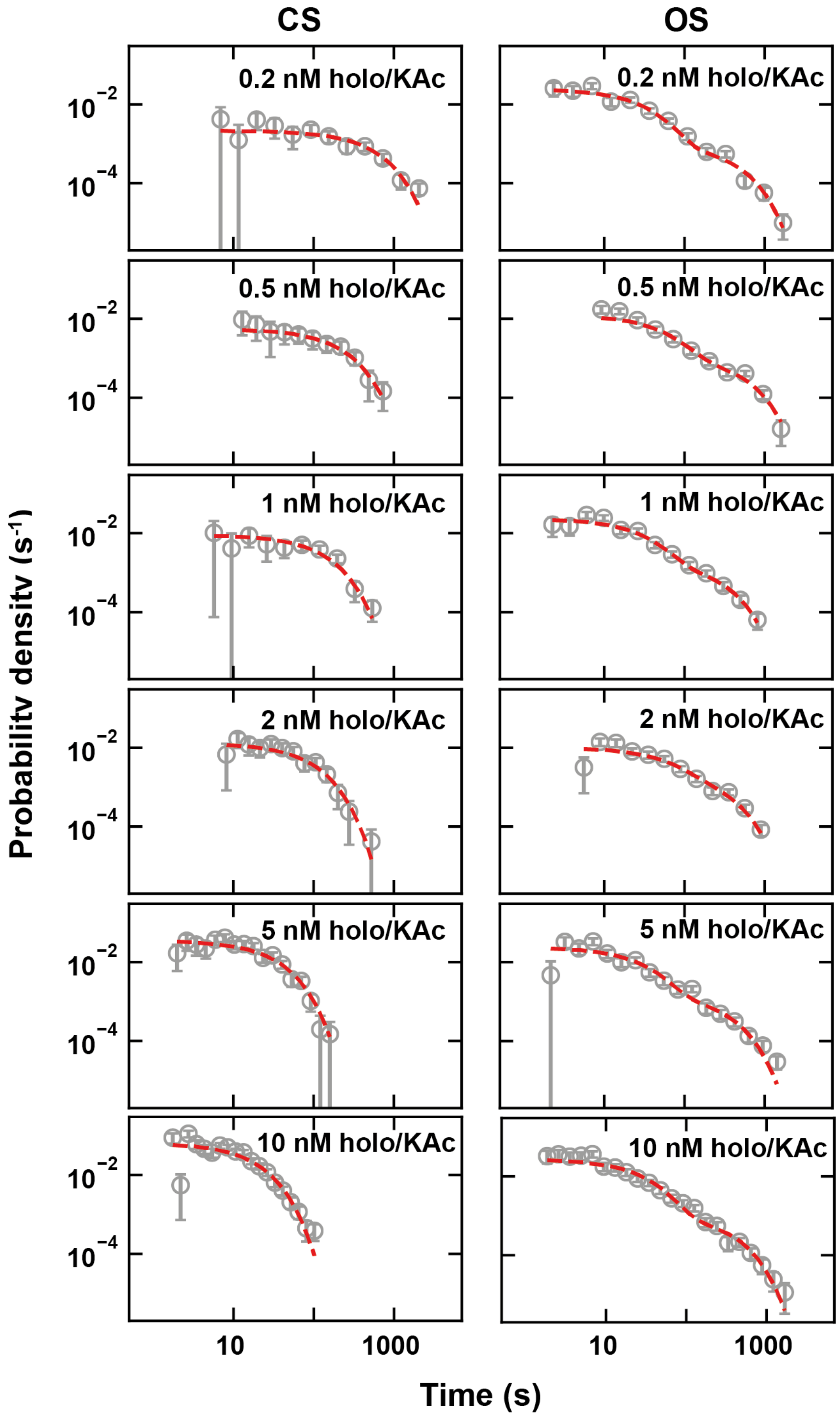

Supplementary Figure 2. CS and OS dwell times distribution as a function of holo concentration. Dwell time distributions of the open and closed states, i.e. OS and CS respectively, in 150 mM KAc at the holo concentration indicated in the panels. The dashed red line is either mono-exponential or bi-exponential MLE fit. Error bars are two standard deviations extracted from 1000 bootstraps.

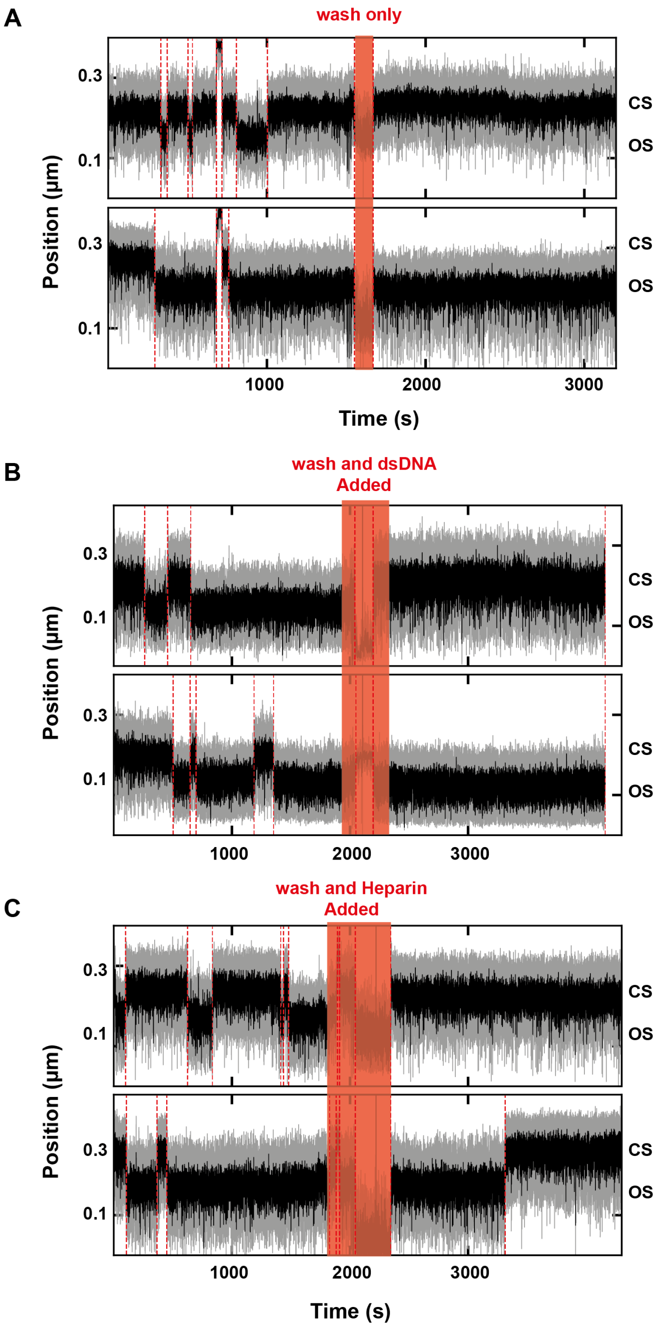

Supplementary Figure 3. The transition from open to closed state releases free the bound holo from the DNA. (A) Two representative traces showing the open complex dynamics for 0.5 nM holo and 150 mM of KGlu. The flow chamber is rinsed with 0.4 ml of reaction buffer at ~1800 s, and nothing is added afterwards. **(B)** Same experiment as in (A), and 0.2 ml of ~10 nM competing *lacCONS*+2 DNA promoter is then flushed after the flow chamber is rinsed with 0.2 ml of reaction buffer. **(C)** Same experiments as in (A), but 0.2 ml of 100 μg/ml heparin is added instead of competing DNA promoter after the flow chamber is rinsed with 0.2 ml of reaction buffer.

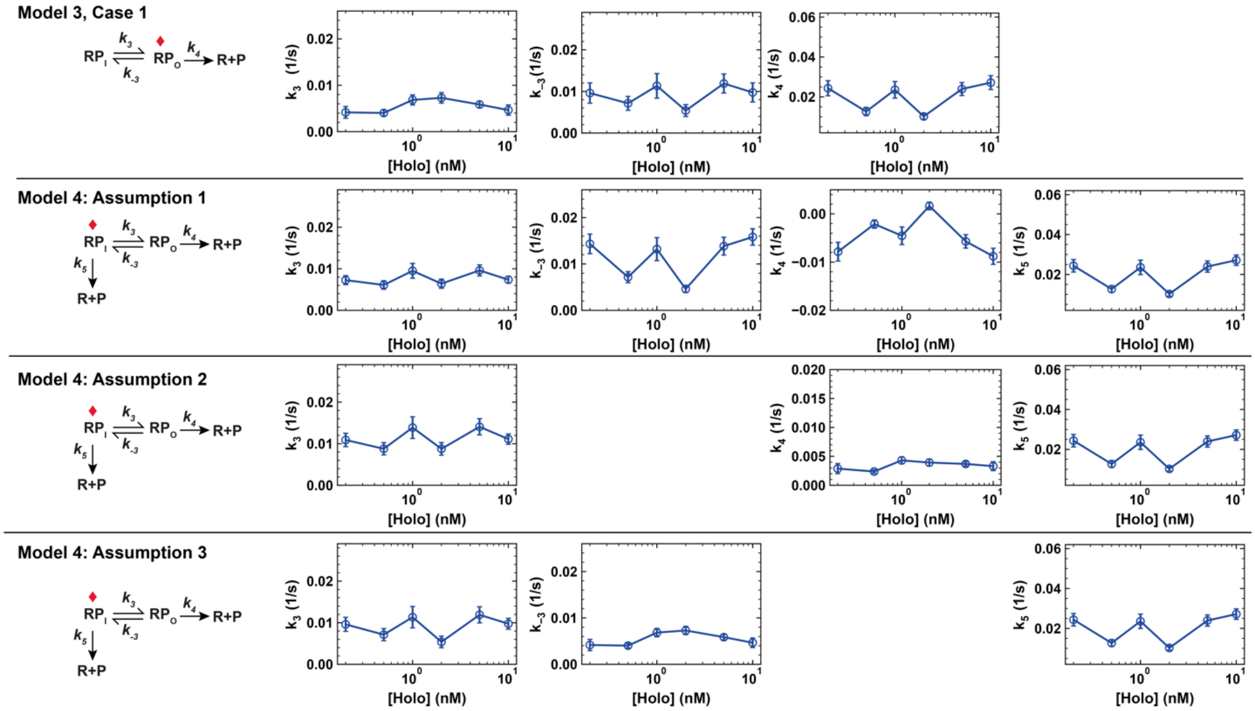

Supplementary Figure 4. Comparison of microscopic rate constants determined using the different models described in Table 1. Plots of the microscopic rate constants for Model 3 and Model 4. The error bars are one standard deviation extracted from error propagation as described in **Materials and Methods**.

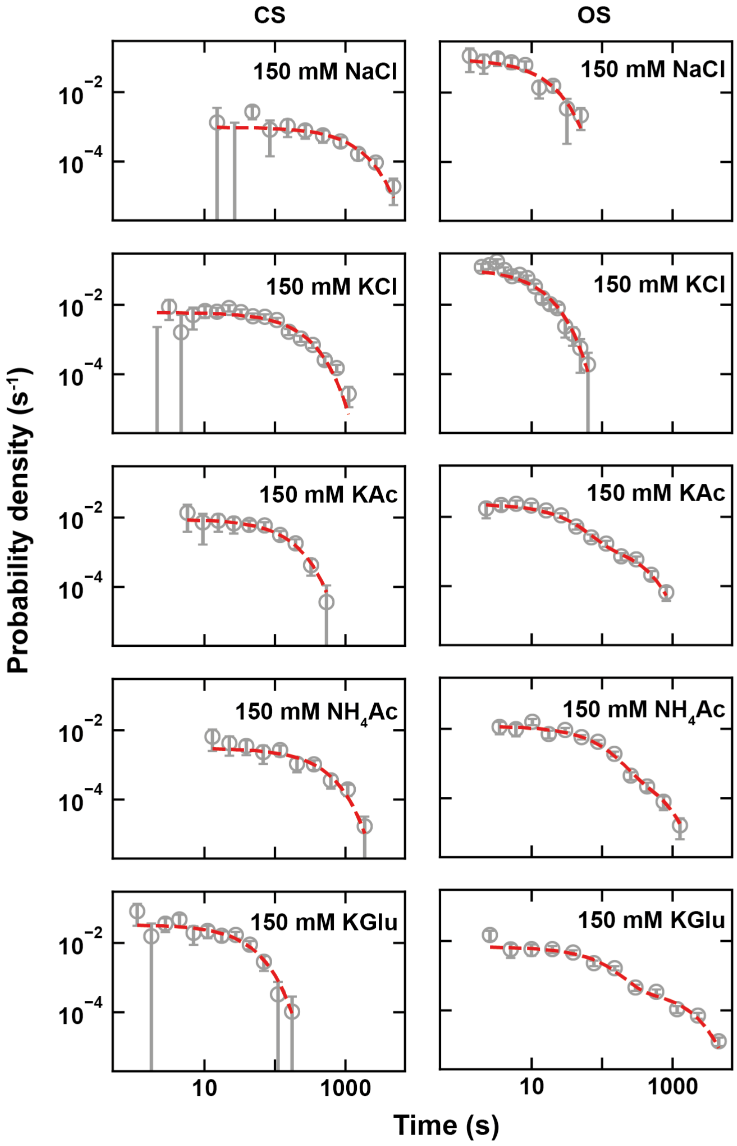

Supplementary Figure 5. CS and OS dwell times distribution as a function of monovalent salt types. Dwell time distributions of the open (OS) and closed (CS) states for different monovalent salts at the same concentration. 10 nM holo was used for KCl and NaCl, while 1 nM holo was used in the other conditions. The dashed red line is the either mono-exponential or bi-exponential MLE fit. Error bars are two standard deviations extracted from 1000 bootstraps.

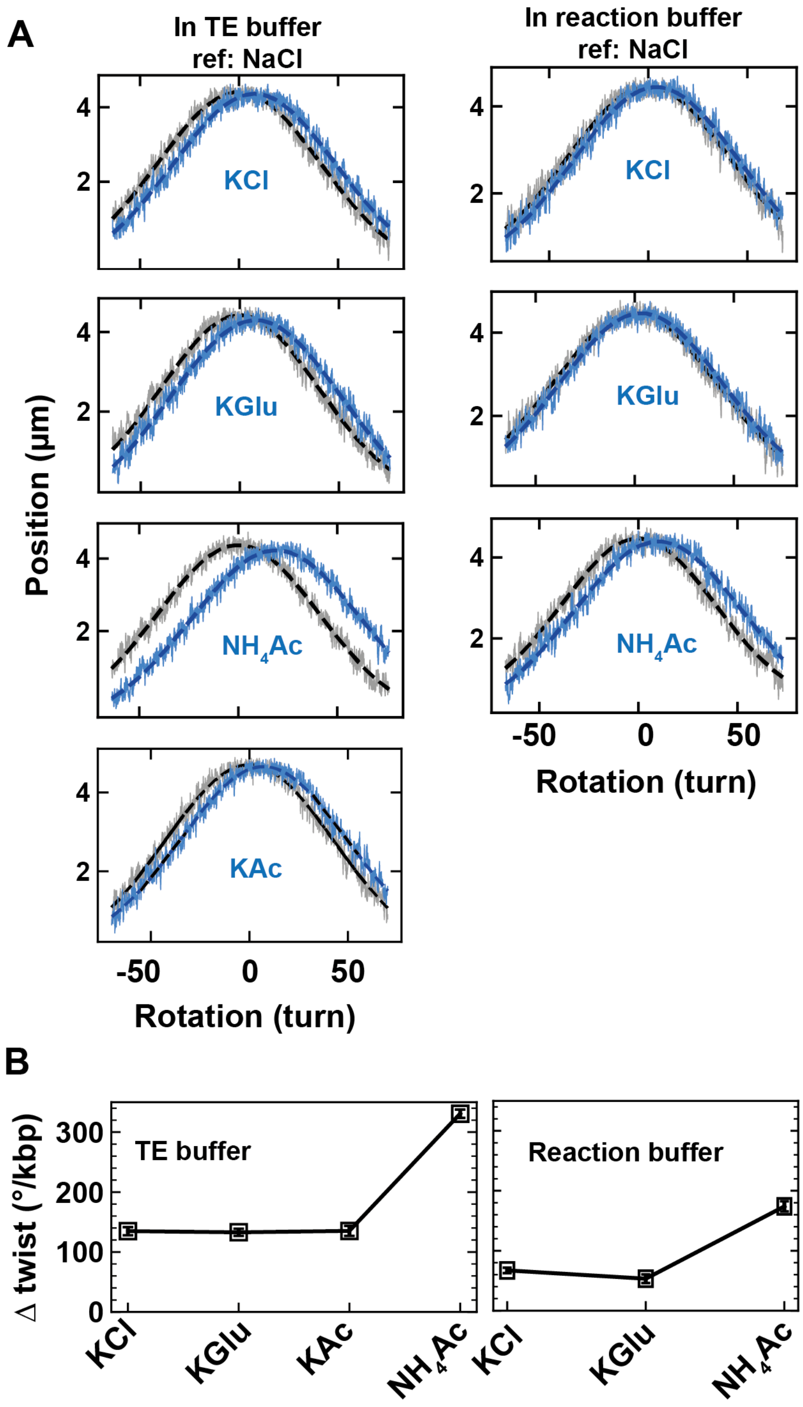

Supplementary Figure 6. The cation nature affects the DNA helical twist. (A) Extension as a function of DNA rotation (extension-rotation) for different monovalent salts at 150 mM concentration, either in TE buffer (left) or holo reaction buffer (right). The reference extension-rotation in NaCl is in black and the one measured in the indicated monovalent salt is in blue. The dashed lines are their respective Gaussian fit. **(B)**

Change in DNA helical twist (Δtwist) per degree of rotation and per kilo base pair in comparison of the reference measurement performed with 150 mM NaCl in either TE buffer (left) or reaction buffer (right).

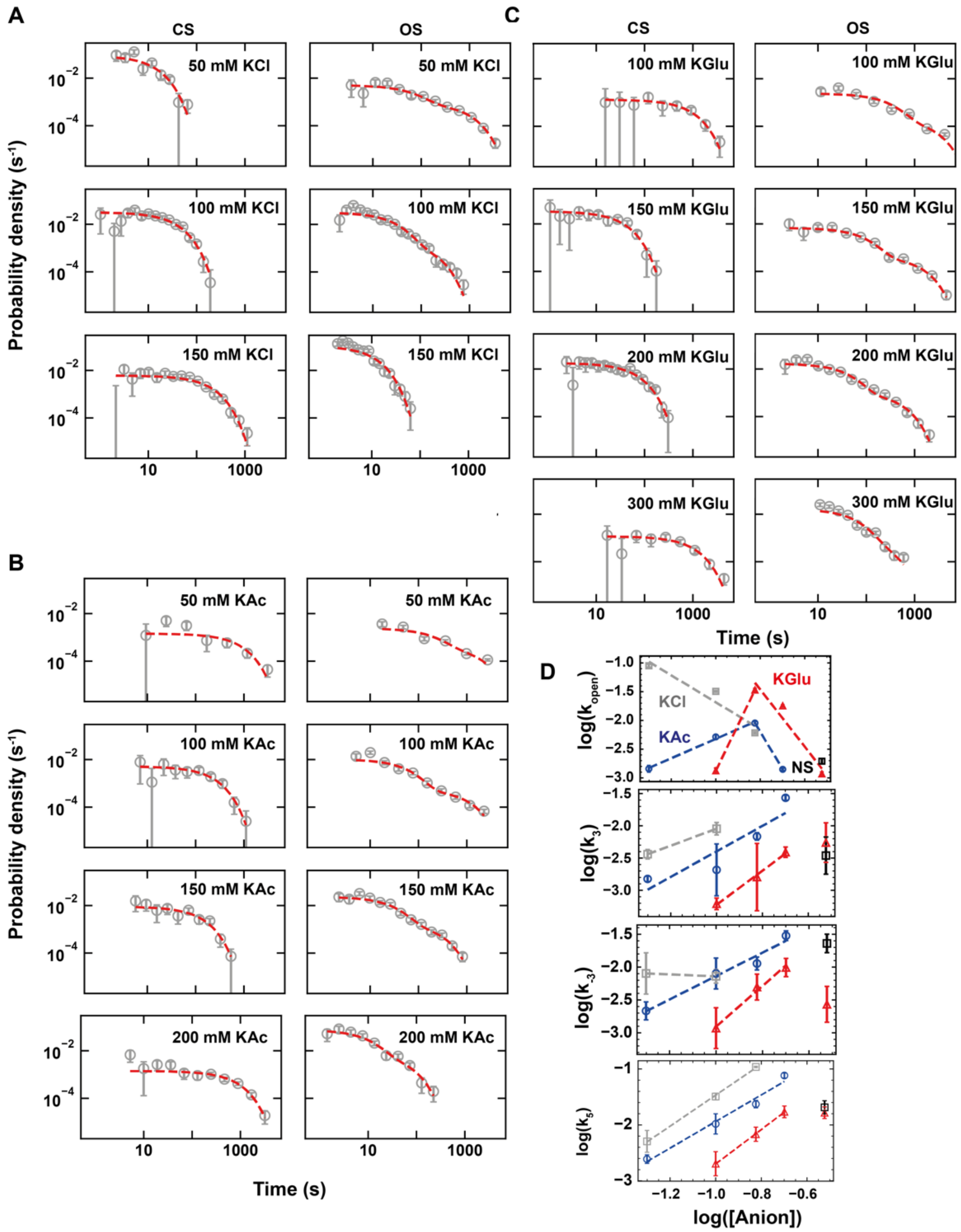

Supplementary Figure 7. CS and OS dwell times distribution as a function of anions concentration. Dwell time distributions of the open and closed states, i.e. OS and CS respectively, for **(A)** KCl, **(B)** KAc and **(C)** KGlu at the concentration indicated in the panels. 10 nM holo was used for KCl and 1 nM holo was used for other anions. The dashed red line is the either mono-exponential or bi-exponential MLE fit. Error bars are two standard deviations extracted from 1000 bootstraps. (**D**) Monovalent salt concentration dependence of k_{open}, k_3, k_{-3} and k_5 for KCl (grey), KAc (blue) and KGlu (red) with positively supercoiled DNA, and for KCl (black) with negatively supercoiled (NS) DNA, for activity traces acquired as in **Figure**

4 D-G represented in log10 of rate constants and salt concentrations. The solid lines connect the markers are linear fits and their respective slopes the Sk_i represented in **Table S4**. Error bars in (D, E, F, G) are the propagated errors from the one standard deviation error extracted from 1000 bootstraps.

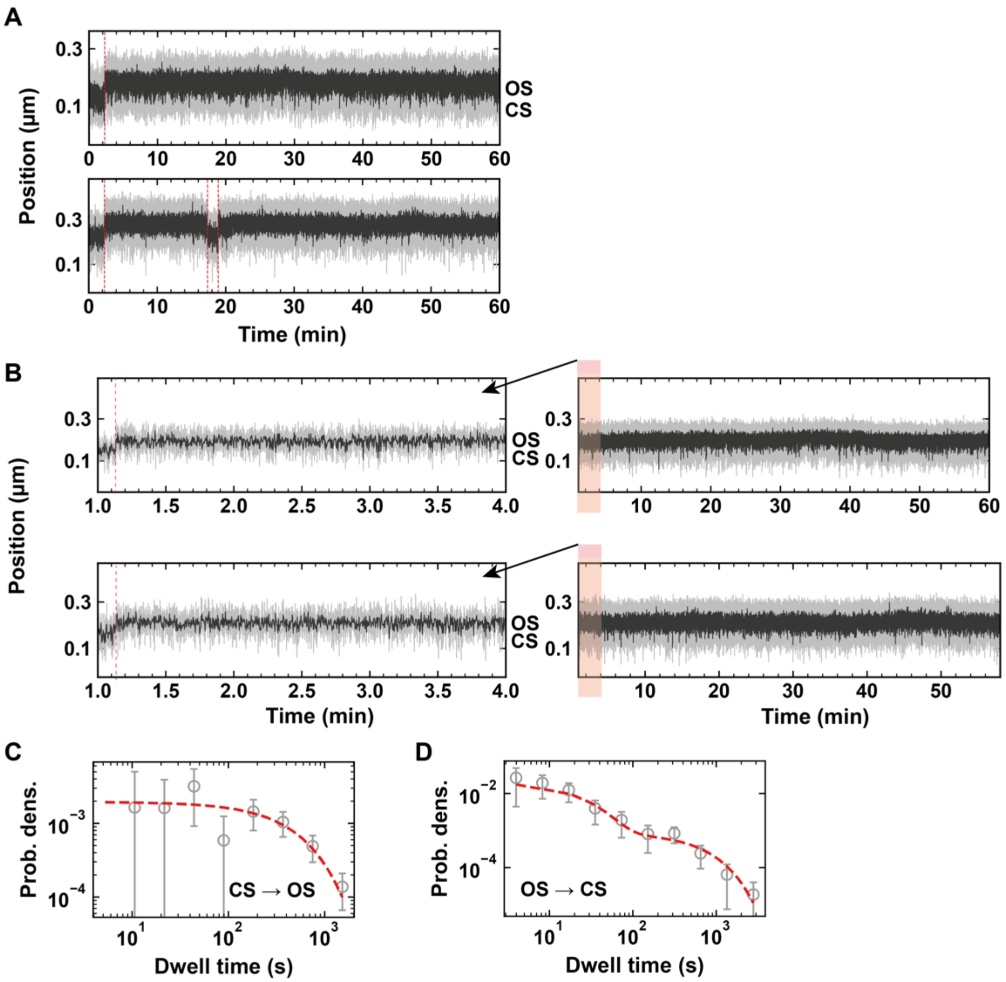

Supplementary Figure 8. Effect of negatively supercoiled promoter on the open complex dynamics. **(A)** Two representative traces showing the open complex dynamics for 5 nM holo at 200 mM of KCl. Transition from the CS to OS are indicated with RED-dashed lines. **(B)** Two representative traces showing the open complex dynamics for 0.5 nM holo at 300 mM of KGlu. Left panel is a zoom-in of the indicated part of the traces from the right panel. Transition from the CS to OS are indicated with RED-dashed lines. **(C, D)** Dwell time distributions of the CS and OS, respectively, from the experiments performed with 5 nM holo and 300 mM KCl. The dashed red line is either a single exponential (CS) or a double exponential (OS) MLE fit. Error bars are two standard deviations extracted from 1000 bootstraps. All experiments were performed at 34°C.

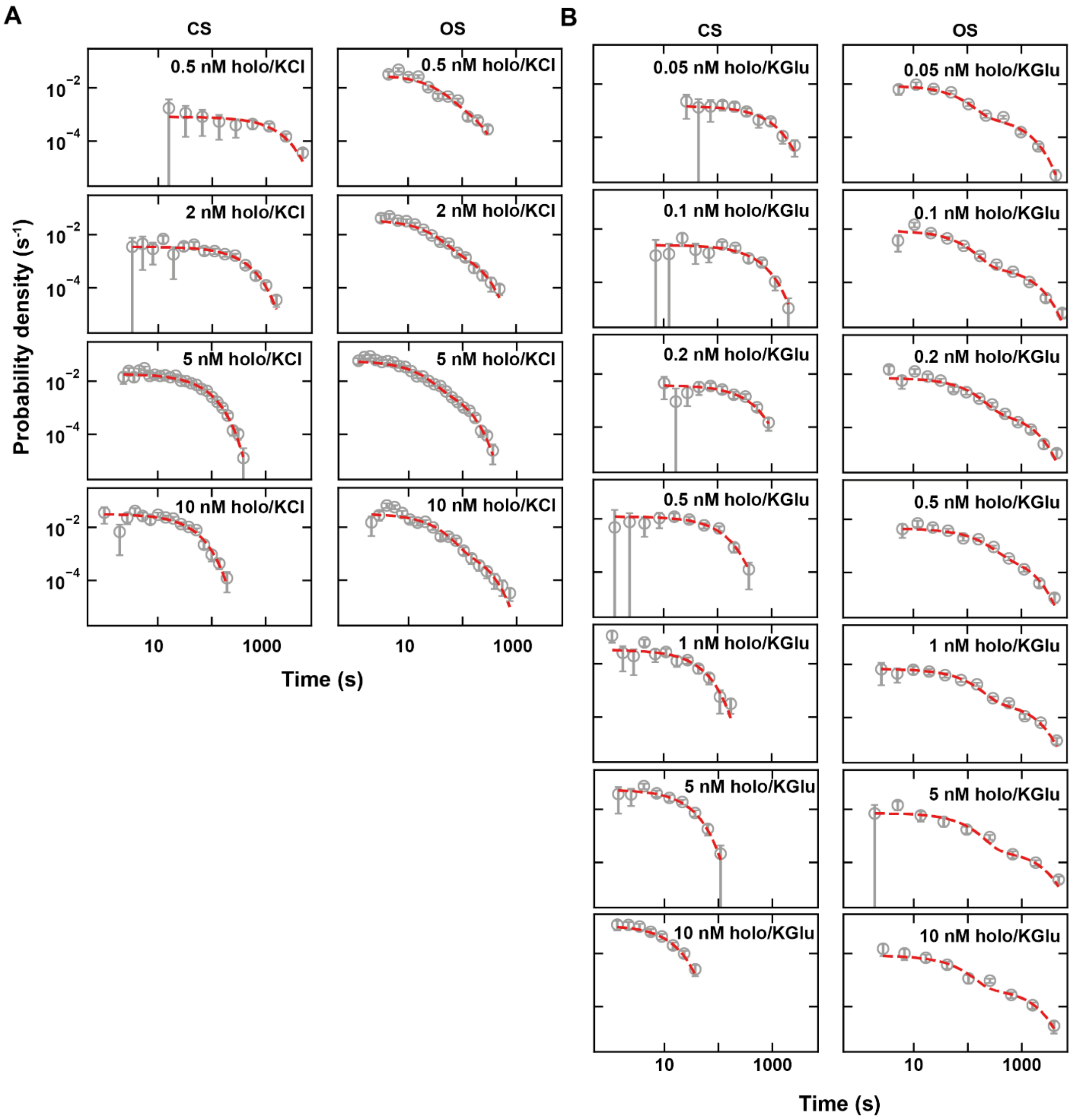

Supplementary Figure 9. CS and OS dwell times distribution as a function of holo concentration. Dwell time distributions of the open and closed states, i.e. OS and CS respectively, in (A) 100 mM KCl and (B) 150 mM KGlu at the holo concentration indicated in the panels. The dashed red line is either mono-exponential or bi-exponential MLE fit. Error bars are two standard deviations extracted from 1000 bootstraps.

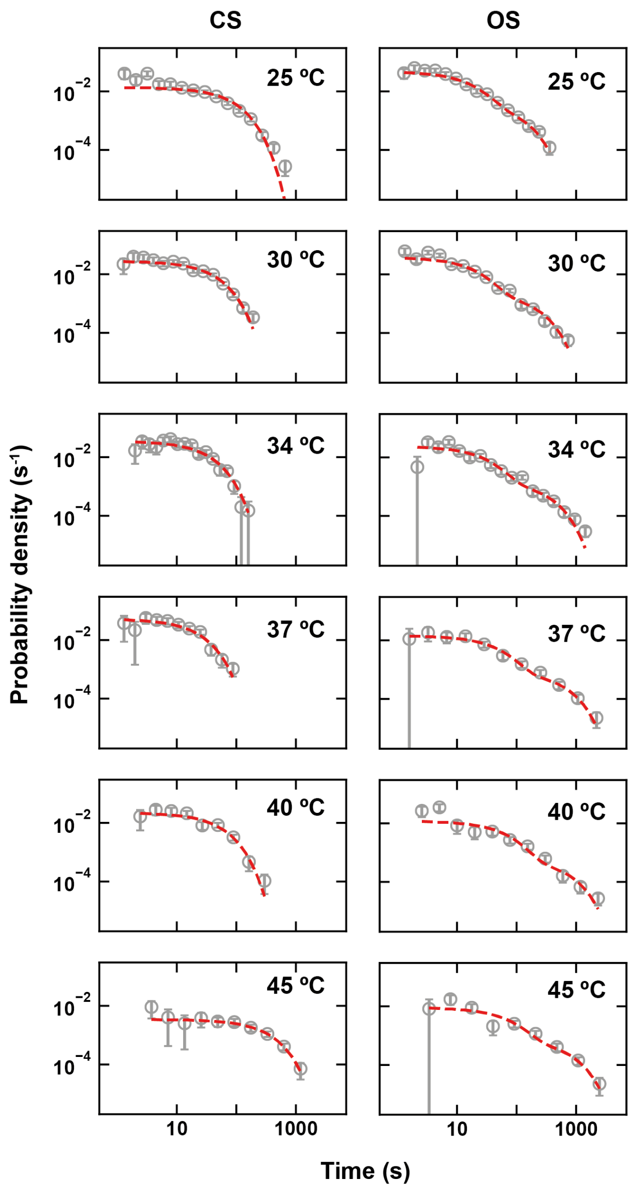

Supplementary Figure 10. Effect of temperature on the open complex dynamics. Dwell time distributions of the OS and CS at the indicated temperature, performed with in 150 mM KAc and 5 nM holo. The dashed red line is either a single exponential (CS) or a double exponential (OS) MLE fit. Error bars are two standard deviations extracted from 1000 bootstraps.

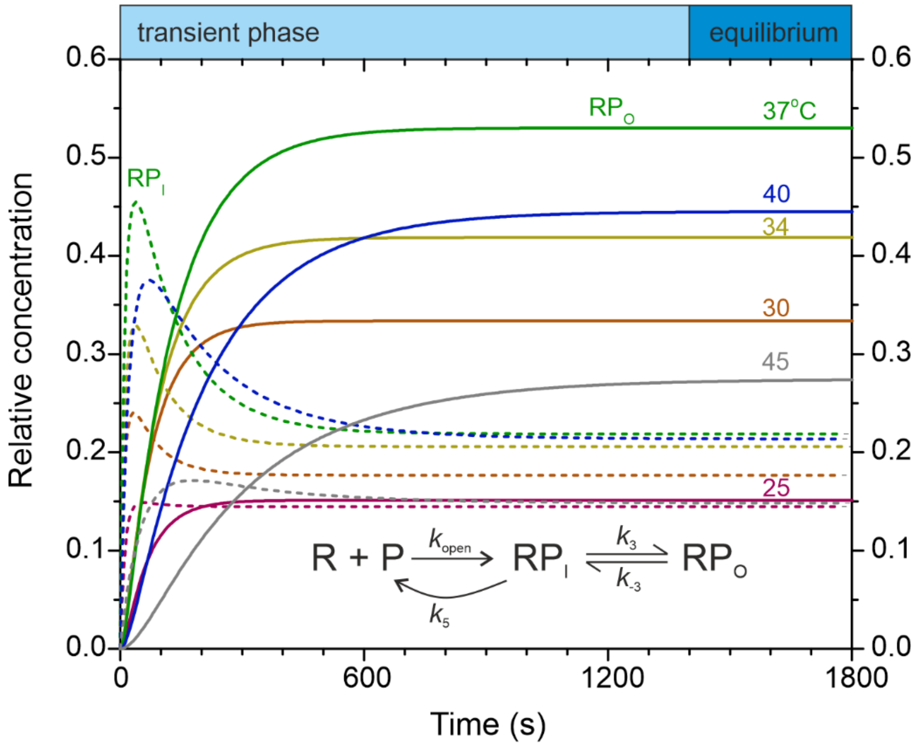

Supplementary Figure 11. Distribution of different holo–promoter complexes in a simulated bulk sample. The relative concentrations of RP_I (dashed line) and RP_O (solid line) complexes at different time-points and temperatures were simulated using KinTek Explorer chemical kinetics software (KinTek Corporation, PA, USA), the indicated model and the rate constants listed in Table S2. These rate constants were experimentally determined in the presence of 150 mM KAc and 5 nM holo. The simulation was started by the mixing of equimolar amounts of holo (R) and promoter at 0 s, and progressed via a transient phase to an equilibrium (reached by 1400 s). The relative concentration of free promoter is not shown but can be calculated by $[P_{free}] = 1 - [RP_I] - [RP_O]$. k_{open} is a combined rate constant for the holo binding to the promoter and isomerization of the initial RP_C complex to RP_I. k_5 is the rate constant for holo dissociation from RP_I. k_3 and k_{-3} are the forward and reverse rate constants for RP_I to RP_O isomerization, respectively.

Table S1: Experimental conditions and their parameters directly obtained from MLE fits

Experiment Conditions			Supercoil sign	T (˚C)	Used in figures	Number of traces				Close state dwell time			Open state dwell time						
Salt	[Salt] (mM)	[Holo] (nM)				Total	Active	Active frac.	95% CI	Dwell times	k_open (1/s)	SD (1/s)	Dwell times	k_(1/s)	SD (1/s)	k+ (1/s)	SD (1/s)	p+ (1/s)	SD (1/s)
	50				3, S4	77	75	0.97	0.04	236	0.0894	0.0055	258	0.016	0.008	0.0012	0.0002	0.73	0.08
	100	10			3, 4, S4, S5	37	34	0.92	0.09	926	0.0320	0.0010	922	0.041	0.006	0.0070	0.0016	0.27	0.08
	150				2, 3, S1, S2, S4	39	38	0.97	0.05	1031	0.0061	0.0002	1030	0.109	0.003	-	-	-	-
KCl		10	(+)	34	3, 4, S4, S5	37	34	0.92	0.09	926	0.0320	0.0010	922	0.041	0.006	0.0070	0.0016	0.27	0.08
	100	5			4, S5	54	53	0.98	0.04	3639	0.0187	0.0003	3641	0.086	0.012	0.0166	0.0008	0.40	0.03
		2			4, S5	59	56	0.95	0.06	602	0.0035	0.0002	579	0.053	0.008	0.0089	0.0015	0.37	0.07
		0.5			4, S5	48	41	0.85	0.10	162	0.0008	0.0001	137	0.044	0.009	0.0104	0.0019	0.38	0.11
	300	5	(-)	34	4, S7	23	20	0.87	0.14	83	0.0020	0.0002	85	0.045	0.013	0.0016	0.0011	0.57	0.07
	50				3, S4	60	57	0.95	0.06	101	0.0014	0.0002	78	0.005	0.001	0.0007	0.0001	0.63	0.08
	100	1			3, S4	36	36	1.00	0.00	133	0.0052	0.0005	125	0.019	0.008	0.0011	0.0011	0.49	0.09
	150				2, 3, S2, S4	29	27	0.93	0.09	207	0.0090	0.0006	196	0.037	0.006	0.0043	0.0006	0.42	0.06
	200				3, S4	61	59	0.97	0.04	453	0.0014	0.0001	418	0.116	0.013	0.0179	0.0018	0.40	0.05
KAc		10	(+)	34	1, 4, S5	57	56	0.98	0.04	1479	0.0649	0.0017	1455	0.038	0.004	0.0033	0.0008	0.32	0.07
		5			4, 5, 6, S5, S7	51	49	0.96	0.05	770	0.0353	0.0012	781	0.038	0.005	0.0037	0.0004	0.41	0.05
	150	2			4, S5	46	44	0.96	0.06	286	0.0129	0.0007	306	0.019	0.003	0.0039	0.0006	0.58	0.06
		1			2, 3, S2, S4	29	27	0.93	0.09	207	0.0090	0.0006	196	0.037	0.006	0.0043	0.0006	0.42	0.06
		0.5			4, S5	38	37	0.97	0.05	164	0.0056	0.0004	176	0.021	0.003	0.0024	0.0003	0.46	0.06
		0.2			4, S5	46	43	0.94	0.07	273	0.0026	0.0001	260	0.035	0.005	0.0029	0.0009	0.34	0.07
	100				3, S4	50	48	0.96	0.05	102	0.0013	0.0001	104	0.003	0.002	0.0004	0.0001	0.46	0.09
	150	1			2, 3, S2, S4	47	45	0.96	0.06	243	0.0338	0.0023	230	0.013	0.004	0.0009	0.0011	0.49	0.07
	200				3, S4	49	49	1.00	0.00	481	0.0179	0.0008	466	0.029	0.007	0.0023	0.0005	0.44	0.07
	300				3, S4	43	40	0.93	0.08	206	0.0012	0.0001	193	0.020	0.005	0.0045	0.0034	0.24	0.15
		0.05			4, S5	53	52	0.98	0.04	168	0.0015	0.0001	158	0.018	0.005	0.0012	0.0008	0.53	0.09
KGlu		0.1	(+)	34	4, S5	65	64	0.99	0.03	251	0.0025	0.0002	245	0.016	0.004	0.0009	0.0022	0.48	0.06
		0.2			4, S5	49	47	0.96	0.06	268	0.0038	0.0002	267	0.012	0.006	0.0010	0.0008	0.42	0.08
	150	0.5			2, 4S, S6	64	61	0.95	0.06	272	0.0124	0.0008	263	0.008	0.003	0.0011	0.0004	0.47	0.10
		1			2, 3, S2, S4	47	45	0.96	0.06	243	0.0338	0.0023	230	0.013	0.004	0.0009	0.0011	0.49	0.07
		5			4, S5	56	54	0.96	0.05	322	0.0554	0.0035	335	0.013	0.003	0.0007	0.0001	0.46	0.04
		10			4, S5	58	56	0.97	0.04	318	0.1161	0.0058	335	0.018	0.004	0.0009	0.0001	0.57	0.04
NaCl	150	10	(+)	34	2, S2	53	13	0.25	0.12	66	0.0010	0.0001	54	0.092	0.008	-	-	-	-
NH4Ac	150	1	(+)	34	2, S2	45	43	0.96	0.06	179	0.0030	0.0002	169	0.015	0.004	0.0029	0.0026	0.25	0.14
				25	4, 5, 6, S5, S7	41	40	0.98	0.04	807	0.0137	0.0005	1448	0.068	0.013	0.0106	0.0007	0.36	0.04
				30	5, 6, S7	44	47	0.94	0.07	1587	0.0280	0.0008	1647	0.058	0.006	0.0059	0.0011	0.38	0.04
KAc	150	5	(+)	34	5, 6, S7	51	49	0.96	0.05	770	0.0350	0.0012	781	0.038	0.005	0.0037	0.0004	0.41	0.05
				37	5, 6, S7	54	53	0.98	0.04	393	0.0520	0.0025	409	0.024	0.004	0.0020	0.0002	0.43	0.05
				40	5, 6, S7	46	45	0.98	0.04	383	0.0220	0.0013	380	0.018	0.005	0.0017	0.0003	0.35	0.07
				45	5, 6, S7	49	49	1.00	0.00	284	0.0040	0.0002	278	0.016	0.004	0.0016	0.0002	0.46	0.05

Table S2: Experimental conditions and their related parameters extracted from MLE procedures. The rate constants k_3, k_{-3} and k_5 were extracted according to Model 4, Assumption 3.

Experiment Conditions			Supercoil sign	T ('C)	Used in figures	Number of traces				Close state dwell time			Open state dwell time						
Salt	[Salt] (mM)	[Holo] (nM)				Total	Active	active frac.	95% CI	Dwell times	k_open (1/s)	SD (1/s)	Dwell times	k3 (1/s)	SD (1/s)	k-3 (1/s)	SD (1/s)	k5 (1/s)	SD (1/s)
	50				3, S4	77	75	0.97	0.04	236	0.0894	0.0055	258	0.008	0.006	0.0036	0.0010	0.005	0.003
	100	10			3, 4, S4, S5	37	34	0.92	0.09	926	0.0320	0.0010	922	0.007	0.003	0.0090	0.0022	0.032	0.005
	150				2, 3, S1, S2, S4	39	38	0.97	0.05	1031	0.0061	0.0002	1030	-	-	-	-	0.109	0.003
KCl		10	(+)	34	3, 4, S4, S5	37	34	0.92	0.09	926	0.0320	0.0010	922	0.007	0.003	0.0090	0.0022	0.032	0.005
	100	5			4, S5	54	53	0.98	0.04	3639	0.0187	0.0003	3641	0.020	0.004	0.0244	0.0014	0.059	0.007
		2			4, S5	59	56	0.95	0.06	602	0.0035	0.0002	579	0.012	0.004	0.0129	0.0023	0.037	0.006
		0.5			4, S5	48	41	0.85	0.1	162	0.0008	0.0001	137	0.008	0.004	0.0146	0.0029	0.031	0.007
	300	5	(-)		4, S7	23	20	0.87	0.14	83	0.0020	0.0002	85	0.023	0.007	0.0035	0.0023	0.021	0.006
	50				3, S4	60	57	0.95	0.06	101	0.0014	0.0002	78	0.002	0.001	0.0015	0.0003	0.002	0.001
	100	1			3, S4	36	36	1	0	133	0.0052	0.0005	125	0.008	0.005	0.0021	0.0019	0.010	0.005
	150				2, 3, S2, S4	29	27	0.93	0.09	207	0.0090	0.0006	196	0.011	0.003	0.0068	0.0011	0.024	0.004
	200				3, S4	61	59	0.97	0.04	453	0.0014	0.0001	418	0.030	0.006	0.0272	0.0031	0.076	0.009
KAc		10	(+)	34	1, 4, S5	57	56	0.98	0.04	1479	0.0649	0.0017	1455	0.010	0.002	0.0047	0.0011	0.027	0.003
		5			4, 5, 6, S5, S7	51	49	0.96	0.05	770	0.0353	0.0012	781	0.012	0.002	0.0059	0.0007	0.024	0.003
	150	2			4, S5	46	44	0.96	0.06	286	0.0129	0.0007	306	0.005	0.001	0.0073	0.0011	0.010	0.001
		1			2, 3, S2, S4	29	27	0.93	0.09	207	0.0090	0.0006	196	0.011	0.003	0.0068	0.0011	0.024	0.004
		0.5			4, S5	38	37	0.97	0.05	164	0.0056	0.0004	176	0.007	0.002	0.0040	0.0006	0.013	0.002
		0.2			4, S5	46	43	0.94	0.07	273	0.0026	0.0001	260	0.010	0.002	0.0042	0.0013	0.024	0.004
	100				3, S4	50	48	0.96	0.05	102	0.0013	0.0001	104	0.001	0.001	0.0006	0.0002	0.002	0.001
	150	1			2, 3, S2, S4	47	45	0.96	0.06	243	0.0338	0.0023	230	0.005	0.002	0.0016	0.0019	0.007	0.002
	200				3, S4	49	49	1	0	481	0.0179	0.0008	466	0.010	0.003	0.0039	0.0008	0.017	0.005
	300				3, S4	43	40	0.93	0.08	206	0.0012	0.0001	193	0.003	0.002	0.0055	0.0040	0.017	0.005
		0.05			4, S5	53	52	0.98	0.04	168	0.0015	0.0001	158	0.008	0.003	0.0023	0.0016	0.009	0.003
KGlu		0.1	(+)	34	4, S5	65	64	0.99	0.03	251	0.0025	0.0002	245	0.007	0.003	0.0016	0.0038	0.009	0.003
		0.2			4, S5	49	47	0.96	0.06	268	0.0038	0.0002	267	0.004	0.003	0.0016	0.0012	0.007	0.004
	150	0.5			2, 4S, S6	64	61	0.95	0.06	272	0.0124	0.0008	263	0.002	0.002	0.0018	0.0007	0.005	0.002
		1			2, 3, S2, S4	47	45	0.96	0.06	243	0.0338	0.0023	230	0.005	0.002	0.0016	0.0019	0.007	0.002
		5			4, S5	56	54	0.96	0.05	322	0.0554	0.0035	335	0.005	0.001	0.0012	0.0002	0.007	0.002
		10			4, S5	58	56	0.97	0.04	318	0.1161	0.0058	335	0.009	0.002	0.0020	0.0003	0.008	0.002
NaCl	150	10	(+)	34	2, S2	53	13	0.25	0.12	66	0.0010	0.0001	54	-	-	-	-	0.092	0.008
NH4Ac	150	1	(+)	34	2, S2	45	43	0.96	0.06	179	0.0030	0.0002	169	0.002	0.002	0.0036	0.0031	0.012	0.004
				25	4, 5, 6, S5, S7	41	40	0.98	0.04	807	0.0137	0.0005	1448	0.016	0.005	0.0153	0.0012	0.047	0.008
				30	5, 6, S7	44	47	0.94	0.07	1587	0.0280	0.0008	1647	0.017	0.003	0.0090	0.0016	0.038	0.004
KAc	150	5	(+)	34	5, 6, S7	51	49	0.96	0.05	770	0.0350	0.0012	781	0.012	0.002	0.0059	0.0007	0.024	0.003
				37	5, 6, S7	54	53	0.98	0.04	393	0.0520	0.0025	409	0.008	0.002	0.0033	0.0004	0.015	0.003
				40	5, 6, S7	46	45	0.98	0.04	383	0.0220	0.0013	380	0.005	0.002	0.0024	0.0005	0.012	0.003
				45	5, 6, S7	49	49	1	0	284	0.0040	0.0002	278	0.005	0.002	0.0027	0.0004	0.009	0.002

Table S3: Equilibrium constants determined from holo concentration dependent experiment fitting the data with **Equation 1**.

Salt	K_1 (μM^{-1})	k_2 (s^{-1})
100 mM KCl	0.95 ± 4.96	3.0 ± 15.7
150 mM KAc	60.1 ± 4.8	0.17 ± 0.01
150 mM KGlu	6.5 ± 6.4	2.4 ± 2.3

Table S4: Sk_i determined from the slopes of the least-mean-squares linear fits of the salt concentration dependent experiments reported in **Supplementary Figure 7D**.

Sk_i	KCl [salt range]	KAc [salt range]	KGlu [salt range]
Sk_{open}	-2.3 [50-150 mM]	1.7 [50-150 mM]<br>-6.5 [150-200 mM]	8 [100-150 mM]<br>-5 [150-300 mM]
Sk_3	-0.2 [50-100 mM]	1.8 [50-200 mM]	3.1 [100-200 mM]
Sk_{-3}	1.3 [50-100 mM]	2 [50-200 mM]	2.6 [100-200 mM]
Sk_5	2.8 [50-150 mM]	2.4 [50-200 mM]	3.1 [100-200 mM]

References

1. Floyd, D.L., Harrison, S.C. and van Oijen, A.M. (2010) Analysis of kinetic intermediates in single-particle dwell-time distributions. *Biophysical journal*, **99**, 360-366.
2. Xie, S.N. (2001) Single-molecule approach to enzymology. *Single Mol*, **2**, 229-236.
