# Supplementary material for: Quantitative parameters of bacterial RNA polymerase open-complex formation, stabilization and disruption on a consensus promoter
Source: Nucleic Acids Res. 2022 Jul 12;50(13):7511–28. doi: 10.1093/nar/gkac560 (PMC9303404; doi:10.1093/nar/gkac560)
Supplement: gkac560_Supplemental_Files [file gkac560_supplemental_files.zip › Caption for Supplementary Data 1-2.docx]

**Supplementary data 1**: Sequence of the 20.6 kbp DNA construct used in the experiments investigating the monovalent salt nature dependence of the DNA twist.

**Supplementary data 2:** Sequence of the 1.4 kbp DNA construct used in the bacterial RNA polymerase open complex dynamic experiments
