# Supplementary material for: Quantitative parameters of bacterial RNA polymerase open-complex formation, stabilization and disruption on a consensus promoter
Source: Nucleic Acids Res. 2022 Jul 12;50(13):7511–28. doi: 10.1093/nar/gkac560 (PMC9303404; doi:10.1093/nar/gkac560)
Supplement: gkac560_Supplemental_Files [file gkac560_supplemental_files.zip › Supplementary Data 1.rtf]

>20.66kb_tether
TCGAGATGGTGCATCCCTCAAAACGAGGGAAAATCCCCTAAAACGAGGGATAAAACATCCCTCAAATTGGGGGATTGCTATCCCTCAAAACAGGGGGACACAAAAGACACTATTACAAAAGAAAAAAGAAAAGATTATTCGTCAGAGAATTCTGGCGAATCCTCTGACCAGCCAGAAAACGACCTTTCTGTGGTGAAACCGGATGCTGCAATTCAGAGCGGCAGCAAGTGGGGGACAGCAGAAGACCTGACCGCCGCAGAGTGGATGTTTGACATGGTGAAGACTATCGCACCATCAGCCAGAAAACCGAATTTTGCTGGGTGGGCTAACGATATCCGCCTGATGCGTGAACGTGACGGACGTAACCACCGCGACATGTGTGTGCTGTTCCGCTGGGCATGCCAGGACAACTTCTGGTCCGGTAACGTGCTGAGCCCGGCCAAACTCCGCGATAAGTGGACCCAACTCGAAATCAACCGTAACAAGCAACAGGCAGGCGTGACAGCCAGCAAACCAAAACTCGACCTGACAAACACAGACTGGATTTACGGGGTGGATCTATGAAAAACATCGCCGCACAGATGGTTAACTTTGACCGTGAGCAGATGCGTCGGATCGCCAACAACATGCCGGAACAGTACGACGAAAAGCCGCAGGTACAGCAGGTAGCGCAGATCATCAACGGTGTGTTCAGCCAGTTACTGGCAACTTTCCCGGCGAGCCTGGCTAACCGTGACCAGAACGAAGTGAACGAAATCCGTCGCCAGTGGGTTCTGGCTTTTCGGGAAAACGGGATCACCACGATGGAACAGGTTAACGCAGGAATGCGCGTAGCCCGTCGGCAGAATCGACCATTTCTGCCATCACCCGGGCAGTTTGTTGCATGGTGCCGGGAAGAAGCATCCGTTACCGCCGGACTGCCAAACGTCAGCGAGCTGGTTGATATGGTTTACGAGTATTGCCGGAAGCGAGGCCTGTATCCGGATGCGGAGTCTTATCCGTGGAAATCAAACGCGCACTACTGGCTGGTTACCAACCTGTATCAGAACATGCGGGCCAATGCGCTTACTGATGCGGAATTACGCCGTAAGGCCGCAGATGAGCTTGTCCATATGACTGCGAGAATTAACCGTGGTGAGGCGATCCCTGAACCAGTAAAACAACTTCCTGTCATGGGCGGTAGACCTCTAAATCGTGCACAGGCTCTGGCGAAGATCGCAGAAATCAAAGCTAAGTTCGGACTGAAAGGAGCAAGTGTATGACGGGCAAAGAGGCAATTATTCATTACCTGGGGACGCATAATAGCTTCTGTGCGCCGGACGTTGCCGCGCTAACAGGCGCAACAGTAACCAGCATAAATCAGGCCGCGGCTAAAATGGCACGGGCAGGTCTTCTGGTTATCGAAGGTAAGGTCTGGCGAACGGTGTATTACCGGTTTGCTACCAGGGAAGAACGGGAAGGAAAGATGAGCACGAACCTGGTTTTTAAGGAGTGTCGCCAGAGTGCCGCGATGAAACGGGTATTGGCGGTATATGGAGTTAAAAGATGACCATCTACATTACTGAGCTAATAACAGGCCTGCTGGTAATCGCAGGCCTTTTTATTTGGGGGAGAGGGAAGTCATGAAAAAACTAACCTTTGAAATTCGATCTCCAGCACATCAGCAAAACGCTATTCACGCAGTACAGCAAATCCTTCCAGACCCAACCAAACCAATCGTAGTAACCATTCAGGAACGCAACCGCAGCTTAGACCAAAACAGGAAGCTATGGGCCTGCTTAGGTGACGTCTCTCGTCAGGTTGAATGGCATGGTCGCTGGCTGGATGCAGAAAGCTGGAAGTGTGTGTTTACCGCAGCATTAAAGCAGCAGGATGTTGTTCCTAACCTTGCCGGGAATGGCTTTGTGGTAATAGGCCAGTCAACCAGCAGGATGCGTGTAGGCGAATTTGCGGAGCTATTAGAGCTTATACAGGCATTCGGTACAGAGCGTGGCGTTAAGTGGTCAGACGAAGCGAGACTGGCTCTGGAGTGGAAAGCGAGATGGGGAGACAGGGCTGCATGATAAATGTCGTTAGTTTCTCCGGTGGCAGGACGTCAGCATATTTGCTCTGGCTAATGGAGCAAAAGCGACGGGCAGGTAAAGACGTGCATTACGTTTTCATGGATACAGGTTGTGAACATCCAATGACATATCGGTTTGTCAGGGAAGTTGTGAAGTTCTGGGATATACCGCTCACCGTATTGCAGGTTGATATCAACCCGGAGCTTGGACAGCCAAATGGTTATACGGTATGGGAACCAAAGGATATTCAGACGCGAATGCCTGTTCTGAAGCCATTTATCGATATGGTAAAGAAATATGGCACTCCATACGTCGGCGGCGCGTTCTGCACTGACAGATTAAAACTCGTTCCCTTCACCAAATACTGTGATGACCATTTCGGGCGAGGGAATTACACCACGTGGATTGGCATCAGAGCTGATGAACCGAAGCGGCTAAAGCCAAAGCCTGGAATCAGATATCTTGCTGAACTGTCAGACTTTGAGAAGGAAGATATCCTCGCATGGTGGAAGCAACAACCATTCGATTTGCAAATACCGGAACATCTCGGTAACTGCATATTCTGCATTAAAAAATCAACGCAAAAAATCGGACTTGCCTGCAAAGATGAGGAGGGATTGCAGCGTGTTTTTAATGAGGTCATCACGGGATCCCATGTGCGTGACGGACATCGGGAAACGCCAAAGGAGATTATGTACCGAGGAAGAATGTCGCTGGACGGTATCGCGAAAATGTATTCAGAAAATGATTATCAAGCCCTGTATCAGGACATGGTACGAGCTAAAAGATTCGATACCGGCTCTTGTTCTGAGTCATGCGAAATATTTGGAGGGCAGCTTGATTTCGACTTCGGGAGGGAAGCTGCATGATGCGATGTTATCGGTGCGGTGAATGCAAAGAAGATAACCGCTTCCGACCAAATCAACCTTACTGGAATCGATGGTGTCTCCGGTGTGAAAGAACACCAACAGGGGTGTTACCACTACCGCAGGAAAAGGAGGACGTGTGGCGAGACAGCGACGAAGTATCACCGACATAATCTGCGAAAACTGCAAATACCTTCCAACGAAACGCACCAGAAATAAACCCAAGCCAATCCCAAAAGAATCTGACGTAAAAACCTTCAACTACACGGCTCACCTGTGGGATATCCGGTGGCTAAGACGTCGTGCGAGGAAAACAAGGTGATTGACCAAAATCGAAGTTACGAACAAGAAAGCGTCGAGCGAGCTTTAACGTGCGCTAACTGCGGTCAGAAGCTGCATGTGCTGGAAGTTCACGTGTGTGAGCACTGCTGCGCAGAACTGATGAGCGATCCGAATAGCTCGATGCACGAGGAAGAAGATGATGGCTAAACCAGCGCGAAGACGATGTAAAAACGATGAATGCCGGGAATGGTTTCACCCTGCATTCGCTAATCAGTGGTGGTGCTCTCCAGAGTGTGGAACCAAGATAGCACTCGAACGACGAAGTAAAGAACGCGAAAAAGCGGAAAAAGCAGCAGAGAAGAAACGACGACGAGAGGAGCAGAAACAGAAAGATAAACTTAAGATTCGAAAACTCGCCTTAAAGCCCCGCAGTTACTGGATTAAACAAGCCCAACAAGCCGTAAACGCCTTCATCAGAGAAAGAGACCGCGACTTACCATGTATCTCGTGCGGAACGCTCACGTCTGCTCAGTGGGATGCCGGACATTACCGGACAACTGCTGCGGCACCTCAACTCCGATTTAATGAACGCAATATTCACAAGCAATGCGTGGTGTGCAACCAGCACAAAAGCGGAAATCTCGTTCCGTATCGCGTCGAACTGATTAGCCGCATCGGGCAGGAAGCAGTAGACGAAATCGAATCAAACCATAACCGCCATCGCTGGACTATCGAAGAGTGCAAGGCGATCAAGGCAGAGTACCAACAGAAACTCAAAGACCTGCGAAATAGCAGAAGTGAGGCCGCATGACGTTCTCAGTAAAAACCATTCCAGACATGCTCGTTGAAGCATACGGAAATCAGACAGAAGTAGCACGCAGACTGAAATGTAGTCGCGGTACGGTCAGAAAATACGTTGATGATAAAGACGGGAAAATGCACGCCATCGTCAACGACGTTCTCATGGTTCATCGCGGATGGAGTGAAAGAGATGCGCTATTACGAAAAAATTGATGGCAGCAAATACCGAAATATTTGGGTAGTTGGCGATCTGCACGGATGCTACACGAACCTGATGAACAAACTGGATACGATTGGATTCGACAACAAAAAAGACCTGCTTATCTCGGTGGGCGATTTGGTTGATCGTGGTGCAGAGAACGTTGAATGCCTGGAATTAATCACATTCCCCTGGTTCAGAGCTGTACGTGGAAACCATGAGCAAATGATGATTGATGGCTTATCAGAGCGTGGAAACGTTAATCACTGGCTGCTTAATGGCGGTGGCTGGTTCTTTAATCTCGATTACGACAAAGAAATTCTGGCTAAAGCTCTTGCCCATAAAGCAGATGAACTTCCGTTAATCATCGAACTGGTGAGCAAAGATAAAAAATATGTTATCTGCCACGCCGATTATCCCTTTGACGAATACGAGTTTGGAAAGCCAGTTGATCATCAGCAGGTAATCTGGAACCGCGAACGAATCAGCAACTCACAAAACGGGATCGTGAAAGAAATCAAAGGCGCGGACACGTTCATCTTTGGTCATACGCCAGCAGTGAAACCACTCAAGTTTGCCAACCAAATGTATATCGATACCGGCGCAGTGTTCTGCGGAAACCTAACATTGATTCAGGTACAGGGAGAAGGCGCATGAGACTCGAAAGCGTAGCTAAATTTCATTCGCCAAAAAGCCCGATGATGAGCGACTCACCACGGGCCACGGCTTCTGACTCTCTTTCCGGTACTGATGTGATGGCTGCTATGGGGATGGCGCAATCACAAGCCGGATTCGGTATGGCTGCATTCTGCGGTAAGCACGAACTCAGCCAGAACGACAAACAAAAGGCTATCAACTATCTGATGCAATTTGCACACAAGGTATCGGGGAAATACCGTGGTGTGGCAAAGCTTGAAGGAAATACTAAGGCAAAGGTACTGCAAGTGCTCGCAACATTCGCTTATGCGGATTATTGCCGTAGTGCCGCGACGCCGGGGGCAAGATGCAGAGATTGCCATGGTACAGGCCGTGCGGTTGATATTGCCAAAACAGAGCTGTGGGGGAGAGTTGTCGAGAAAGAGTGCGGAAGATGCAAAGGCGTCGGCTATTCAAGGATGCCAGCAAGCGCAGCATATCGCGCTGTGACGATGCTAATCCCAAACCTTACCCAACCCACCTGGTCACGCACTGTTAAGCCGCTGTATGACGCTCTGGTGGTGCAATGCCACAAAGAAGAGTCAATCGCAGACAACATTTTGAATGCGGTCACACGTTAGCAGCATGATTGCCACGGATGGCAACATATTAACGGCATGATATTGACTTATTGAATAAAATTGGGTAAATTTGACTCAACGATGGGTTAATTCGCTCGTTGTGGTAGTGAGATGAAAAGAGGCGGCGCTTACTACCGATTCCGCCTAGTTGGTCACTTCGACGTATCGTCTGGAACTCCAACCATCGCAGGCAGAGAGGTCTGCAAAATGCAATCCCGAAACAGTTCGCAGGTAATAGTTAGAGCCTGCATAACGGTTTCGGGATTTTTTATATCTGCACAACAGGTAAGAGCATTGAGTCGATAATCGTGAAGAGTCGGCGAGCCTGGTTAGCCAGTGCTCTTTCCGTTGTGCTGAATTAAGCGAATACCGGAAGCAGAACCGGATCACCAAATGCGTACAGGCGTCATCGCCGCCCAGCAACAGCACAACCCAAACTGAGCCGTAGCCACTGTCTGTCCTGAATTCATTAGTAATAGTTACGCTGCGGCCTTTTACACATGACCTTCGTGAAAGCGGGTGGCAGGAGGTCGCGCTAACAACCTCCTGCCGTTTTGCCCGTGCATATCGGTCACGAACAAATCTGATTACTAAACACAGTAGCCTGGATTTGTTCTATCAGTAATCGACCTTATTCCTAATTAAATAGAGCAAATCCCCTTATTGGGGGTAAGACATGAAGATGCCAGAAAAACATGACCTGTTGGCCGCCATTCTCGCGGCAAAGGAACAAGGCATCGGGGCAATCCTTGCGTTTGCAATGGCGTACCTTCGCGGCAGATATAATGGCGGTGCGTTTACAAAAACAGTAATCGACGCAACGATGTGCGCCATTATCGCCTGGTTCATTCGTGACCTTCTCGACTTCGCCGGACTAAGTAGCAATCTCGCTTATATAACGAGCGTGTTTATCGGCTACATCGGTACTGACTCGATTGGTTCGCTTATCAAACGCTTCGCTGCTAAAAAAGCCGGAGTAGAAGATGGTAGAAATCAATAATCAACGTAAGGCGTTCCTCGATATGCTGGCGTGGTCGGAGGGAACTGATAACGGACGTCAGAAAACCAGAAATCATGGTTATGACGTCATTGTAGGCGGAGAGCTATTTACTGATTACTCCGATCACCCTCGCAAACTTGTCACGCTAAACCCAAAACTCAAATCAACAGGCGCCGGACGCTACCAGCTTCTTTCCCGTTGGTGGGATGCCTACCGCAAGCAGCTTGGCCTGAAAGACTTCTCTCCGAAAAGTCAGGACGCTGTGGCATTGCAGCAGATTAAGGAGCGTGGCGCTTTACCTATGATTGATCGTGGTGATATCCGTCAGGCAATCGACCGTTGCAGCAATATCTGGGCTTCACTGCCGGGCGCTGGTTATGGTCAGTTCGAGCATAAGGCTGACAGCCTGATTGCAAAATTCAAAGAAGCGGGCGGAACGGTCAGAGAGATTGATGTATGAGCAGAGTCACCGCGATTATCTCCGCTCTGGTTATCTGCATCATCGTCTGCCTGTCATGGGCTGTTAATCATTACCGTGATAACGCCATTACCTACAAAGCCCAGCGCGACAAAAATGCCAGAGAACTGAAGCTGGCGAACGCGGCAATTACTGACATGCAGATGCGTCAGCGTGATGTTGCTGCGCTCGATGCAAAATACACGAAGGAGTTAGCTGATGCTAAAGCTGAAAATGATGCTCTGCGTGATGATGTTGCCGCTGGTCGTCGTCGGTTGCACATCAAAGCAGTCTGTCAGTCAGTGCGTGAAGCCACCACCGCCTCCGGCGTGGATAATGCAGCCTCCCCCCGACTGGCAGACACCGCTGAACGGGATTATTTCACCCTCAGAGAGAGGCTGATCACTATGCAAAAACAACTGGAAGGAACCCAGAAGTATATTAATGAGCAGTGCAGATAGAGTTGCCCATATCGATGGGCAACTCATGCAATTATTGTGAGCAATACACACGCGCTTCCAGCGGAGTATAAATGCCTAAAGTAATAAAACCGAGCAATCCATTTACGAATGTTTGCTGGGTTTCTGTTTTAACAACATTTTCTGCGCCGCCACAAATTTTGGCTGCATCGACAGTTTTCTTCTGCCCAATTCCAGAAACGAAGAAATGATGGGTGATGGTTTCCTTTGGTGCTACTGCTGCCGGTTTGTTTTGAACAGTAAACGTCTGTTGAGCACATCCTGTAATAAGCAGGGCCAGCGCAGTAGCGAGTAGCATTTTTTTCATGGTGTTATTCCCGATGCTTTTTGAAGTTCGCAGAATCGTATGTGTAGAAAATTAAACAAACCCTAAACAATGAGTTGAAATTTCATATTGTTAATATTTATTAATGTATGTCAGGTGCGATGAATCGTCATTGTATTCCCGGATTAACTATGTCCACAGCCCTGACGGGGAACTTCTCTGCGGGAGTGTCCGGGAATAATTAAAACGATGCACACAGGGTTTAGCGCGTACACGTATTGCATTATGCCAACGCCCCGGTGCTGACACGGAAGAAACCGGACGTTATGATTTAGCGTGGAAAGATTTGTGTAGTGTTCTGAATGCTCTCAGTAAATAGTAATGAATTATCAAAGGTATAGTAATATCTTTTATGTTCATGGATATTTGTAACCCATCGGAAAACTCCTGCTTTAGCAAGATTTTCCCTGT
ATTGCTGAAATGTGATTTCTCTTGATTTCAACCTATCATAGGACGTTTCTATAAGATGCG
TGTTTCTTGAGAATTTAACATTTACAACCTTTTTAAGTCCTTTTATTAACACGGTGTTAT
CGTTTTCTAACACGATGTGAATATTATCTGTGGCTAGATAGTAAATATAATGTGAGACGT
TGTGACGTTTTAGTTCAGAATAAAACAATTCACAGTCTAAATCTTTTCGCACTTGATCGA
ATATTTCTTTAAAAATGGCAACCTGAGCCATTGGTAAAACCTTCCATGTGATACGAGGGC
GCGTAGTTTGCATTATCGTTTTTATCGTTTCAATCTGGTCTGACCTCCTTGTGTTTTGTT
GATGATTTATGTCAAATATTAGGAATGTTTTCACTTAATAGTATTGGTTGCGTAACAAAG
TGCGGTCCTGCTGGCATTCTGGAGGGAAATACAACCGACAGATGTATGTAAGGCCAACGT
GCTCAAATCTTCATACAGAAAGATTTGAAGTAATATTTTAACCGCTAGATGAAGAGCAAG
CGCATGGAGCGACAAAATGAATAAAGAACAATCTGCTGATGATCCCTCCGTGGATCTGAT
TCGTGTAAAAAATATGCTTAATAGCACCATTTCTATGAGTTACCCTGATGTTGTAATTGC
ATGTATAGAACATAAGGTGTCTCTGGAAGCATTCAGAGCAATTGAGGCAGCGTTGGTGAA
GCACGATAATAATATGAAGGATTATTCCCTGGTGGTTGACTGATCACCATAACTGCTAAT
CATTCAAACTATTTAGTCTGTGACAGAGCCAACACGCAGTCTGTCACTGTCAGGAAAGTG
GTAAAACTGCAACTCAATTACTGCAATGCCCTCGTAATTAAGTGAATTTACAATATCGTC
CTGTTCGGAGGGAAGAACGCGGGATGTTCATTCTTCATCACTTTTAATTGATGTATATGC
TCTCTTTTCTGACGTTAGTCTCCGACGGCAGGCTTCAATGACCCAGGCTGAGAAATTCCC
GGACCCTTTTTGCTCAAGAGCGATGTTAATTTGTTCAATCATTTGGTTAGGAAAGCGGAT
GTTGCGGGTTGTTGTTCTGCGGGTTCTGTTCTTCGTTGACATGAGGTTGCCCCGTATTCA
GTGTCGCTGATTTGTATTGTCTGAAGTTGTTTTTACGTTAAGTTGATGCAGATCAATTAA
TACGATACCTGCGTCATAATTGATTATTTGACGTGGTTTGATGGCCTCCACGCACGTTGT
GATATGTAGATGATAATCATTATCACTTTACGGGCCGCGAATTCTCATGTTTGACCGCTT
ATCATCGATAAGCTCTGCTTTTTGTTGACTTCCATTGTTCATTCCACGGACAAAAACAGA
GAAAGGAAACGACAGAGGCCAAAAAGCTCGCTTTCAGCACCTGTCGTTTCCTTTCTTTTC
AGAGGGTATTTTAAATAAAAACATTAAGTTATGACGAAGAAGAACGGAAACGCCTTAAAC
CGGAAAATTTTCATAAATAGCGAAAACCCGCGAGGTCGCCGCCCCGTAACAAGGCGGATC
GCCGGAAAGGACCCGCAAATGATAATAATTATCAATTGCATACTATCGACGGCACTGCTG
CCAGATAACACCACCGGGGAAACATTCCATCATGATGGCCGTGCGGACATAGGAAGCCAG
TTCATCCATCGCTTTCTTGTCTGCTGCCATTTGCTTTGTGACATCCAGCGCCGCACATTC
AGCAGCGTTTTTCAGCGCGTTTTCGATCAACGTTTCAATGTTGGTATCAACACCAGGTTT
AACTTTGAACTTATCGGCACTGACGGTTACCTTGTTCTGCGCTGGCTCATCACGCAGGAT
ACCAAGGCTGATGTTGTAGATATTGGTCACCGGCTGAGGGTTTTCGATTGCCGCTGCGTG
GATAGCACCATTTGCGATCAGGCNGTCCTTGATGAATGACACTCCATTGCGAATAAGTTC
GAAGGAGACGGTGTCACGAATGCGCTGGTCCAGCTCGGTCGATTGCCTTTTGTGCAGCAG
AGGTATCAATCTCAACGCCAAGGCTCATCGAAGCGCAATATTGCTGCTCACCAAAACGCG
TATTGACCAGGTGTTCAACGGCAAATTTCTGCCCTTCTGATGTCAGAAAGGCAAAGTGAT
TTTCTTTCTGGTATTCAGTTGCTGTGTGTCGGTTTCAGCAAAACCAAGCTCGCGCAATTC
GGCTGTGCAGATTTAGAAGGCAGATCACCAGACAGCAACGGCCAACGGAAAACAGCGCAT
ACAGAACATCCGTCGCCGCGCCGACAACGTGATAATTTTTATGACCCATGATTTATTTCC
TTTTAGACGTGAGCCTGTCGCACAGCAAAGCCGCCGAAAGTTCCTCGACCGATGCCCTTG
AGAGCCTTCAACCCAGTCAGCTCCTTCCGGTGGGCGCGGGGCATGACTATCGTCGCCGCA
CTTATGACTGTCTTCTTTATCATGCAACTCGTAGGACAGGTGCCGGCAGCGCTCTGGGTC
ATTTTCGGCGAGGACCGCTTTCGCTGGAGCGCGACGATGATCGGCCTGTCGCTTGCGGTA
TTCGGAATCTTGCACGCCCTCGCTCAAGCCTTCGTCACTGGTCCCGCCACCAAACGTTTC
GGCGAGAAGCAGGCCATTATCGCCGGCATGGCGGCCGACGCGCTGGGCTACGTCTTGCTG
GCGTTCGCGACGCGAGGCTGGATGGCCTTCCCCATTATGATTCTTCTCGCTTCCGGCGGC
ATCGGGATGCCCGCGTTGCAGGCCATGCTGTCCAGGCAGGTAGATGACGACCATCAGGGA
CAGCTTCAAGGATCGCTCGCGGCTCTTACCAGCCTAACTTCGATCATTGGACCGCTGATC
GTCACGGCGATTTATGCCGCCTCGGCGAGCACATGGAACGGGTTGGCATGGATTGTAGGC
GCCGCCCTATACCTTGTCTGCCTCCCCGCGTTGCGTCGCGGTGCATGGAGCCGGGCCACC
TCGACCTGAATGGAAGCCGGCGGCACCTCGCTAACGGATTCACCACTCCAAGAATTGGAG
CCAATCAATTCTTGCGGAGAACTGTGAATGCGCAAACCAACCCTTGGCAGAACATATCCA
TCGCGTCCGCCATCTCCAGCAGCCGCACGCGGCGCATCTCGGGCAGCGTTGGGTCCTGCA
GATCCGGCTGTGGAATGTGTGTCAGTTAGGGTGTGGAAAGTCCCCAGGCTCCCCAGCAGG
CAGAAGTATGCAAAGCATGCATCTCAATTAGTCAGCAACCAGGTGTGGAAAGTCCCCAGG
CTCCCCAGCAGGCAGAAGTATGCAAAGCATGCATCTCAATTAGTCAGCAACCATAGTCCC
GCCCCTAACTCCGCCCATCCCGCCCCTAACTCCGCCCAGTTCCGCCCATTCTCCGCCCCA
TGGCTGACTAATTTTTTTTATTTATGCAGAGGCCGAGGCCGCCTCGGCCTCTGAGCTATT
CCAGAAGTAGTGAGGAGGCTTTTTTGGAGGCCTAGGCTTTTGCAAAAAGCTTCACGCTGC
CGCAAGCACTCAGGGCGCAAGGGCTGCTAAAGGAAGCGGAACACGTAGAAAGCCAGTCCG
CAGAAACGGTGCTGACCCCGGATGAATGTCAGCTACTGGGCTATCTGGACAAGGGAAAAC
GCAAGCGCAAAGAGAAAGCAGGTAGCTTGCAGTGGGCTTACATGGCGATAGCTAGACTGG
GCGGTTTTATGGACAGCAAGCGAACCGGAATTGCCAGCTGGGGCGCCCTCTGGTAAGGTT
GGGAAGCCCTGCAAAGTAAACTGGATGGCTTTCTTGCCGCCAAGGATCTGATGGCGCAGG
GGATCAAGATCTACGACCTGCATAACCAGTAAGAAGATAGCAGTGATGTCAAACGACGCA
GCTGACTTCTTTTCTTTCACGACTTCCCCACACCCAGCATGCATACCTTTCCGCCATAAC
TGTAGTGAATGTCTGTTATGAGCGAGGAGCGGAAGTTAACACTTATGAAAAATGGCTACG
AAGTCCGTGGCTATCTATCGGCTTATTAGTACTTGAAACGCTTCTTCAGAAGCCTGAAGA
GCTAATCGTTCGGCGATACTATATATGCATTAATAGACTATATCGTTGGTATAAACAGTG
CACCATGCAACATGAATAACAGTGGGTTATCCAAAAGGAAGCAGAAAGCTAAATATGGAA
AACTACAATACGATGCCCCGTTAAGTTCAATACTACTAATTTTTAGATGGAAAACGTATG
TAATAGAGAGTAACTTAAAAGAGAGATCCTGTGTTGCCGCCAAATAAATTGCGGTTATTT
TAATAAAATTAAGGGTTACTATATGTTGGAGTTTAGTGTTATTGAAAGAGGCGGGTATAT
TCCTGCAGTAGAAAAAAATAAGGCATTCCTACGAGCAGATGGTTGGAATGACTATTCCTT
TGTTACAATGTTTTATCTTACTGTCTTTGATGAGCATGGTGAAAAATGCGATATCGGAAA
TGTTAAAATTGGTTTTGTAGGTCAAAAAGAAGAAGTAAGCACTTATTCATTAATAGATAA
AAAATTCAGTCAACTCCCTGAAATGTTTTTTTCCTTAGGTGAAAGCATTGACTACTATGT
TAATCTCAGCAAATTAAGCGATGGTTTTAAACATAACCTTCTTAAAGCTATTCAGGATTT
AGTAGTATGGCCAAATCGATTAGCCGACATTGAAAATGAAAGCGTCCTTAACACCTCATT
ACTTAGAGGGGTAACTCTTTCAGAAATTCATGGACAGTTCGCACGTGTGTTAAATGGTTT
GCCAGAATTGTCAGATTTCCACTTTTCATTTAATAGAAAAAGTGCTCCCGGATTCAGTGA
TTTAACTATACCTTTTGAGGTGACGGTTAATTCTATGCCCAGCACGAACATTCATGCTTT
TATCGGGCGGAATGGGTGTGGTAAAACAACAATTTTGAATGGAATGATTGGTGCAATCAC
CAACCCAGAAAACAATGAATATTTTTTCTCTGAAAATAATAGACTTATCGAGTCAAGAAT
CCCAAAGGGATATTTTCGATCGCTTGTTTCAGTTTCGTTTAGTGCATTTGATCCTTTTAC
TCCTCCTAAAGAACAACCTGACCCAGCAAAAGGTACACAATACTTTTATATTGGACTCAA
GAATGCTGCCAGCAATAGTTTAAAATCACTAGGCGATCTCCGCTTAGAATTCATTTCAGC
ATTTATTGGTTGTATGAGAGTAGATAGAAAAAGACAACTCTGGCTTGAAGCTATCAAAAA
ACTAAGTAGTGATGAAAACTTTTCAAATATGGAACTCATCAGCCTCATTTCTAAATATGA
AGAGTTAAGACGTAATGAACCACAGATTCAAGTGGACGATGATAAATTCACTAAATTGTT
TTATGACAATATCCAGAAATATCTGCTTCGAATGAGCTCTGGACATGCAATTGTTTTATT
TACTATCACAAGATTAGTAGATGTCGTTGGCGAAAAGTCATTAGTTTTATTCGATGAACC
AGAGGTTCATCTGCATCCACCTTTGCTCTCTGCTTTTTTACGAACATTAAGCGACTTACT
CGATGCACGCAATGGTGTAGCAATAATTGCAACTCATTCCCCAGTAGTACTGCAAGAGGT
TCCAAAATCCTGCATGTGGAAAGTCCTACGGTCAAGAGAAGCAATAAATATTATCCGTCC
GGATATTGAGACATTCGGTGAGAACTTAGGTGTTTTAACTCGTGAGGTGTTTTTACTTGA
AGTGACAAATTCTGGATACCACCACTTATTATCGCAGTCCGTTGATTCAGAGCTTTCTTA
TGAAACCATTCTAAAAAATTATAATGGTCAGATAGGATTAGAAGGTCGAACCGTTTTAAA
AGCGATGATAATGAACAGAGATGAAGGTAAAGTACAATGAAAAAACTACCTCTTCCAGCG
AGAACTTATAGCGAAATGCTTAATAAATGCTCGGAAGGTATGATGCAGATAAATGTTAGA
AATAATTTCATTACTCACTTCCCCACTTTTTTGCAGAAAGAACAACAATATAGAATATTA
AGCTCGACAGGTCAGTTATTTACCTACGACAGGACACACCCTCTTGAGCCTACAACCTTA
GTAGTTGGTAACCTGACAAAGGTTAAATTAGAAAAGCTTTATGAAAATAATCTCCGAGAT
AAAAACAAACCCGCTAGAACATATTACGATGACATGCTTGTTTCATCAGGTGAAAAATGT
CCATTTTGTGGTGATATAGGACAGACAAAAAATATAGATCATTTTCTTCCTATTGCACAT
TATCCTGAATTTTCGGTGATGCCTATTAATTTAGTTCCATCGTGCCGCGACTGCAATATG
GGAGAGAAAGGTCAAGTTTTCGCAGTAGATGAGGTACACCAAGCGATTCATCCCTATATC
GACAAGGACATTTTTTTTCGTGAGCAATGGGTATATGCAAATTTCGTTTCCGGAACTCCG
GGTGCTATCAGTTTTTATGTTGAATGCCCGGCGAACTGGAGGCAGGAAGACAAACACAGA
GCTCTTCATCATTTCAAGCTATTAAATATTGCTAACAGGTATCGTTTGGAGGCAGGGAAG
CACTTGAGTGAAGTGATTACTCAAAGAAACTCTTTCGTAAAAGTTATAAGGAAATATAGT
TCAACCGCAACGTTTCAGCAGCTACAGTCAGAATTTATTGAAGCAAATCTGAAACCTATT
ATAGATTTGAATGACTTCCCCAATTATTGGAAAAGAGTTATGTATCAGTGCCTAGCAAAC
TCGGAAGATTTTTTCAGAGGGATCTAGAATATGATGAAAGATAGAAAATTACGACGCTTA
TCGGAAGTGAACGAATACTTTTTATATGAGGAGGGCTGTTTTTACAAAATCCGGTAGTAA
CTTGCTAACCAATTCCTAGGCAGGTCATTGGCAACAGTGGCATGCACCGAGAAGGACGTT
TGTAATGTCCGCTCCGGCACATAGCAGTCCTAGGGACAGTGGCGTACAGTCATAGATGGT
CGGTGGGAGGTGGTACAAATTCTCTCATGCAAAAAATATGTAAAATCGGTAGCAACTGGA
AATCATTCAACACCCGCACTATCGGAAGTTCACCAGCCAGCCGCAGCACGTTCCTGCATA
CGACGTGTCTGCGGCTCTACCATATCTCCTATGAGCAACGTGTTAGCAGAGCCAAGCCAC
AACTCTAATTTTAATACATAATGAATGATAATAATAATATTAAAAATTTCCTGTGTAACT
AATTTACTATATGGTTTCTGATAAGAATCATTGCAAAGATCAAACAACTTGTATTACATT
GACAGTTAAGCAGTTAATTTTATCACCTCTAAAATATATCAGCATCTAGCATGCAACCTA
TCAAAATGGAGAGTTTTATGACTAAAAAACCATGGGAAAGAAGACTTAAAGATTTATCGC
ACTTGCTCAAATGCTGCATTGATACATATTTTGACCCTGAATTATTTCGCTTGAATTTGA
ATCAATTCCTCCAAACCGCAAGAACAGTAACATTTATTATTCAAAAAAACAAAAACCAGA
TTATAGGATATGACATTTGGTATAACAATAATGTTATTGAAAAATGGAAAAATGATCCAT
TAATGGCTTGGGCTAAAAATTCTCGCAATACGATAGAAAAACAAGGCGATTTAGAAATGT
ATAGCGAGGCAAAGGCTACTCTTATTTCATCTTACATTGAAGAAAATGACATTGAGTTTA
TTACAAATGAAAGTATGTTAAACATTGGTATAAAAAAGTTAGTCAGACTTGCACAAAAGA
AATTACCTTCATATTTAACTGAATCATCTATTATTAAATCAGAAAGACGATGGGTCGCTA
ATACGCTAAAAGATTACGAATTATTACATGCCTTAGCTATAATCTATGGCAGAATGTATA
ACTGCTGTAACTCTCTTGGCATACAAATAAACAATCCAATGGGTGACGATGTGATTTCGC
CAACATCATTCGACTCTTTATTTGATGAAGCCAGGAGAATAACTTATTTAAAATTAAAAG
ATTACTCCATAAGCAAATTGTCATTTAGCATGATACAATATGACAATAAAATAATTCCTG
AAGATATTAAAGAGCGTCTAAAACTGGTAGATAAGCCTAAAAATATCACTTCGACAGAAG
AGTTAGTTGACTATACAGCCAAGCTTGCAGAAACGACTTTTTTAAAGGACGGTTATCACA
TTCAAACATTAATTTTTTATGATAAACAATTCCATCCAATTGATTTAATCAATACAACAT
TTGAAGATCAAGCAGATAAATATATTTTTTGGCGTTATGCAGCTGACAGAGCCAAAATAA
CAAATGCCTATGGCTTCATTTGGATATCAGAGCTATGGCTCAGAAAAGCAAGCATCTACT
CCAATAAACCAATACATACAATGCCAATTATAGATGAAAGACTTCAGGTAATTGGAATTG
ATTCAAATAATAATCAAAAATGTATTTCATGGAAAATAGTTAGAGAAAACGAAGAAAAAA
AACCGACTTTAGAAATATCAACAGCAGACTCAAAACATGACGAAAAACCATATTTCATGC
GTTCAGTCTTAAAAGCAATTGGCGGTGATGTAAACACTATGAACAATTGAGTCATAGAAC
TTCCATTATTCTCCTGAAGATAATAATCGCCAAATAAACCAATACTCAGCTTTACAATAT
ACTAACTAACCGCAGAACGTTATTTCATACAACGTTTCTGCGGCATATCACAAAACGATT
ACTCCATAACAGGGACAGCAGGCCACTCAATATCAGGTGCAGTTGATGTATCAAGCGCGC
ATGCCCGACGGCGAGGATCTCGTCGTGACCCATGGCGATGCCTGCTTGCCGAATATCATG
GTGGAAAATGGCCGCTTTTCTGGATTCATCGACTGTGGCCGGCTGGGTGTGGCGGACCGC
TATCAGGACATAGCGTTGGCTACCCGTGATATTGCTGAAGAGCTTGGCGGCGAATGGGCT
GACCGCTTCCTCGTGCTTTACGGTATCGCCGCTCCCGATTCGCAGCGCATCGCCTTCTAT
CGCCTTCTTGACGAGTTCTTCTGAGCGGGACTCTGGGGTTCGAAATGACCGACCAAGCGA
CGCCCAACCTGCCATCACGAGATTTCGATTCCACCGCCGCCTTCTATGAAAGGTTGGGCT
TCGGAATCGTTTTCCGGGACGCCGGCTGGATGATCCTCCAGCGCGGGGATCTCATGCTGG
AGTTCTTCGCCCACCCCGGGCTCGATCCCCTCGCGAGTTGGTTCAGCTGCTGCCTGAGGC
TGGACGACCTCGCGGAGTTCTACCGGCAGTGCAAATCCGTCGGCATCCAGGAAACCAGCA
GCGGCTATCCGCGCATCCATGCCCCCGAACTGCAGGAGTGGGGAGGCACGATGGCCGCTT
TGGTCCGGATCTTTGTGAAGGAACCTTACTTCTGTGGTGTGACATAATTGGACAAACTAC
CTACAGAGATTTAAAGCTCTAAGGTAAATATAAAATTTTTAAGTGTATAATGTGTTAAAC
TACTGATTCTAATTGTTTGTGTATTTTAGATTCCAACCTATGGAACTGATGAATGGGAGC
AGTGGTGGAATGCCTTTAATGAGGAAAACCTGTTTTGCTCAGAAGAAATGCCATCTAGTG
ATGATGAGGCTACTGCTGACTCTCAACATTCTACTCCTCCAAAAAAGAAGAGAAAGGTAG
AAGACCCCAAGGACTTTCCTTCAGAATTGCTAAGTTTTTTGAGTCATGCTGTGTTTAGTA
ATAGAACTCTTGCTTGCTTTGCTATTTACACCACAAAGGAAAAAGCTGCACTGCTATACA
AGAAAATTATGGAAAAATATTCTGTAACCTTTATAAGTAGGCATAACAGTTATAATCATA
ACATACTGTTTTTTCTTACTCCACACAGGCATAGAGTGTCTGCTATTAATAACTATGCTC
AAAAATTGTGTACCTTTAGCTTTTTAATTTGTAAAGGGGTTAATAAGGAATATTTGATGT
ATAGTGCCTTGACTAGAGATCATAATCAGCCATACCACATTTGTAGAGGTTTTACTTGCT
TTAAAAAACCTCCCACACCTCCCCCTGAACCTGAAACATAAAATGAATGCAATTGTTGTT
GTTAACTTGTTTATTGCAGCTTATAATGGTTACAAATAAAGCAATAGCATCACAAATTTC
ACAAATAAAGCATTTTTTTCACTGCATTCTAGTTGTGGTTTGTCCAAACTCATCAATGTA
TCTTATCATGTCTGGATCTGACGGGTGCGCATGATCGTGCTCCTGTCGTTGAGGACCCGG
CTAGGCTGGCGGGGTTGCCTTACTGGTTAGCAGAATGAATCACCGATACGCGAGCGAACG
TGAAGCGACTGCTGCTGCAAAACGTCTGCGACCTGAGCAACAACATGAATGGTCTTCGGT
TTCCGTGTTTCGTAAAGTCTGGAAACGCGGAAGTCAGCGCTCTTCCGCTTCCTCGCTCAC
TGACTCGCTGCGCTCGGTCGTTCGGCTGCGGCGAGCGGTATCAGCTCACTCAAAGGCGGT
AATACGGTTATCCACAGAATCAGGGGATAACGCAGGAAAGAACATGTGAGCAAAAGGCCA
GCAAAAGGCCAGCAAAAGGCCAGGAACCGTAAAAAGGCCGCGTTGCTGGCGTTTTTCCAT
AGGCTCCGCCCCCCTGACGAGCATCACAAAAATCGACGCTCAAGTCAGAGGTGGCGAAAC
CCGACAGGACTATAAAGATACCAGGCGTTTCCCCCTGGAAGCTCCCTCGTGCGCTCTCCT
GTTCCGACCCTGCCGCTTACCGGATACCTGTCCGCCTTTCTCCCTTCGGGAAGCGTGGCG
CTTTCTCATAGCTCACGCTGTAGGTATCTCAGTTCGGTGTAGGTCGTTCGCTCCAAGCTG
GGCTGTGTGCACGAACCCCCCGTTCAGCCCGACCGCTGCGCCTTATCCGGTAACTATCGT
CTTGAGTCCAACCCGGTAAGACACGACTTATCGCCACTGGCAGCAGCCACTGGTAACAGG
ATTAGCAGAGCGAGGTATGTAGGCGGTGCTACAGAGTTCTTGAAGTGGTGGCCTAACTAC
GGCTACACTAGAAGGACAGTATTTGGTATCTGCGCTCTGCTGAAGCCAGTTACCTTCGGA
AAAAGAGTTGGTAGCTCTTGATCCGGCAAACAAACCACCGCTGGTAGCGGTGGTTTTTTT
GTTTGCAAGCAGCAGATTACGCGCAGAAAAAAAGGATCTCAAGAAGATCCTTTGATCTTT
TCTACGGGGTCTGACGCTCAGTGGAACGAAAACTCACGTTAAGGGATTTTGGTCATGAGA
TTATCAAAAAGGATCTTCACCTAGATCCTTTTAAATTAAAAATGAAGTTTTAAATCAATC
TAAAGTATATATGAGTAAACTTGGTCTGACAGTTACCAATGCTTAATCAGTGAGGCACCT
ATCTCAGCGATCTGTCTATTTCGTTCATCCATAGTTGCCTGACTCCCCGTCGTGTAGATA
ACTACGATACGGGAGGGCTTACCATCTGGCCCCAGTGCTGCAATGATACCGCGAGACCCA
CGCTCACCGGCTCCAGATTTATCAGCAATAAACCAGCCAGCCGGAAGGGCCGAGCGCAGA
AGTGGTCCTGCAACTTTATCCGCCTCCATCCAGTCTATTAATTGTTGCCGGGAAGCTAGA
GTAAGTAGTTCGCCAGTTAATAGTTTGCGCAACGTTGTTGCCATTGCTGCAGGCATCGTG
GTGTCACGCTCGTCGTTTGGTATGGCTTCATTCAGCTCCGGTTCCCAACGATCAAGGCGA
GTTACATGATCCCCCATGTTGTGCAAAAAAGCGGTTAGCTCCTTCGGTCCTCCGATCGTT
GTCAGAAGTAAGTTGGCCGCAGTGTTATCACTCATGGTTATGGCAGCACTGCATAATTCT
CTTACTGTCATGCCATCCGTAAGATGCTTTTCTGTGACTGGTGAGTACTCAACCAAGTCA
TTCTGAGAATAGTGTATGCGGCGACCGAGTTGCTCTTGCCCGGCGTCAACACGGGATAAT
ACCGCGCCACATAGCAGAACTTTAAAAGTGCTCATCATTGGAAAACGTTCTTCGGGGCGA
AAACTCTCAAGGATCTTACCGCTGTTGAGATCCAGTTCGATGTAACCCACTCGTGCACCC
AACTGATCTTCAGCATCTTTTACTTTCACCAGCGTTTCTGGGTGAGCAAAAACAGGAAGG
CAAAATGCCGCAAAAAAGGGAATAAGGGCGACACGGAAATGTTGAATACTCATACTCTTC
CTTTTTCAATATTATTGAAGCATTTATCAGGGTTATTGTCTCATGAGCGGATACATATTT
GAATGTATTTAGAAAAATAAACAAATAGGGGTTCCGCGCACATTTCCCCGAAAAGTGCCA
CCTGACGTCTAAGAAACCATTATTATCATGACATTAACCTATAAAAATAGGCGTATCACG
AGGCCCTTTCGTCTTCAAGAATTCGC
