# Supplementary material for: Quantitative parameters of bacterial RNA polymerase open-complex formation, stabilization and disruption on a consensus promoter
Source: Nucleic Acids Res. 2022 Jul 12;50(13):7511–28. doi: 10.1093/nar/gkac560 (PMC9303404; doi:10.1093/nar/gkac560)
Supplement: gkac560_Supplemental_Files [file gkac560_supplemental_files.zip › Supplementary Data 2.rtf]

>1.4kb tether
AACAAATCTATTATACCAATCGGCTTCAACAATGGTGCTCCACGGCAGGCGCCTGACGAGAGGACCAACACACCGAGGAACCTCAGCGCTTCGACATGGCAAAATCCCCCCCTTGCAACTTCTAGAGGAGAAGAGTACTGACTTGAGCGCTCCCAGCACAACAAGGATAGACTCCTCAGCGTTGCACGTTGGGGATAGCGCTAGCTAACAAAGACGCCTGCTACAACAGGAGTATCAAACCCGTACAAAGGGAACATCCACACTCCTCAGCTTGGTGAATCGAAGCGCGGCATCAGGATTTCCTTTTGGATACCTGAAACAAAGCCCATCGTGGTCCTGTAGACTTGGCACACTCCTCAGCTACACCTGCAGCGCGCGCATGTTATAGCATCAGGCAGTTTAAGTCGGGACAATAGGGGCCGCAATACACAGTTTACCGCATCCTCAGCCTTGACCTAACTGACAAACTGCCATGGACGACTAGCCATGCTCTTAGACAGCCCGTCTCATACAGTGATTATGGTCTCGAATTGTCCTCAGCTTGCAGTGAGCGCAACGCAATAAATGTGATCTAGATCACATTTTAGGCACCCCAGGCTTGACACTTTATGCTTCGGCTCGTATAATGTGTGGAATTGTGAGAGCGGAAGGACCTCAGCATTAGAGTTCAATAAGGTCTCCTACCAAGCAACTCAGAGATCTCACAGGCTTAGAAGACCATCAATCTCCCCTCAGCAGACAGGCCTCCTGTTAAGATGGCAGAGCCCGGTAATCGCACTCAAGACACATAGACTAGTATTCAGGCCTGCTGGTAATCGCAGGCCTTTTTATTTGGGCGGGCCTCAGCGCATGGCTAACTTGAATTCCTACGTGCGAGGGCAGAAGACTTATCCGCATTTCGTCTCTTCACCTATCTACTACCCATGCCCCTCAGCCGGAGATTATGTAGGTTGTGAGATGCGGGAGAGGTTCTCGATCTTCCCGTGGGACGTCAACCTTTCCCTTGATAAAGCATTCCCTCAGCCGCTCGGGTATGGCAGTAAGTACGCCTTCTGAATTGTGCTAACCTTCATCCTTATCAAGGCTTGCTGCCAATGATTAGGATTCCTCAGCATTGCCTTGCGACAGACTTCCTACTCACACTCGCTCACATTGAGCTACTCGATGGGCCATCAGCTTGACCCGCTCTGTAGGGCCTCAGCTCGCGATTACGTGAGTTAGGGCTCCGGACTGCGCTGTATAGTCGAATCTGATCTCGCCCCAACAACTGCAAACCCCAACTTACCTCAGCTTTAGATAACATGATTAGCCGAAGTTGCACGGGGTGCCCACCGTGGACTCCTCCCCGGGTGTCGCTCCTTCATCTGACAATATCCTCAGCGCAGCCGCTACCACCATCG
